# Bayesian Estimation of Correlation between Measures of Blood Pressure Indices, Aerobic Capacity and Resting Heart Rate Variability Using Markov Chain Monte Carlo Simulation and 95% High Density Interval in Female School Teachers

**DOI:** 10.3390/ijerph17186750

**Published:** 2020-09-16

**Authors:** Shaher A. I. Shalfawi

**Affiliations:** Department of Education and Sports Science, University of Stavanger, 4036 Stavanger, Norway; shaher.shalfawi@uis.no

**Keywords:** psychophysiology health, rate-pressure product, mean arterial blood pressure, MCMC, data simulation

## Abstract

Background: Several explanations regarding the disparity observed in the literature with regard to heart rate variability (HRV) and its association with performance parameters have been proposed: the time of day when the recording was conducted, the condition (i.e., rest, active, post activity) and the mathematical and physiological relationships that could have influenced the results. A notable observation about early studies is that they all followed the frequentist approach to data analyses. Therefore, in an attempt to explain the disparity observed in the literature, the primary purpose of this study was to estimate the association between measures of HRV indices, aerobic performance parameters and blood pressure indices using the Bayesian estimation of correlation on simulated data using Markov Chain Monte Carlo (MCMC) and the equal probability of the 95% high density interval (95% HDI). Methods: The within-subjects with a one-group pretest experimental design was chosen to investigate the relationship between baseline measures of HRV (rest; independent variable), myocardial work (rate–pressure product (RPP)), mean arterial pressure (MAP) and aerobic performance parameters. The study participants were eight local female schoolteachers aged 54.1 ± 6.5 years (mean ± SD), with a body mass of 70.6 ± 11.5 kg and a height of 164.5 ± 6.5 cm. Their HRV data were analyzed in R package, and the Bayesian estimation of correlation was calculated employing the Bayesian hierarchical model that uses MCMC simulation integrated in the JAGS package. Results: The Bayesian estimation of correlation using MCMC simulation reproduced and supported the findings reported regarding norms and the within-HRV-indices associations. The results of the Bayesian estimation showed a possible association (regardless of the strength) between pNN50% and MAP (*rho* = 0.671; 95% HDI = 0.928–0.004), MeanRR (ms) and RPP (*rho* = −0.68; 95% HDI = −0.064–−0.935), SDNN (ms) and RPP (*rho* = 0.672; 95% HDI = 0.918–0.001), LF (ms^2^) and RPP (*rho* = 0.733; 95% HDI = 0.935–0.118) and SD2 and RPP (*rho* = 0.692; 95% HDI = 0.939–0.055). Conclusions: The Bayesian estimation of correlation with 95% HDI on MCMC simulated data is a new technique for data analysis in sport science and seems to provide a more robust approach to allocating credibility through a meaningful mathematical model. However, the 95% HDI found in this study, accompanied by the theoretical explanations regarding the dynamics between the parasympathetic nervous system and the sympathetic nervous system in relation to different recording conditions (supine, reactivation, rest), recording systems, time of day (morning, evening, sleep etc.) and age of participants, suggests that the association between measures of HRV indices and aerobic performance parameters has yet to be explicated.

## 1. Introduction

Heart rate variability (HRV) research has been intensified due to the significant relationship observed between cardiovascular mortality and the autonomic nervous system [1,2]. This has led to establishing a work group called the “Task Force”, involving scientists and researchers from both the European Society of Cardiology and the North American Society for Pacing and Electrophysiology in 1996 [3] with the aim of developing the standards for HRV research. The objectives of the task force were to develop the definitions and terms of HRV, identify standard measurement methods, define how HRV correlates with human physiology, describe how the measures are practiced clinically and identify needed research [3]. A search on pubmed.gov using the term “heart rate variability” yielded 39,743 results for studies investigating HRV from a wide variety of disciplines since the task force report was published to date. The HRV is a reflection of the change in the time interval between successive heartbeats [1,3,4]. This inter-beat interval (IBI) has been shown to be influenced by the autonomic nervous system and more specifically, parasympathetic nervous system (PNS) activities and sympathetic nervous system (SNS) activities [3,5,6]. This is because the heart is controlled by both the higher brain center and the cardiovascular control area [1,7]. The PNS is believed to be mediated by the fluctuations of vagal-cardiac nerve [1] and its activity is higher during rest, which contributes to slower heart rate observed in healthy people, and is associated with a respiratory frequency of about 0.15 to 0.4 Hz [1,3,6,7]. The SNS activities have been reported to increase with increased activity (stress) and corresponds to a respiratory frequency range between 0.04 and 0.15 Hz [1,3,4,6,7]. Therefore, the interaction between the two frequencies suggests that the low frequency (i.e., 0.04 and 0.15 Hz) represents both SNS and PNS branches [3,4,6,7]. Due to this dynamic relationship, it is expected that PNS activities (depending on the physical and psychological state of the person) may or may not be associated with SNS activities [6]. Therefore, researchers have pointed out the importance of being careful when interpreting HRV results [3,4,6,8].

The task force report in 1996 was the first robust guide for conducting HRV research [3]. Nevertheless, the task force left many elements regarding the research process open for other researchers to develop. After this report, Berntson et al. [1] were the first to provide the most robust guidelines regarding HRV research process. Thereafter, researchers provided improved guidelines to follow [4,5,9,10]. All these studies provided guides regarding the HRV data collection, analyses and cleaning, calculation and reporting [1,3,4,5,8,9,10]. However, the studies agreed that HRV can be analyzed using three domains: the first is based on the statistical method concerned with IBI time series (i.e., time domain); the second is the frequency domain, reported to divide the heart rhythm oscillations into 4 bands; the third is the nonlinear indices (Poincaré plot) (Table 1).

The availability of recording systems and techniques such as photoplethysmography [11], IBI recording [12] and (gold standard) electrocardiography (ECG) [3,4,7] made the recording of HRV noninvasive and accessible. The advancement in technology allowed researchers to use accessible systems, such as heart rate monitors, to record IBI to be able to assess their athletes’ HRV. For example, in 2005, Kingsley et al. [12] compared Polar 810s with Reynolds digital ambulatory ECG. They found that Polar 810s produced similar results to the ECG recording during rest and exercise at any relative intensity. In 2009, Nunan et al. [13] reinvestigated the validity and reliability of the Polar 810s using simultaneous recording with CardioPerfect 12-lead ECG module in the resting supine position. Their results indicated a high validity and reliability of the Polar 810s. In 2016, Hernando et al. [14] validated the Polar RS800 for HRV analysis during rest and exercise, the results of the study showing a valid and reliable recording during rest with some variation during exercise. Similar results were reported in 2017 by Cassirame et al. [15], who conducted a study examining the accuracy of the Garmin 920XT HRM compared to the standard ECG recording. They did not report differences in HRV analysis in resting supine position, whereas differences were observed in the exercise condition. These studies confirm the importance of carefully choosing the recording conditions in relation to the purpose of the study for the results to be comparable [3,4].

The ventricular contraction and relaxation are a continues process in a cardiac cycle and the systolic and diastolic blood pressure (BP) are measured during these contractions and relaxations, respectively [10]. The heart continuously pumps blood to different parts of the body and the rate of pumps per minute depends on the body need (i.e., relaxing, during exercise, before and after meals, standing, walking etc.). Body needs have been categorized into “physiological and pathological, neuropsychological, lifestyle, non-modifiable and environmental factors [16].” However, the heart muscle is influenced by the medulla, which is responsible for transmitting the signals coming from the PNS and SNS to the heart [17]. Research shows that an increase in SNS activities increases heart rate, and an increase in PNS activities slows heart rate [1,3,10,17]. Therefore, the ventricular contraction and relaxation is connected to relatively balanced PNS and SNS activities, which are linked to the HRV indices. This can be illustrated by the process of inhaling and exhaling during breathing; during inhaling, heart rate (HR) increases and BP rises and vice versa during exhaling [6]. Hence, the change in BP is continuous and indicates that the body systems function as a regulator to maintain a homeostasis state [18]. Therefore, an increase in BP would implicate a decrease in heart rate and vascular tone and vice versa [6]. This relationship between PNS and SNS (i.e., the source of HRV) and BP has been extensively studied and is well documented [6,10,16,18,19,20,21,22,23,24]. Since HR depends on the person state and the IBI varies accordingly, it was expected that HRV indices would be related to the ability of the heart to pump the needed amount of blood per minute to carry the needed amount of oxygen and deliver it to the working tissues via the cardiovascular system [25]. Hence, several researchers investigated this relationship, and a brief review of the published reports indicates a disparity in the reported results (Table 2).

Several explanations regarding the disparity observed (Table 2) in the literature could be hypothesized; among others, the time of day when the recording was conducted and the condition (i.e., rest, active, post activity). Vitale et al. [39] concluded that the human circadian rhythms could potentially differ during the time of day. The task force indicated that mathematical and physiological relationships could influence the results [3]. Observing all the studies presented earlier, one can note that all mentioned studies followed the frequentist approach to data analyses. To the best of the author’s knowledge, no studies to date have investigated the relationship between measures of HRV indices and aerobic capacity using the Bayesian approach to data analyses. Therefore, in an attempt to explain the disparity observed in the literature, the primary purpose of this study was to estimate the association between measures of HRV indices, aerobic performance and blood pressure indices employing the Bayesian estimation of correlation and the equal probability of the 95% high density interval (HDI) on a simulated data. To assure the suitability of the data, a secondary aim was to compare the data produced to the norms and to investigate the estimation of the association within the HRV indices in comparison to those reported.

## 2. Materials and Methods

### 2.1. Study Design

It is well documented that BP and respiration affect the measurements of HRV [5,9]. To avoid the interindividual differences in HRV measures caused by BP and respiration, the within-subjects experimental design has been shown to be the optimal experimental design in HRV studies, giving a further increased statistical power when using a small sample size [4,5,9]. Therefore, this study followed these recommendations, and the within-subjects with a one-group pretest experimental design was chosen to investigate the relationship between baseline measures of HRV (rest; independent variable), myocardial work (rate–pressure product (RPP)), mean arterial pressure (MAP) and aerobic capacity performances on female schoolteachers over 30 years old [4,9].

### 2.2. Participants

Twelve local female schoolteachers aged 53.9 ± 5.7 years (mean ± SD), with a body mass of 71.8 ± 10.1 kg and a height of 164.4 ± 6.2 cm, volunteered to participate in the present study. To be able to compare the results with the norms reported in the literature [40], the participants had to be females ≥30 years old, not consume medication that might influence their results at the time of the study, be free from injuries and illness and complete the testing procedure. After the initial consultation with the participants, two participants reported that they were on BP medication; therefore, those two participants’ measures were excluded from the study. Furthermore, two participants did not complete the testing procedure, leaving this study with 8 female schoolteachers aged 54.1 ± 6.5 years (mean ± SD), with a body mass of 70.6 ± 11.5 kg and a height of 164.5 ± 6.5 cm. In addition to their everyday teaching at schools, the participants conducted circuit training with the same instructor twice a week. Written informed consent was obtained from all participants after a verbal and written explanation of the experimental design and potential risks associated with participating in the study. The study was conducted in accordance with the Helsinki Declaration and the Norwegian National Committees for Research Ethics. This study was approved by the Norwegian center for research data (id: 738807).

### 2.3. Procedure and Instruments

#### 2.3.1. Anthropometry

Participants were asked not to have intense physical training the day before the experiment day and not to consume food for a minimum of 2 h prior to the testing. Then, upon their admission to the laboratory, height and body mass were recorded using a wall mounted Seca stadiometer model 222 and Seca flat digital scale model 877, respectively (Seca Medical Measuring Systems and Scales, Hamburg, Germany).

#### 2.3.2. Blood Pressure

Blood pressure was measured manually using a Reister stethoscope (model: Anestophon) and sphygmomanometer (model: Big Ben; Rudolf Riester GmbH, Jungingen, Germany). All BP instruments were tested and approved by the British and Irish Hypertension Society (BHS) Validation Service [41]. Blood pressure was assessed according to the recommendations described in Kallioinen et al. [42]. Systolic and diastolic pressures together with HR were noted. Baseline RPP (RPP = heart rate x systolic arterial pressure) and MAP (MAP = ((Systolic blood pressure − Diastolic blood pressure) ÷ 3) + Diastolic blood pressure) were then estimated [19,24].

#### 2.3.3. HRV Data Acquisition

Garmin 920XT (Garmin Ltd., Olathe, KS, USA) was used to record HRV in the resting supine position prior to the aerobic capacity test. The Garmin 920XT has been reported to have high HRV accuracy compared to ECG measurements when recording at rest in the supine position [15]. Prior to measuring HRV, Garmin HRM-Tri was placed around the center of the chest and below the level of the breasts. Garmin HRM-Tri uses ANT+ technology to transfer heart rate measures to the Garmin 920XT using a 2.4 GHz ANT wireless communication protocol. To measure Short-Term HRV (5 min), the Garmin 920XT was preprogramed to record for 10 min. The 10 min recording was chosen to ensure that the participants had enough time to acclimatize to the recording environment [4], and to record enough data points to be able achieve a clean 5 min of HRV recording [1,3,43]. To ensure comparability of results across studies and laboratories, the short-term (5 min) recording was adopted in line with the task force recommendations [3].

#### 2.3.4. Aerobic Capacity Test

The mask size for each participant was chosen to insure headspace correction, and the Vyntus CPX gas analyzer (Model: versatile JAEGER; Vyaire medical, Hoechberg, Germany) was calibrated using the fully automated 2-point gas calibration of the O_2_/CO_2_, through a special Twin Tube sample line combined with a fresh air flush system [44]. The participants were tested on a motorized treadmill (Ergo ELG 55) that was connected to a programmable external WOODWAY User-System version 2.0 (Woodway GmbH, Weilam Rhein, Germany). The modified Bruce continuous incremental test protocol was used to test the participants [45]. The test continued until the participant could no longer continue the test (to exhaustion). Allometrically scaled [45,46] peak VO2 (VO2_peak_^^0.67^), respiratory exchange ratio (RER), breaths per minute (BPM), maximum heartrate (HR_max_) and time to exhaustion were recorded using the breath-by-breath method powered by Vyaire’s SentrySuite software (Vyaire medical, Hoechberg, Germany). The following criteria had to be met for the measures to be accepted: (i) VO2 plateaued despite increased exercise intensity and (ii) RER > 1.0. Further methodological details are provided in the supplementary materials (S1: methodological details).

### 2.4. HRV Data Management

The IBI time series was retrieved from Garmin 920XT through Garmin connect by downloading the Garmin FIT file. The Garmin FIT file was thereafter imported to Kubios Standard HRV version 3.3 (Kuopio Oy, Finland) for further analysis. The analysis was conducted according to the recommendations [3,6] with a smooth priors detrending method and a sampling frequency of 500 Hz.

#### 2.4.1. Artifact Identification

To be able to obtain a clean short-term 5 min of IBI time series, the last 6 min of the recording was exported from Kubios to a text file for artifact identification. If the last 6 min of the recording had ≥5% artifact, the HRV data of the participant were disqualified from further analysis. Artifacts were first visually inspected [3], then the IBI time series was examined for artifacts using the Berntson et al. [43] artifact detection algorithm, which is based on a real estimation of the distribution of IBI differences from the individual participants’ IBI data. Then, percentile-based distribution indices were applied, and a removal of the artifact from the first and fourth quartile was carried out to calculate an estimate of the overall artifact-free standard deviation leading to a final calculation of the IBI threshold criterion for the difference between IBI to identify the artifact. This procedure was carried out automatically using ARTiiFACT tool version 2.13 (University of Würzburg, Würzburg, Germany) [47]. The artifact was identified and manually deleted from the IBI data series [43,47]. In their report, the task force [3] recommended reporting the number of data points edited and analyzed; therefore, Table 3 represents the artefact identification based on 6 min and the final number of data points analyzed based on 5 min.

#### 2.4.2. Measurements of HRV Indices

The cleaned IBI data were then imported to Kubios in order to calculate measures of HRV indices. From the time domain method, MeanRR (ms), SDNN (ms), RMSSD (ms) and pNN50 (%) were estimated. From the frequency domain method, HF (0.15–0.4 Hz) and LF (0.04–0.15 Hz) in absolute values of power (ms^2^) and normalized units (HF or LF/(HF+LF) × 100) were estimated. From the nonlinear (Poincaré plot) method, SD1 and SD2 were estimated [3,6,7]. To standardize and advance the field of study, source data and all calculations of HRV are provided (S1: Source data and all calculations of HRV).

### 2.5. Statistical Analysis

**Figure 1:**
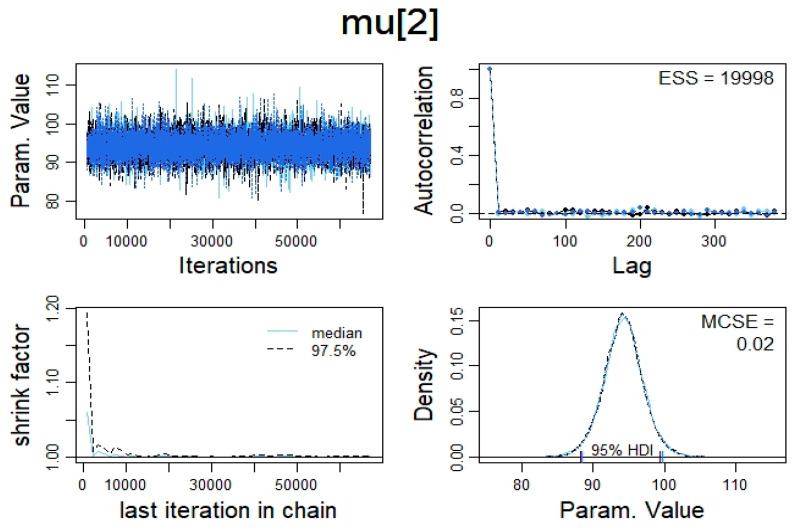
Example of representativeness and accuracy checks by examining the convergence of the Markov Chain Monte Carlo (MCMC) algorithm (the figure describes the convergence diagnostics for the variable MAP).

The data were prepared for analyses using Microsoft Excel for office 365 version 16 (Microsoft, Redmond, WA, USA). Thereafter, all statistical analyses were conducted using R package version 3.5.3 (R Core Team, Vienna, Austria) and RStudio version 1.2.5033 (RStudio Team, Boston, MA, USA). The Bayesian estimation of correlation (Bayesian reallocation of credibility across possibilities also known as the Bayes’ rule) was calculated using the Bayesian hierarchical model with Markov Chain Monte Carlo (MCMC) simulation as a part of the model, which is integrated in JAGS package version 4.3.0 (Martyn Plummer, international agency for research on cancer, Lyon, France). However, the Bayes’ rule dictates that the Bayesian estimated correlation has equal probability to fall at any point within the 95% HDI. Therefore, an estimated coefficient (*rho* = median) would not be enough evidence of association if the 95% HDI overlapped zero [48,49]. The R code (S1: The R code) provides the complete script, including the model specification and the graphics commands adapted from and adjusted after Kruschke [49]. The MCMC simulation was carried out by running 3 “chains”, 500 “adapt” steps, 500 steps to “burn-in” and 10th place as a number of “thin” with 20,000 samples to save for each variable in this study [49]. To apply the model [49,50,51], the data should be reasonably normally distributed and not be too kurtotic [49]. Therefore, the data were explored through a histogram plot, and the normality of distribution was tested using Shapiro–Wilk’s test, Skewness and Kurtosis (the *p* value for the Shapiro–Wilk’s test was set to *p* ≤ 0.05). For the data to be considered too kurtotic, the kurtosis should exceed ± 3 [52]. The descriptive statistics were reported as Bayesian estimated median ± 95% HDI and standard deviations (SD) ± 95% HDI of the estimated SD median for all the participants on all recorded variables in this study (Table 4). Furthermore, to be able to make an intuitive decision regarding the size of the Bayesian estimation of correlation, Cohen’s effect size for correlation was adapted, where a correlation of 0.1–0.29, 0.3–0.49 and >0.5 were classified as small, medium and large, respectively [53]. Due to the small sample size, the data produced by the MCMC simulation had to pass several criteria: Firstly, retrospective power analysis using “Posterior predictive checks (PPC)”, which is described in details in [49,54]. The code for the model check is embedded within the R code (S1: The R code). However, the PPC was qualitatively assessed visually by examining the model and the actual data collected. The figures (S1: The complete correlation figures) show that the superimposed ellipses from the model on the scatter plot of the data effectively contain the data giving a good indication that the model fits the data produced in this study. Secondly, the values of the MCMC chain had to meet the criteria of representativeness and accuracy by examining the convergence of the MCMC algorithm which was checked visually (trace plot and density plot) and numerically (potential scale factor, effective sample size and Monte Carlo standard error) using the approach described by [49] (Figure 1; S1: methodological details). Thirdly, the data produced, should be in line with the data reported in the literature regarding the norms of measuring (see Section 4.1.). Fourthly, the relationships between indices of HRV produced in this study should be comparable to the relationships between HRV indices reported in the literature, as it has been shown that this relationship is very stable and remains stable because the SNS and PNS change dynamically.

## 3. Results

### 3.1. Sample Characteristics

Examining the normality of distribution using Shapiro–Wilk’s test, Skewness and Kurtosis (Table 4), it was found that all variables were approximately normally distributed except for BPM. Therefore, BPM were log transformed and a second examination of normality indicated an approximately normally distributed BPM (Table 4). The data were found to not exceed the kurtosis threshold of ±3 [52]. Sample characteristics are presented in Table 4.

### 3.2. The Relationship within HRV Indices

The results from the estimated Bayesian correlation between time, frequency and nonlinear domains indicate a clear relationship between SDNN (ms) and LF ms^2^ (*rho* = 0.9) with a higher probability (95% HDI) that the true correlation falls between *rho* = 0.568 and *rho* = 0.982. Similar results can be observed between SDNN (ms) and SD2 (Table 5). Furthermore, a relationship with a high probability that the correlation is large between RMSSD (ms) with HF ms^2^ and SD1 was detected (Table 5). Similar results can be observed for the pNN50 (%) relationship with HF ms^2^ and SD1 (Table 5). The results further show a clear indication of a large correlation between SD1 and HF ms^2^ and between SD2 and LF ms^2^ (Table 5).

### 3.3. The Relationship between Measures of HRV Indices and Measurments of Blood Pressure Indices and Aerobic Capacity Performance

**Figure 2:**
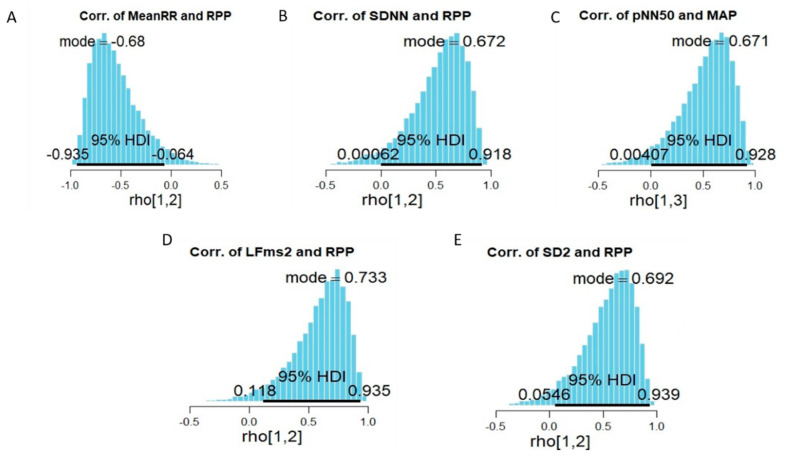
The probability of association between MeanRR and RPP (**A**), SDNN (ms) and RPP (**B**), pNN50 and MAP (**C**), LF (ms^2^) and RPP (**D**), SD2 and RPP (**E**).

The results from the correlation between measures of HRV and measurements of blood pressure and aerobic capacity performance (Table 6) indicate a high probability (95% HDI) of a relationship between MeanRR and RPP (Figure 2A), SDNN (ms) and RPP (Figure 2B), pNN50 and MAP (Figure 2C), LF (ms^2^) and RPP (Figure 2D) and finally SD2 and RPP (Figure 2E). The complete correlation figures are provided in the supplementary materials (S1. The complete correlation figures).

## 4. Discussion

The primary purpose of the present study was to use the Bayesian estimation of correlation to estimate the association between measures of HRV indices, aerobic performance and blood pressure indices using MCMC simulation in an attempt to explain the conflicting results reported in the literature by qualitatively comparing the reported results with the Bayesian estimated results. Hence, to assure the validity of the data presented in this study, a secondary aim was to compare the data produced to the reported norms and to investigate the estimation of the association within the HRV indices in comparison to those reported. The main finding in this study was that the simulation of 20,000 samples to produce the posterior possible association resulted in similar data that were within the reported norms. Furthermore, the association within the HRV indices is in line with what has been reported in the literature. Hence, the Bayesian estimation of correlation using MCMC simulation reproduced and supported the findings reported regarding the norms and within the HRV indices associations. Furthermore, the results of the Bayesian estimation showed that the association between HRV indices and aerobic performance parameters was unclear, indicated by the estimated *rho,* which has an equal chance to fall within the 95% HDI. Finally, a confirmed probability of association between HRV indices (PNS & SNS) and RPP was observed, and a trivial probability of association between pNN50 (%) and MAP at resting condition were also detected.

### 4.1. Measures of HRV Indices Compared to Norms

At the time of the task force report, there had been no comprehensive investigation regarding HRV indices, and the reported normal values were based on a few studies reporting both long and short term recordings [3]. The approximate values reported for the short-term recording (5 min) were for the LF ms^2^ = 1170 ± 416, HF ms^2^ = 975 ± 203, LF nu = 54 ± 4 and HF nu = 29 ± 3 [3]. It should be noted that the task force report did not account for age, sex, environment and recording condition (supine (rest), reactivation (during training) and rest (post activation)). Therefore, the normal values from the task force report could be considered incomparable to currently available studies. The most recent comprehensive HRV indices normal values were reported by Nunan et al. [40]. They investigated 3141 studies for suitability and concluded that from these, 44 studies were suitable (based on the task force guidelines) for further analysis with a total number of 21,438 participants. The study [40] accounted for age, sex, environment, recording condition and was based on short-term recordings (5 min). Furthermore, another investigation examining the Nunan et al. [40] study was conducted by Shaffer and Ginsberg [6]. It indicated that the participants reported on in the Nunan et al. [40] study had a minimum age of 40 years and were classified based on their sex. Therefore, the author of the present study feels that the Nunan et al. [40] study would be the most appropriate study for comparison with the results from this study. Based on the most credible value reported in Table 4, all participants of this study were within the normal range for MeanRR (873 ± 59 ms compared to the normal value range 785–1160 ms), SDNN (29 ± 10 ms compared to the normal value range 32–93 ms), RMSSD (24 ± 8 ms compared to the normal value range 19–75 ms), LF (ms^2^) (696 ± 552 ms^2^ compared to the normal value range 193–1009 ms^2^), LF (nu) (72 ± 18 nu compared to the normal value range 30–65 nu), HF (ms^2^) (179 ± 110 ms^2^ compared to the normal value range 83–3630 ms^2^) and HF (nu) (28 ± 19 nu compared to the normal value range 16–60 nu). Despite the fact that the measured variables in this study fall within the reported norms, the values were lower in this study compared to the norms data. This could be due to the participants in this study being older (53.9 ± 5.7 years) compared to those reported in Nunan et al. [40] (40 years old) and the different systems used for recording HRV. Research suggests that older people tend to have lower HRV indices values compared to younger people [2,6,7]. However, further research is needed to address the norms of HRV based on age, sex, environment and testing condition. For this reason, the source data are attached to this report (S1: Source data and all calculations of HRV). No further comparisons were possible because the normal values reported in Nunan et al. [40] did not report on all the estimated HRV metrics in this study.

### 4.2. The Association within HRV Indices

The most credible estimate of correlation value found in this study was first between SDNN (ms) (which reflects all factors contributing to HRV including SNS and PNS activities [7,8,10]) and LF ms^2^ band (*rho* = 0.9; 95% HDI = 0.568–0.982; Table 5). This relationship is in line with most of the reported studies [6,7,8,10]. However, HRV can be analyzed using the HF band (0.15–0.4 Hz) and the LF band (0.04–0.15 Hz). The LF band has been shown to reflect mainly the SNS activities in several studies [2,3,6,7,10,16,40,55,56]; however, due to the fact the this study measured HRV in resting condition, the relationship between SDNN (ms) and LF ms^2^ could be explained by baroreflex activity affecting the LF band compared to cardiac sympathetic innervation [2,3,6,7,10,16,40,55,56]. Hence, the fact that the PNS affects heart rhythms down to 0.05 Hz compared to the SNS, which has been reported to produce up to 0.1 Hz [6,16], explains the oscillations in the heart rhythms that can occur during resting vagal activities which cross over into the LF band [2,3,6,7,10,16,40,55,56], thus explaining the observed association in the present study. This explanation can be further confirmed by the strong association observed between the RMSSD and HF band compared to the LF band (Table 5) as RMSSD was suggested to be mainly influenced by PNS [6,10,57]. Similar results can be observed between SDNN and SD2 which represent the short- and long-term HRV [6,7] with the most credible correlation value of *rho* = 0.918 (95% HDI = 0.654–0.987; Table 5). Since SD2 reflects both SNS and PNS activities contributing to HRV (similar to LF), this association was expected and in line with what has been reported in the literature [6,7]. The similarity between SD2 and LF can be further demonstrated by the most credible relationship values found between the two in this study (*rho* = 0.893; 95% HDI = 0.575–0.98; Table 5).

Considering that the LF band does not cross the HF band [6,16], researchers concluded that the HF band reflects PSN/vagal activities [2,3,6,7,10,13,16,40,56], which is why it was later named the respiratory band as it relates to HRV indices related to breathing [6]. Therefore, it was expected that HF ms^2^ would have a strong relationship with RMSSD, as the RMSSD was classified as the primary time domain measure that reflects changes related to vagal activities affecting HRV [7,16,57]. This relationship was confirmed in the current study, and the results showed that the most credible correlation value between the two was *rho* = 0.9 (95% HDI = 0.567–0.982; Table 5). Furthermore, since pNN50 and RMSSD were reported to reflect short-term HRV changes and both reflect the PNS activities [6,57], it was expected that the pNN50 would add further affirmation to the results in this study when compared to the other studies. Indeed, the relationship found between pNN50 (%) and HF ms^2^ in this study confirms the association between RMSSD and HF ms^2^ (Table 5; *rho* = 0.896; 95% HDI = 0.574–0.984). Moreover, the SD1, which represents the fast beat to beat variability in IBI [7], has been reported to be the nonlinear domain metric that is identical to the time domain metric RMSSD [6]; the similarity between SD1 and HF, which correlates with baroreflex sensitivity [6,7], dictated the expectation that SD1 would correlate with RMSSD (*rho* = 0.92; 95% HDI = 0.665–0.987) and HF (*rho* = 0.895; 95% HDI = 0.582–0.982) which further confirms the relationship between the HF and RMSSD (Table 5).

It should be noted that no further confirmed probable associations were observed between time, frequency and nonlinear-domain variables in comparison to those reported by the task force [3] (Table 5). However, this might be due to the short-term measurement (5 min) used in this study compared to the 24 h measurements reported in the task force report, where the lack of association was caused by “both mathematical and physiological relationships [3]”. Furthermore, the used statistical analysis in this study, which is based on the Bayesian estimation of correlation [49], indicates that the highest probability (95% HDI) of the correlation values overlapped zero or near zero; therefore, those relationships were regarded as unclear. Finally, the reported short-term estimated relationships between time, frequency and nonlinear-domain variables reported in the literature [2,3,6,7,10,13,16,56] are comparable to the associations observed in this study.

### 4.3. The Relationship between Measures of HRV Indices and Both Measures of Blood Pressure Indices and Aerobic Capacity Parameters

The Bayesian estimation of correlation relocates credibility across possibilities [49,50,51]. Thus, the possibilities are reflected by the 95% HDI, and the probability of the estimated Bayesian correlation falling at any point within the 95% HDI is equal. Based on the Bayes’ rule, an estimated coefficient (*rho* = median) would not be enough evidence of association if the 95% HDI overlapped zero [48,49]. Therefore, the findings from this study did not provide enough evidence of the association between the measures of HRV indices and the parameters from the aerobic capacity test (i.e., VO2_Peak_, BPM, HR_max_ and Time to exhaustion; Table 6). Nevertheless, the results (Table 6 and Figure 2) indicate a possible association (regardless of the strength) between pNN50% and MAP, MeanRR (ms) and RPP, SDNN (ms) and RPP, LF (ms^2^) and RPP and SD2 and RPP (Figure 2).

Several studies have investigated changes in HRV indices as a result of performance adaptations in response to different exercise protocols. The results of these studies indeed confirm that changes in VO2_max_ are accompanied by changes in HRV indices [21,23,26,27,28,31]. The changes observed differed based on age and measuring condition (i.e., supine, standing) [28], duration of intervention [27,28,38], participants’ background [23,26] and measurement of time of day [39]. Nevertheless, the fact that research shows that the measures of resting HRV were not affected by exercise in the middle-aged group (50–59 years old) compared to the young group [26,28], that changes in HRV indices appear to flatten after 12 weeks of exercise [27] and that the maintenance of those changes could be achieved by exercising regularly [26], could in part explain *i)* the lack of evidence of association between measures of HRV indices and variables from the aerobic capacity test in this study and ii) the conflicting results reported in the literature. Another possible explanation is the newly reported results by Phoemsapthawee et al. [38], showing that changes in vagal-related HRV are related to individual ability to adapt to exercise. This was further confirmed by the multiple stepwise regression, which did not show a meaningful relationship between measures of HRV indices and changes in VO2_peak_ [38].

Studies investigated the relationship between measures of HRV indices and aerobic capacity directly and/or indirectly through establishing a prediction model to estimate aerobic capacity using the frequentist approach to data analysis [21,27,29,30,32,33,36,37,58]. Interestingly and regardless of whether the association was observed [30,33,36,37] or not [27,32], all the correlations reported in these studies falls within the 95% HDI reported in this study (Table 6). Several authors proposed explanations for the disparity in the results across those studies, which can be summarized by 3 major reasons: The first reason is the differences in the participants’ background, type of sport and age [26,29,32,33,35,58]. The results from these reports confirm that associations were detected in soccer players, distance runners, patients with chronic obstructive pulmonary disease and young people [26,29,32,33,58], but were not confirmed in middle-aged participants, untrained subjects, sprinters and throwers [26,29,32,36]. The variation related to the participants’ background was explained by the fact that the participants with already high values of vagal-related HRV indices in resting conditions tend to reach their anaerobic threshold at a higher exercise intensity compared to those who have lower values of vagal-related HRV indices [33]. The second reason is measurement position (i.e., supine, standing etc.). Studies have reported notably higher PNS activities in the seated rest compared to the standing position [21]. It was further investigated and reported that out of 30 possible correlations between HRV indices and aerobic capacity, only two from the supine position were associated with aerobic capacity compared to 15 from a standing position [32]. The third reason was the measuring time of day and condition (i.e., sleeping, early morning, evening); reported results showed that HRV indices measured in a resting supine position early in the morning (at wake up) did not differ between young and older participants. Nevertheless, the results indicate that there were differences in vagal-related HRV between age groups when measured during sleep, with the young group having higher values [28]. Furthermore, a review conducted by Vitale et al. [39] concluded that higher vagal-related HRV indices in the morning, compared to the evening, were observed and that they differed between individuals, which the authors used further to advise coaches and trainers to consider when planning the timing of exercise. Among all the studies above, no association was reported in a similar age group to the one in this study.

In this study, a possible inverse relationship was detected between MeanRR and RPP (Table 6, Figure 2). This was expected and was confirmed in the majority of published studies: simply stated, when the human body is exposed to a stressful demand (such as standing, performing daily activities and exercise), the SNS activities trigger the heart, and an increase in HR can be observed to meet the demands imposed on the body. This increase in HR is coupled with a decrease in time between the beat to beat interval [22]. This process is a good example of the combined actions of PNS and SNS in opposite directions, where the HR speeds up in response to a stimulus from the SNS and slows down in response to a stimulus from the PNS [59]. This can be further extended to explain the possible positive association observed between HRV indices reflecting mainly SNS activities (LF ms2 and SD2) and RPP (Table 6), indicating that an increase in SNS activities would cause higher RPP [10,16,59]. It is important to be reminded, as described earlier in this article, that the relationship between PNS and SNS is dynamic and that PNS activity could be associated with low, high or no SNS activities [6]. Therefore, the trivial (lower band of the 95% HDI at zero but not overlapping; Table 6) possible association between SDNN and RPP could be explained by the fact that measurements were carried out at resting condition, causing the PNS to be the dominant system. However, since RPP is the product of HR and systolic arterial pressure, and the systolic arterial pressure is only affected by the SNS [59], which has been reported to produce up to 0.1 Hz and crosses over to the LF band, it is expected that this trivial possibility will vanish with increased activity (see Section 4.2.). Finally, the relationship between HRV indices and MAP showed a potential but trivial (since the 95% HDI was almost at zero but not overlapping) possibility for association between pNN50 (a parameter primarily reflecting PNS activity) and MAP (Table 6). Nevertheless, while it was not expected to find a relationship at rest, it was expected that measures of PNS correlate positively with MAP [18]. This association could also be explained by the dynamic relationship between PNS and SNS explained earlier [6]. Furthermore, MAP involves both systolic and diastolic blood pressure [19,24], and the possible association could further be explained by the fact that the autonomic nervous system’s role in regulating MAP is to maintain it at the homeostasis level [18]. Hence, an elevation in MAP has been reported to cause a decrease in SNS activities and an increase in PSN activities [6,18,59].

In line with other studies, this study is not without limitations. Due to the difficulties involved in conducting such studies, the sample size was small, but still in line with the recommendation from the task force [3] to be able to establish norms through meta-analysis studies. The small sample size was compensated for by using the within-subjects experimental design as advised [4,5,9], the simulation of data using MCMC producing a simulated sample [49], the retrospective power analysis based on PPC and the assessment of representativeness and accuracy by examining the convergence of the MCMC algorithm. Furthermore, the number of participants in this study is in line with the majority of studies examining HRV in the field [12,15,28,31,35,60]. Participants’ in this study were tested during resting supine position only, which can also be viewed as a limitation; however, due to the measurement equipment used in this study, the measurements under other conditions (reactivation (during training) and rest (post-activation)) would have produced unreliable results. For those reasons, and due to the fact that this is the first study within sport sciences that uses the Bayesian estimation of correlation on MCMC simulated data using the 95% HDI, the author has attached all the necessary information for replicability (supplementary materials) in order to contribute to future advancements in the field.

## 5. Conclusions

The normal values reported in the literature were based on several studies which can be seen as a stable reference to evaluate HRV values. The relationships within the HRV indices can also be seen as stable relationships that are hard to change due to the dynamics between PNS and SNS activities. Hence, the Bayesian estimation of correlation using MCMC simulated data produced similar HRV values to the norms reported in the literature, and the associations found within HRV indices (time, frequency and nonlinear) were also in line with what has been reported in the literature. Nevertheless, while the measures in this study fell within the reported norms, the values were lower, which may have been caused by the Bayesian hierarchical model having a higher precision in calculating the most credible values of the Mean compared to the frequentist Mean value. However, this argument is not completely valid unless more studies are carried out. Therefore, it would be helpful if the reported studies could publish their data sources for other researchers to able to assess the data using the Bayesian hierarchical model and compare the results with what has been reported. Alternatively, the lower values could also have been caused by different recording conditions (supine, reactivation, rest), recording systems, time of day (morning, evening, sleep etc.) and age of participants, which would clearly give different results. Hence, it is advised to use the same condition and recording system when the repeated measure is intended. Furthermore, it could be noted that PNS and SNS activities are dynamic and depend on the individual’s baseline physiological and psychologically abilities and the ability to adapt to stressors, suggesting that the disparity in the association between the measures of HRV indices and aerobic capacity could be caused by a different dynamic between the two systems among individuals. Hence, it is expected that two individuals with the same aerobic capacity would have different PNS and SNS activities dynamics. Furthermore, the fact that PNS activity could be associated with low, high or no SNS activities and that it is individually based could explain the disparity in the results reported in the literature. Therefore, further investigation of the dynamics between PNS and SNS in different participants who share the same physiological abilities is strongly advised. Hence, the results from this investigation are valid only for the participants in this study and at the time of participation. Finally, the use of the Bayesian estimation of correlation with 95% HDI on MCMC simulated data is a new technique for data analysis in sport science and seems to provide a more robust approach to allocating credibility through a meaningful mathematical model. However, the 95% HDI found in this study, accompanied by the theoretical explanations regarding the dynamics between PNS and SNS in relation to different recording conditions (supine, reactivation, rest), recording systems, time of day (morning, evening, sleep etc.) and age of participants, suggests that the association between measures of HRV indices and aerobic performance parameters has yet to be explicated.

## Figures and Tables

**Table 1:** The three heart rate variability (HRV) domains.

**Time Domain**		
SDNN	Standard deviation of all Normal–Normal intervals in a time series	SDNN indicate total variability [3,7,8]
RMSSD	The root mean square of successive differences	RMSSD and pNN50 (%) reflects vagal tone/PNS activities [4,7,10]
pNN50 (%))	Percent of successive intervals with a difference greater that 50 ms compared to previous interval	
**Frequency Domain**		
HF	High-frequency band (i.e., 0.15 to 0.4 Hz)	HF reflects vagal tone/PNS activities [4,8,10]
LF	Low-frequency (LF) band (i.e., 0.04 and 0.15 Hz)	LF reflects baroreceptor activity at rest (vagal influenced) and SNS activities during stress [3,4,8,10]
VLF	Very-low-frequency (VLF) band (i.e., 0.0033 to 0.04 Hz)	VLF and ULF reflect long-term thermo- and hormonal regulation mechanisms [3,10]
ULF	Ultra-low-frequency (ULF) band (i.e., <0.0033 Hz)	
**Poincaré Plot**		
SD1	Standard descriptor 1	SD1 reflects fast IBI variability which is a reflection of PNS activities to the heart [4,7]
SD2	Standard descriptor 2	SD2 reflects the long-term IBI variability, which represents both SNS and PSN activities [4,7]

Note: Both VLF and ULF physiological explanations are less defined and, therefore, are not usually included in the HRV reporting. PNS = parasympathetic nervous system; SNS = sympathetic nervous system; IBI = inter-beat interval.

**Table 2:** A brief review of the literature showing the disparity in the reported results.

Study (Date)	Focus	Findings
De Meersman [26] (year 1993)	Compared different age groups on both VO2_max_ and HRV (assessed by the percent change in mean HR).	(a) Results showed that the participants with higher VO2_max_ have a higher value of HRV.
Melanson and Freedson [27] (year 2001)	Effect of a 12-week endurance training on resting heart rate variability in sedentary adult males.	(a) Increase in VO2_peak_, no change in resting HR, increase in RMSSD and pNN50 after 12 weeks of training, but not during, compared to the baseline. (b) No effect on LF (nu) and only the training group had elevated HF power compared to the baseline. (c) No marked correlations were observed between HRV indices and VO2_peak_ except for HRV indices after 16 weeks of training.
Catai et al. [28] (year 2002)	Effects of aerobic exercise training on heart rate variability during wakefulness and sleep and cardiorespiratory responses of young and middle-aged healthy men.	(a) Exercise training increased VO2_peak_ in all participants with no marked changes observed in HRV indices.
Kouidi et al. [29] (year 2002)	Effect of athletic training on time domain HRV indices.	(a) A relationship between aerobic capacity and time domain HRV indices on athletes training in the long-distance group but not on athletes from the sprint track group, the field weight events group and the control group. They concluded that HRV modulation depends on the exercise training pattern.
Marocolo et al. [30] (year 2007)	Effect of aerobic training program on the electrical remodeling of heart high-frequency components.	(a) Positive correlation between HRV indices and VO2_max_; however, the root mean squared voltage of total (a variable from the signal-averaged ECG) was the only independent predictor of VO2_max_.
Schmitt et al. [31] (year 2008)	Altitude, heart rate variability and aerobic capacities.	(a) No relationship between changes in HRV and VO2_max._ (b) The increase in VO2 and power at the respiratory compensation point was accompanied by decreased PNS activities. (c) The changes in HRV parameters and the changes in VO2 and power at the respiratory compensation point had a statistically significant relationship.
Grant et al. [32] (year 2009)	Relationship between exercise capacity and heart rate variability.	(a) Relationship between VO2_max_ and HRV indices showed different results in relation to measuring position. (b) LF (nu) and LF/HF correlated with VO2_max_ when HRV was recorded in the supine resting position. (c) RMSSD, pNN50, SD1, LF (nu), HF (ms^2^) and LF/HF were correlated with VO2_max_ when HRV was recorded in a standing position.
Leite et al. [33] (year 2015)	Correlation between heart rate variability indexes and aerobic physiological variables.	(a) A moderate but statistically significant relationship between HRV and aerobic capacity in patients with chronic obstructive pulmonary disease.
Flatt and Esco [34] (year 2016)	Evaluating individual raining adaptation with smartphone-derived heart rate variability in a collegiate female soccer team.	(a) A statistically significant large relationship between change in RMSSD (expressed as a coefficient of variation) and Yo-Yo IR1 test performance. (b) No statistically significant relationship was observed between mean change of RMSSD and Yo-Yo IR1 test performance.
Flatt et al. [35] (year 2017)	Individual heart rate variability responses to preseason training in high level female soccer players.	(a) Inverse, very large, relationship between mean weekly changes in RMSSD and changes in daily and weekly training loads. (b) Positive and large relationship between mean weekly changes in RMSSD and both fatigue and soreness.
Materko [36] (year 2018)	Stratification of the level of aerobic fitness based on heart rate variability parameters in adult males at rest.	(a) The group with higher VO2_max_ also had higher HRV. (b) Only pNN50 was among the HRV indices, together with Cardiac-Deceleration Rate, that was able to predict VO2_max_
Materko et al. [37] (year 2018)	Maximum oxygen uptake prediction model based on heart rate variability parameters.	(a) Small to moderate relationship between measures of HRV indices (i.e., RMSSD, pNN50, HF and LF/HF) and VO2_max_ (b) Only pNN50 was among the HRV indices, together with mean HR and Cardiac-Deceleration Rate, that was able to predict VO2_max_
Phoemsapthawee et al. [38] (year 2019)	Clarifying the casual link between body composition, aerobic fitness and the alterations in cardiac autonomic modulation after a 12-week exercise training.	(a) Improvement in VO2_peak_ after the training period in the exercise group. (b) Increased PNS indices and reduced SNS indices at rest. (c) Statistically significant relationship between changes in VO2_peak_ and HRV indices. (d) Only SD1/SD2 ratio gave a statistically significant explanation for the changes in VO2_peak_

VO2_peak_, VO2_max_ = highest rate of oxygen consumption measured during incremental exercise.

**Table 3:** Identification and deletion of the artefact based on the last 6 recorded minutes and the number of the final data points used in HRV analysis.

Based on the Last 6 min				Based on 5 min
Participant Number	Total Data Point	Artifact	Percentage	Total Data Point Analyzed
P. 1	392	17	4.3	333
P. 2	445	0	0.0	369
P. 3	434	0	0.0	363
P. 4	396	1	0.3	329
P. 5	457	4	0.9	383
P. 6	391	2	0.5	324
P. 7	420	0	0.0	349
P. 8	369	2	0.5	309

**Table 4:** Bayesian estimated mean and SD ± HDI, Shapiro–Wilk’s test, Skewness and Kurtosis of the raw data.

Variable	Mean ± (95% HDI)	SD ± (95% HDI)	Shapiro–Wilk’s Test (Sig.)	Skewness	Kurtosis
Mean HR (bpm)	69.3 (65.5–73.1)	4.93 (3.11–8.78)	0.870	0.17	−1.14
Min HR (bpm)	64.5 (61.3–67.5)	3.91 (2.46–7.06)	0.811	−0.11	−1.08
Max HR (bpm)	76 (71.2–81)	6.13 (4.01–11.3)	0.979	0.00	0.92
MeanRR (ms)	873 (824–924)	58.7 (40.1–111)	0.896	0.06	−1.09
SDNN (ms)	28.9 (20.4–37.1)	9.93 (6.59–18.5)	0.911	0.24	−0.22
RMSSD (ms)	23.8 (17.4–30.8)	8.17 (5.23–14.8)	0.185	0.09	−2.18
pNN50 (%)	5.34 (1.11–9.51)	5.22 (3.39–9.48)	0.132	0.49	−1.64
HF (ms^2^)	179 (92.2–273)	110 (73.2–205)	0.677	0.56	−0.73
LF (ms^2^)	696 (244–1150)	552 (354–1010)	0.255	1.36	2.34
HF (n.u)	28.2 (13.1–42.9)	18.6 (11.6–33.1)	0.158	1.22	1.65
LF (n.u)	71.7 (57.5–87.2)	17.7 (11.8–33.5)	0.170	−1.19	1.57
SD1	17 (12.3–21.8)	5.68 (3.84–10.7)	0.181	0.09	−2.19
SD2	36.6 (25.6–48)	13.5 (8.81–25.1)	0.734	0.29	0.35
RPP (mmHg/min)	9330 (8210–10,400)	1320 (893–2480)	0.333	0.83	1.05
MAP (mmHg)	94.4 (88.6–99.9)	6.98 (4.48–12.5)	0.090	1.52	2.30
VO2peak^−67^	123 (105–140)	21.2 (13.6–38.6)	0.167	0.37	−1.75
RER	1.13 (1.06–1.19)	0.08 (0.05–0.15)	0.920	−0.00	−1.02
BPM	41.5 (34.4–48.4)	8.49 (5.7–15.9)	0.047 * (Nor. 0.097)	1.26 (Nor. 1.10)	0.28 (Nor. −0.18)
HRmax (bpm)	170 (165–176)	6.29 (4.17–11.7)	0.791	−0.01	1.01
Time to exhaustion (s)	842 (718–956)	140 (91.3–261)	0.192	−0.89	0.19

MeanRR = average time for successive heart beats; SDNN = standard deviation of all Normal–Normal intervals; RMSSD = root mean square of SDs between successive N–N intervals; pNN50 = percent of successive intervals with a difference greater that 50 ms compared to previous interval; HF = high frequency; LF = low frequency; SD1 = standard descriptor 1; SD2 = standard descriptor 2; RPP = rate-pressure product; MAP = mean arterial blood pressure; RER = respiratory exchange ratio; BPM = breaths per minute; HRmax = maximum heartrate during exercise; Nor. = calculation after transforming the data that were not observed to follow normality; HDI = high density interval; * = *p* ≤ 0.05.

**Table 5:** Correlation matrix between HRV measures from time domain, frequency domain and nonlinear domain.

Variable		HF (ms^2^)	LF (ms^2^)	HF (n.u)	LF (n.u)	SD1	SD2
MeanRR (ms)	*rho*	0.357	−0.465	0.324	−0.332	0.231	−0.393
	Upper 95% HDI	0.793	0.284	0.802	0.394	0.799	0.32
	Lower 95% HDI	−0.378	−0.85	−0.392	−0.803	−0.397	−0.824
SDNN (ms)	*rho*	0.651	0.9	−0.702	0.653	0.765	0.918
	Upper 95% HDI	0.923	0.982	−0.074	0.932	0.954	0.987
	Lower 95% HDI	0.041	0.568	−0.937	0.065	0.235	0.654
RMSSD (ms)	*rho*	0.9	0.716	−0.177	0.269	0.92	0.721
	Upper 95% HDI	0.982	0.943	0.464	0.767	0.987	0.947
	Lower 95% HDI	0.567	0.093	−0.752	−0.464	0.665	0.141
pNN50 (%)	*rho*	0.896	0.749	−0.307	0.282	0.907	0.785
	Upper 95% HDI	0.984	0.946	0.42	0.778	0.98	0.953
	Lower 95% HDI	0.574	0.146	−0.781	−0.416	0.588	0.23
SD1	*rho*	0.895	0.691	−0.146	0.179		
	Upper 95% HDI	0.982	0.94	0.483	0.724		
	Lower 95% HDI	0.582	0.1	−0.742	−0.507		
SD2	*rho*	0.605	0.893	−0.731	0.724		
	Upper 95% HDI	0.901	0.98	−0.137	0.942		
	Lower 95% HDI	−0.083	0.575	−0.94	0.135		

**Table 6:** Correlation matrix between HRV measures from time domain, frequency domain and nonlinear domain.

Variable		RPP	MAP	PeakVO2	BPM	HRmax	Time
MeanRR (ms)	*rho*	−0.68	0.134	0.44	0.050	−0.144	0.464
	Upper 95% HDI	−0.064	0.676	0.858	0.655	0.563	0.853
	Lower 95% HDI	−0.935	−0.564	−0.251	−0.601	−0.682	−0.249
SDNN (ms)	*rho*	0.672	0.578	0.132	−0.195	0.488	−0.398
	Upper 95% HDI	0.918	0.89	0.724	0.423	0.863	0.344
	Lower 95% HDI	0.001	−0.12	−0.521	−0.778	−0.216	−0.819
RMSSD (ms)	*rho*	0.366	0.605	0.419	−0.209	0.668	−0.221
	Upper 95% HDI	0.83	0.912	0.843	0.488	0.91	0.415
	Lower 95% HDI	−0.319	−0.058	−0.278	−0.737	−0.044	−0.788
pNN50 (%)	*rho*	0.376	0.671	0.423	−0.237	0.629	−0.37
	Upper 95% HDI	0.827	0.928	0.833	0.432	0.907	0.36
	Lower 95% HDI	−0.31	0.004	−0.323	−0.765	−0.056	−0.805
HF (ms^2^)	*rho*	0.303	0.639	0.461	−0.153	0.626	−0.295
	Upper 95% HDI	0.782	0.924	0.858	0.481	0.917	0.412
	Lower 95% HDI	−0.426	−0.017	−0.257	−0.74	−0.06	−0.787
LF (ms^2^)	*rho*	0.733	0.629	0.031	−0.227	0.41	−0.323
	Upper 95% HDI	0.935	0.904	0.624	0.434	0.819	0.371
	Lower 95% HDI	0.118	−0.095	−0.624	−0.772	−0.328	−0.81
HF (n.u)	*rho*	−0.346	−0.177	−0.157	−0.061	−0.089	−0.023
	Upper 95% HDI	0.388	0.477	0.496	0.641	0.558	0.613
	Lower 95% HDI	−0.8	−0.757	−0.729	−0.615	−0.702	−0.647
LF (n.u)	*rho*	0.345	0.246	0.16	0.011	0.064	−0.009
	Upper 95% HDI	0.804	0.748	0.719	0.633	0.683	0.616
	Lower 95% HDI	−0.379	−0.471	−0.528	−0.624	−0.559	−0.638
SD1	*rho*	0.362	0.599	0.473	−0.217	0.652	−0.303
	Upper 95% HDI	0.825	0.909	0.856	0.497	0.916	0.426
	Lower 95% HDI	−0.344	−0.064	−0.28	−0.747	−0.055	−0.782
SD2	*rho*	0.692	0.551	0.078	−0.242	0.481	−0.403
	Upper 95% HDI	0.939	0.906	0.672	0.42	0.852	0.326
	Lower 95% HDI	0.055	−0.134	−0.578	−0.785	−0.265	−0.824

## Supplementary Materials

The following are available online at https://www.mdpi.com/1660-4601/17/18/6750/s1; S1: Methodological details; S1: Source data and all calculations of HRV; S1: The R code; S1: The complete correlation figures.
