# Supplementary material for: Bayesian Estimation of Correlation between Measures of Blood Pressure Indices, Aerobic Capacity and Resting Heart Rate Variability Using Markov Chain Monte Carlo Simulation and 95% High Density Interval in Female School Teachers
Source: Int J Environ Res Public Health. 2020 Sep 16;17(18):6750. doi: 10.3390/ijerph17186750 (PMC7558932; doi:10.3390/ijerph17186750)
Supplement: Supplementary file 1 [file ijerph-17-06750-s001.pdf]

S1: Methodological details

Supplementary methodological details

Blood pressure (BP)

Blood pressure was administrated manually using Reister stethoscope (model: Anestophon) and sphygmomanometer (model: Big Ben; Rudolf Riester GmbH, Jungingen, Germany). All blood pressure instruments were tested and approved by the British and Irish Hypertension Society (BHS) Validation Service (Society, 2020). Blood pressure was assessed according to the recommendations described in Kallioinen et al. (Kallioinen, Hill, Horswill, Ward, & Watson, 2017) by first asking the participants to set on a height adjustable mechanical chair with resting their back to the chair and feet on the floor for ~5 minutes, the left arm was rested on a table in front of the participant and the seat height was adjusted so the arm is in a level with the heart. Then, the deflated BP cuff was wrapped around the upper arm with the artery marker over the brachial artery pulse followed by placing the earpieces of the stethoscope between the arm and the cuff locating the auditory canals. Then, controllably the cuff was inflated to about 20 to 30 mmHg above the estimated systolic pressure (estimated by observing the sphygmomanometer pressure gauge), then the sphygmomanometer valve was slowly and partially opened to release pressure and when the first sharp heartbeat was heard, the BP mmHg was registered (systolic BP) and when the sound disappear BP mmHg was registered (diastolic pressures), HR was noted together with BP. Baseline rate-pressure product (RPP) (RPP = heart rate x systolic arterial pressure) and mean arterial blood pressure (MAP = [(Systolic blood pressure − Diastolic blood pressure) ÷ 3] + Diastolic blood pressure) were then estimated (Ansari et al., 2012; Swank & Sharp, 2016).

HRV data acquisition

As briefly presented in the introduction, several studies investigated different heartrate monitors to record HRV time series validity and accuracy in several conditions such as supine (rest), reactivation (during training) and rest (post activation) (Cassirame, Vanhaesebrouck, Chevrolat, & Mourot, 2017; Hernando, Garatachea, Almeida, Casajus, & Bailon, 2016; Kingsley, Lewis, & Marson, 2005; Nunan et al., 2009). The reported results from these investigations revealed a high accuracy in supine position whereas during activity and post activity the measures remained questionable. In this study, Garmin 920XT (Garmin Ltd, Olathe, Kansas, USA) was used to record HRV in a resting supine position (rest) pre-aerobic capacity test. The Garmin 920XT has been reported to have high HRV accuracy compared to ECG measurements when recording at rest at a supine position (Cassirame et al., 2017). Prior to measuring HRV, Garmin HRM-Tri was placed around center of the chest and below the level of breasts. Garmin HRM-Tri uses ANT+ technology to transfer heart rate measures to the Garmin 920XT using a 2.4 GHz ANT wireless communication protocol. To measure Short-Term HRV (5 min), the Garmin 920XT was preprogramed to record 10 min, the 10 min recording was chosen to ensure that participants had enough time in the acclimatization to the recording environment (Laborde, Mosley, & Thayer, 2017), and to record enough data points to be able achieve a clean 5 minutes of HRV recording (Berntson et al., 1997; Berntson, Quigley, Jang, & Boysen, 1990; Task_Force, 1996). To ensure comparability of results across studies and laboratories, the short-term (5 min) recording was adopted in line with the task force recommendations (Task_Force, 1996). The participants were asked to lay down on a gym mat in a supine position, then the recording started.

Aerobic capacity test

After completing the HRV recording and prior to aerobic capacity test start, mask size was chosen to insure headspace correction and the Vyntus CPX gas analyzer (Model: versatile JAEGER; Vyaire medical, Hoechberg, Germany) was calibrated using the fully automated 2-point gas calibration of the O2/CO2, through a special Twin Tube sample line combined with a fresh air flush system (Vyaire, 2016). Then participants were tested on a motorized treadmill (Ergo ELG 55) that is connected to a programmable external WOODWAY User-System version 2.0 (Woodway GmbH, Weilam Rhein, Germany). The modified Bruce continuous incremental test protocol which is believed to be the most suitable to test the participants in this study (Gibson, Wagner, & Heyward, 2019) was preprogrammed into the WOODWAY User-System. The testing protocol was similar to the standard Bruce protocol except for the first 2 stages, where stage 1 was at speed 2.7 km/h and 0% incline and stage 2 was at 5% incline with no change in speed. The test continues until participant could no longer continue the test (to exhaustion). Allometrically scaled (Gibson et al., 2019; Nevill, Ramsbottom, & Williams, 1992) peak VO2 ($VO2_{peak}^{\wedge 0.67}$), respiratory exchange ratio (RER), breaths per minute (BPM), maximum heartrate (HR_{max}) and time to exhaustion were recorded using the breath-by-breath method powered by Vyaire's SentrySuite software (Vyaire medical, Hoechberg, Germany). The following criteria had to be met for the measures to be accepted; (i) VO2 plateaued despite increased exercise intensity and (ii) RER > 1.0.

Check the convergence of the MCMC algorithm

The convergence of the MCMC algorithm was checked using the approach described by (J. K. Kruschke, 2015, p. 178). Hence:

a) Firstly, the MCMC algorithm was checked for ***representativeness*** by visual inspection of the chain trajectory (trace plot; Figure 1, upper left, labeled as iterations). Since in this study the MCMC simulation was carried out by running 3 “chains”, those chains should overlap each other to be accepted as representative (figure 1 down here is from the diagnostic function provided with the source code (# Convergence diagnostics) that was supplied together with the article for the variable *MAP*). As can be seen from this figure (trace plot; Figure 1, upper left, labeled as iterations), the 3 chains overlap smoothly and mix well. The visual representativeness was further inspected using the density plot (Figure 1, lower right, labeled as Parm. Value) where it can further be seen that the 3 chains overlap smoothly.
b) Secondly, the convergence of the MCMC algorithm was checked numerically by inspecting the “potential scale factor” (Figure 1, lower left, labeled as shrink factor). The shrink factor ideal value is 1, and it can be clearly seen from the figure that it converges at almost 1, which indicate a good convergence.
c) The MCMC accuracy was assessed using the *“Effective sample size”* (ESS), the ESS needs to be relatively large and the recommended ESS is ≥ 10000 (J. K. Kruschke, 2015). This can be seen from the upper right plot (Figure 1, upper right, labeled as autocorrelation). Furthermore, the accuracy can further be assessed using the Monte Carlo standard error (MCSE), which can be seen in figure 1 lower right ((Figure 1, lower right, labeled as density). MCSE indicate the estimated SD of the sample mean in the chain, the closer the number to zero,

the more stable the chains. This can clearly be observed in figure 1 (MCSE = 0.02).

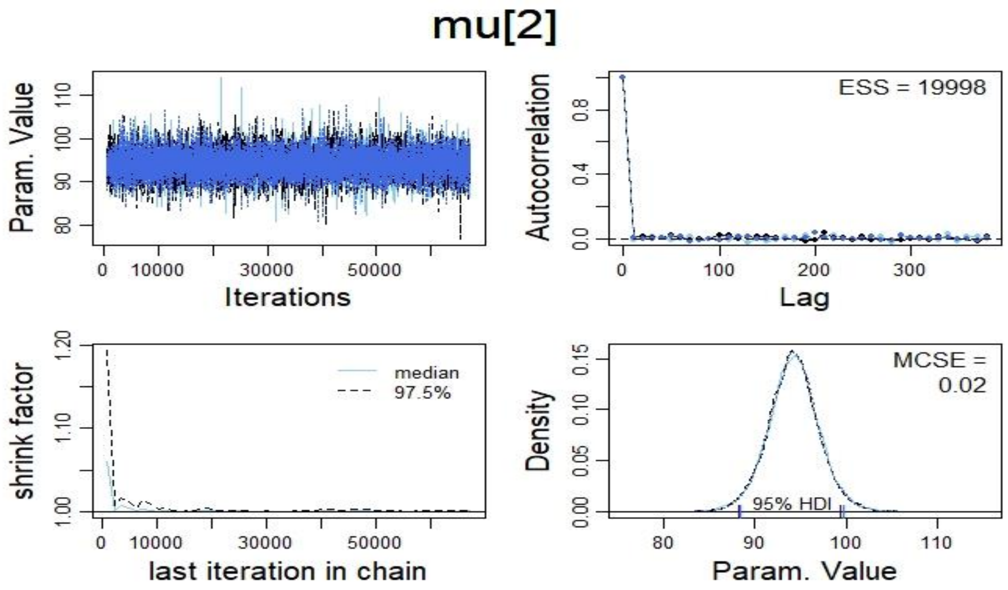

Figure 1: illustration of the MCMC diagnostic check.

Model checking

The model used in this study was assessed for its reasonability as a good description of the data. The method used in this assessment was based on the *"Posterior predictive checks (PPC)"* which is described in details in (John K. Kruschke, 2013). The code for the model check is embedded with the R code provided with the supplementary files. However, the PPC was qualitatively assessed visually by examining the model and the actual data collected (plots were provided in the supplementary results file). The figure shows that the superimposed ellipses from the model on the scatter plot of the data contain the data, this is a good indication of the model fit for the data produced in this study.

References:

Ansari, M., Javadi, H., Pourbehi, M., Mogharrabi, M., Rayzan, M., Semnani, S., . . . Assadi, M. (2012). The association of rate pressure product (RPP) and myocardial perfusion imaging (MPI) findings: a preliminary study. *Perfusion, 27*(3), 207-213. doi:10.1177/0267659112436631

Berntson, G. G., Bigger, J. T., Jr., Eckberg, D. L., Grossman, P., Kaufmann, P. G., Malik, M., . . . van der Molen, M. W. (1997). Heart rate variability: origins, methods, and interpretive caveats. *Psychophysiology, 34*(6), 623-648. doi:10.1111/j.1469-8986.1997.tb02140.x

Berntson, G. G., Quigley, K. S., Jang, J. F., & Boysen, S. T. (1990). An approach to artifact identification: application to heart period data. *Psychophysiology, 27*(5), 586-598. doi:10.1111/j.1469-8986.1990.tb01982.x

Cassirame, J., Vanhaesebrouck, R., Chevrolat, S., & Mourot, L. (2017). Accuracy of the Garmin 920 XT HRM to perform HRV analysis. *Australas Phys Eng Sci Med, 40*(4), 831-839. doi:10.1007/s13246-017-0593-8

Gibson, A. L., Wagner, D. R., & Heyward, V. H. (2019). *Advanced fitness assessment and exercise prescription*. Champaign, IL: Human Kinetics.

Hernando, D., Garatachea, N., Almeida, R., Casajus, J. A., & Bailon, R. (2016). Validation of Heart Rate Monitor Polar RS800 for Heart Rate Variability Analysis During Exercise. *J Strength Cond Res, 32*(3), 716-725. doi:10.1519/jsc.0000000000001662

Kallioinen, N., Hill, A., Horswill, M. S., Ward, H. E., & Watson, M. O. (2017). Sources of inaccuracy in the measurement of adult patients' resting blood pressure in clinical settings: a systematic review. *Journal of hypertension, 35*(3), 421-441. doi:10.1097/HJH.0000000000001197

Kingsley, M., Lewis, M. J., & Marson, R. E. (2005). Comparison of Polar 810s and an ambulatory ECG system for RR interval measurement during progressive exercise. *Int J Sports Med, 26*(1), 39-44. doi:10.1055/s-2004-817878

Kruschke, J. K. (2013). Posterior predictive checks can and should be Bayesian: Comment on Gelman and Shalizi, 'Philosophy and the practice of Bayesian statistics'. *British Journal of Mathematical and Statistical Psychology, 66*(1), 45-56. doi:10.1111/j.2044-8317.2012.02063.x

Kruschke, J. K. (2015). *Doing Bayesian data analysis. A tutorial with R, JAGS, and Stan* (Second Edition ed.). Oxford, UK: Elsevier.

Laborde, S., Mosley, E., & Thayer, J. F. (2017). Heart Rate Variability and Cardiac Vagal Tone in Psychophysiological Research – Recommendations for Experiment Planning, Data Analysis, and Data Reporting. *Frontiers in psychology, 8*(213). doi:10.3389/fpsyg.2017.00213

Nevill, A. M., Ramsbottom, R., & Williams, C. (1992). Scaling physiological measurements for individuals of different body size. *Eur J Appl Physiol Occup Physiol, 65*(2), 110-117. doi:10.1007/bf00705066

Nunan, D., Donovan, G., Jakovljevic, D. G., Hodges, L. D., Sandercock, G. R., & Brodie, D. A. (2009). Validity and reliability of short-term heart-rate variability from the Polar S810. *Med Sci Sports Exerc, 41*(1), 243-250. doi:10.1249/MSS.0b013e318184a4b1

Society, B. a. I. H. (2020). Validated BP Monitors for For Specialist Use. Retrieved from https://bihsoc.org/bp-monitors/for-specialist-use/

Swank, A., & Sharp, C. (2016). Adaptations to aerobic endurance training programs. In G. G. Haff & N. T. Triplett (Eds.), *Essentials of strength training and conditioning* (4th ed., pp. 115-). Champaign, IL: Human Kinetics.

Task_Force. (1996). Heart rate variability: standards of measurement, physiological interpretation and clinical use. Task Force of the European Society of Cardiology and the North American Society of Pacing and Electrophysiology. *Circulation, 93*(5), 1043-1065.

Vyaire, m. (2016). Vyntus CPX Powered by SentrySuite. Retrieved from https://www.vyaire.com/Documents/international/brochures/respiratory-care/cardiopulmonary/RC_Vyntus-CPX_BR_EN.pdf

S1: Source data and all calculations of HRV

HRV ANALYSIS RESULTS - 02-Mar-2020 12:27:23
Kubios HRV Standard
3.3.1
released in August 2019

Analyzed by: Participant 1 - -

File name: C:\Users
Measurement date: xx/xx/xx xx:xx:xx
File type: asciiRR
Channel label: RR data
Data length: 00:05:38 (h:min:s)
Measurement rate: -

Parameters
Number of samples: 1
Detrending method: Smoothn priors (lambda: 500)
Min/Max HR as average of: 5 beats
Threshold for NNxx/pNNxx: 50 ms
Frequency bands
VLF: 0 - 0.04 Hz
LF: 0.04 - 0.15 Hz
HF: 0.15 - 0.4 Hz
Interpolation rate: 4 Hz
Points in frequency-domain: 300 points/Hz
FFT spectrum options
Window width: 300 s
Window overlap: 50 %
AR spectrum options
AR model order: 16
Use factorization: No
Apply detrending for nonlinear analysis: 1
Entropy embedding dimension: 2

Entropy	tolerance: 0.2 x SD
DFA	short-term fluctuations: 4-12 beats
DFA	long-term fluctuations: 13-64 beats

RR Interval Samples Selected for Analysis

	Sample 1
Sample limit:	00:00:38-00:05:38
Sample Analysis Type: Single sample	
Artifact correction: none	
Artifacts (%):	-

RESULTS FOR A SINGLE SAMPLE

Results Overview

PNS index:	-0.3548
SNS index:	0.5577
Stress index:	12.6396

Time-Domain Results

Statistical parameters

Mean RR (m	899.5225
STD RR (ms):	35.2227
Mean HR (be	66.7021
STD HR (beat	2.6701
Min HR (beat	61.8022
Max HR (bea	77.0218
RMSSD (ms):	32.2284
NNxx (beats)	36
pNNxx (%):	10.8434
SDANN (ms):	
SDNN index (	

Geometric parameters

RR tri index:	10.40625	
TINN (ms):	157	
Frequency-Do	FFT spectrum	AR spectrum
Peak frequencies		
VLF (Hz):	0.033333	0.04
LF (Hz):	0.073333	0.093333
HF (Hz):	0.156667	0.15
Absolute powers		
VLF (ms^2):	108.0818	115.9231
LF (ms^2):	839.0429	697.9255
HF (ms^2):	299.0992	301.7114
VLF (log):	4.6829	4.7529
LF (log):	6.7323	6.5481
HF (log):	5.7008	5.7095
Relative powers		
VLF (%):	8.6665	10.3824
LF (%):	67.278	62.5081
HF (%):	23.983	27.0221
Normalized powers		
LF (n.u.):	73.6619	69.7498
HF (n.u.):	26.2587	30.1527
Total power (	1247.1279	1116.5361
LF/HF ratio:	2.8052	2.3132

Nonlinear Results	
Poincare plot	
SD1 (ms):	22.823347
SD2 (ms):	44.344726
SD2/SD1 rati	1.942955
Approximate	1.1857
Sample entro	1.9202

Detrended fluctuation analysis (DFA)

alpha 1:	1.1454
alpha 2:	0.3564

RR INTERVAL DATA and SPECTRUM ESTIMATES

SAMPLE 1

RR Data		FFT spectrum		AR Spectrum				
Time (s)	RR interval (s)	Frequency (Hz)	PSD (ms^2/Hz)	Frequency (Hz)	PSD (ms^2/Hz)	VLF comp. (ms^2/Hz)	LF comp. (ms^2/Hz)	HF comp. (ms^2/Hz)
38.5	0.921	0	28.503	0	1329.1593			
39.393	0.893	0.003	16.2641	0.003	2663.8467			
40.29	0.897	0.007	2.5436	0.007	2680.5112			
41.133	0.843	0.01	16.6174	0.01	2708.5545			
41.968	0.835	0.013	46.9449	0.013	2748.3871			
42.874	0.906	0.017	340.8888	0.017	2800.5962			
43.784	0.91	0.02	1957.3616	0.02	2865.9598			
44.629	0.845	0.023	1121.7469	0.023	2945.4631			
45.483	0.854	0.027	340.969	0.027	3040.3194			
46.357	0.874	0.03	3291.8901	0.03	3151.9943			
47.238	0.881	0.033	14429.7203	0.033	3282.2323			
48.083	0.845	0.037	8849.8151	0.037	3433.0835			
48.967	0.884	0.04	4198.8783	0.04	3606.9283			
49.852	0.885	0.043	10869.1051	0.043	3806.4926			
50.715	0.863	0.047	19525.547	0.047	4034.8454			
51.625	0.91	0.05	7072.2577	0.05	4295.3608			
52.534	0.909	0.053	3493.4701	0.053	4591.6216			
53.439	0.905	0.057	7188.2433	0.057	4927.2255			
54.315	0.876	0.06	282.1667	0.06	5305.4392			
55.214	0.899	0.063	1305.1476	0.063	5728.6272			
56.101	0.887	0.067	292.3228	0.067	6197.3666			
57.001	0.9	0.07	8034.7238	0.07	6709.1606			
57.878	0.877	0.073	29871.0466	0.073	7256.7209			

58.781	0.903	0.077	12928.1225	0.077	7825.9314
59.72	0.939	0.08	4474.9044	0.08	8393.9016
60.661	0.941	0.083	18098.4867	0.083	8927.9474
61.565	0.904	0.087	2742.4949	0.087	9386.7457
62.51	0.945	0.09	17067.882	0.09	9724.8782
63.466	0.956	0.093	8739.616	0.093	9900.9897
64.413	0.947	0.097	3626.4338	0.097	9887.7584
65.351	0.938	0.1	4382.6485	0.1	9679.888
66.242	0.891	0.103	3513.7887	0.103	9296.2464
67.158	0.916	0.107	2578.7742	0.107	8774.899
68.044	0.886	0.11	1189.2584	0.11	8163.4348
68.918	0.874	0.113	1421.4594	0.113	7508.8755
69.746	0.828	0.117	1051.2983	0.117	6850.5598
70.562	0.816	0.12	2604.4421	0.12	6217.0646
71.513	0.951	0.123	11137.1891	0.123	5626.3594
72.427	0.914	0.127	15542.9143	0.127	5087.7187
73.336	0.909	0.13	13802.537	0.13	4604.1485
74.305	0.969	0.133	5672.4701	0.133	4174.599
75.189	0.884	0.137	2357.3614	0.137	3795.6804
76.029	0.84	0.14	9789.4059	0.14	3462.8597
76.891	0.862	0.143	10984.0633	0.143	3171.2296
77.759	0.868	0.147	6674.0082	0.147	2915.9647
78.651	0.892	0.15	3294.0751	0.15	2692.5669
79.562	0.911	0.153	620.9711	0.153	2496.9774
80.479	0.917	0.157	6298.7265	0.157	2325.6095
81.382	0.903	0.16	1788.8988	0.16	2175.3362
82.28	0.898	0.163	1496.1392	0.163	2043.4556
83.176	0.896	0.167	2992.2032	0.167	1927.646
84.083	0.907	0.17	2189.1605	0.17	1825.9201
84.94	0.857	0.173	410.1957	0.173	1736.58
85.825	0.885	0.177	43.5298	0.177	1658.1771
86.73	0.905	0.18	265.4484	0.18	1589.4756
87.638	0.908	0.183	456.9337	0.183	1529.422

88.554	0.916	0.187	940.568	0.187	1477.1174
89.468	0.914	0.19	1495.671	0.19	1431.7953
90.385	0.917	0.193	4128.906	0.193	1392.8011
91.289	0.904	0.197	5714.4116	0.197	1359.5762
92.175	0.886	0.2	952.1339	0.2	1331.643
93.003	0.828	0.203	65.8761	0.203	1308.5928
93.817	0.814	0.207	308.2901	0.207	1290.0748
94.653	0.836	0.21	310.9641	0.21	1275.7862
95.488	0.835	0.213	684.6228	0.213	1265.4638
96.332	0.844	0.217	395.3169	0.217	1258.8749
97.207	0.875	0.22	99.0163	0.22	1255.81
98.112	0.905	0.223	2798.4624	0.223	1256.0739
99.032	0.92	0.227	2419.8483	0.227	1259.4776
99.935	0.903	0.23	1202.3928	0.23	1265.8294
100.871	0.936	0.233	1498.3144	0.233	1274.925
101.823	0.952	0.237	455.7631	0.237	1286.5373
102.781	0.958	0.24	275.2653	0.24	1300.4051
103.734	0.953	0.243	3219.2804	0.243	1316.2213
104.688	0.954	0.247	5603.1816	0.247	1333.6212
105.629	0.941	0.25	2741.5745	0.25	1352.171
106.557	0.928	0.253	367.1118	0.253	1371.3584
107.471	0.914	0.257	2061.5336	0.257	1390.5864
108.327	0.856	0.26	305.9334	0.26	1409.1723
109.217	0.89	0.263	471.2739	0.263	1426.3547
110.149	0.932	0.267	341.1021	0.267	1441.3091
111.098	0.949	0.27	1654.105	0.27	1453.1759
112.065	0.967	0.273	1938.3893	0.273	1461.0989
113.03	0.965	0.277	454.4671	0.277	1464.2751
113.965	0.935	0.28	724.6504	0.28	1462.0104
114.965	1	0.283	3649.1713	0.283	1453.7759
115.852	0.887	0.287	2550.0879	0.287	1439.2577
116.721	0.869	0.29	101.1545	0.29	1418.3921
117.648	0.927	0.293	642.2769	0.293	1391.3789

118.559	0.911	0.297	929.4418	0.297	1358.6715
119.482	0.923	0.3	734.4188	0.3	1320.9426
120.366	0.884	0.303	3987.4059	0.303	1279.032
121.294	0.928	0.307	1203.0428	0.307	1233.8829
122.245	0.951	0.31	9.9272	0.31	1186.4766
123.168	0.923	0.313	373.7091	0.313	1137.773
124.134	0.966	0.317	91.341	0.317	1088.6615
125.127	0.993	0.32	641.7935	0.32	1039.928
126.099	0.972	0.323	715.4575	0.323	992.2351
126.978	0.879	0.327	42.1241	0.327	946.1158
127.854	0.876	0.33	114.8388	0.33	901.9773
128.763	0.909	0.333	513.1901	0.333	860.1115
129.628	0.865	0.337	1093.213	0.337	820.7101
130.451	0.823	0.34	157.1769	0.34	783.8808
131.26	0.809	0.343	77.7168	0.343	749.6637
132.115	0.855	0.347	20.4784	0.347	718.0464
132.995	0.88	0.35	1225.2387	0.35	688.9776
133.901	0.906	0.353	3151.045	0.353	662.3782
134.819	0.918	0.357	1066.4742	0.357	638.1503
135.745	0.926	0.36	43.8704	0.36	616.1846
136.674	0.929	0.363	436.097	0.363	596.3658
137.612	0.938	0.367	549.9931	0.367	578.5766
138.535	0.923	0.37	394.302	0.37	562.7005
139.39	0.855	0.373	258.8678	0.373	548.6232
140.291	0.901	0.377	156.8565	0.377	536.234
141.23	0.939	0.38	199.4543	0.38	525.4253
142.187	0.957	0.383	85.0731	0.383	516.0926
143.134	0.947	0.387	361.3972	0.387	508.1333
144.057	0.923	0.39	1326.3689	0.39	501.4454
144.974	0.917	0.393	18.2559	0.393	495.9256
145.876	0.902	0.397	1198.0325	0.397	491.467
146.735	0.859	0.4	424.5262	0.4	487.9564
147.575	0.84	0.403	17.1266	0.403	485.2719

148.458	0.883	0.407	313.0059	0.407	483.2796
149.352	0.894	0.41	463.6231	0.41	481.8305
150.268	0.916	0.413	695.2462	0.413	480.7582
151.154	0.886	0.417	1064.5878	0.417	479.8763
151.979	0.825	0.42	888.7841	0.42	478.978
152.807	0.828	0.423	332.4149	0.423	477.8363
153.647	0.84	0.427	80.5367	0.427	476.2072
154.553	0.906	0.43	238.3005	0.43	473.8357
155.485	0.932	0.433	171.5523	0.433	470.4648
156.388	0.903	0.437	42.9562	0.437	465.8485
157.345	0.957	0.44	139.7445	0.44	459.7667
158.293	0.948	0.443	2.0266	0.443	452.0429
159.18	0.887	0.447	762.1823	0.447	442.5603
160.091	0.911	0.45	1109.1885	0.45	431.2762
160.981	0.89	0.453	189.4213	0.453	418.2302
161.86	0.879	0.457	281.6486	0.457	403.5461
162.79	0.93	0.46	625.882	0.46	387.4247
163.73	0.94	0.463	883.5204	0.463	370.1297
164.686	0.956	0.467	159.4436	0.467	351.9675
165.657	0.971	0.47	244.6253	0.47	333.2647
166.604	0.947	0.473	406.0726	0.473	314.3455
167.5	0.896	0.477	2.8257	0.477	295.5125
168.419	0.919	0.48	229.7384	0.48	277.0322
169.324	0.905	0.483	146.3778	0.483	259.1262
170.276	0.952	0.487	568.7836	0.487	241.9674
171.233	0.957	0.49	497.4842	0.49	225.6808
172.183	0.95	0.493	262.2601	0.493	210.3477
173.11	0.927	0.497	170.3357	0.497	196.0113
174.011	0.901	0.5	27.5469	0.5	182.6837
174.906	0.895				
175.745	0.839				
176.591	0.846				
177.473	0.882				

178.343	0.87
179.268	0.925
180.203	0.935
181.153	0.95
182.102	0.949
182.969	0.867
183.857	0.888
184.742	0.885
185.619	0.877
186.453	0.834
187.248	0.795
188.066	0.818
188.876	0.81
189.722	0.846
190.588	0.866
191.462	0.874
192.314	0.852
193.14	0.826
194.014	0.874
194.897	0.883
195.787	0.89
196.674	0.887
197.554	0.88
198.438	0.884
199.323	0.885
200.209	0.886
201.043	0.834
201.927	0.884
202.811	0.884
203.715	0.904
204.573	0.858
205.507	0.934
206.459	0.952

207.414	0.955
208.309	0.895
209.177	0.868
210.075	0.898
210.988	0.913
211.901	0.913
212.825	0.924
213.744	0.919
214.625	0.881
215.455	0.83
216.272	0.817
217.137	0.865
217.997	0.86
218.877	0.88
219.825	0.948
220.807	0.982
221.752	0.945
222.717	0.965
223.662	0.945
224.591	0.929
225.539	0.948
226.534	0.995
227.519	0.985
228.477	0.958
229.367	0.89
230.298	0.931
231.284	0.986
232.249	0.965
233.224	0.975
234.193	0.969
235.137	0.944
236.052	0.915
236.934	0.882

237.816	0.882
238.674	0.858
239.517	0.843
240.367	0.85
241.188	0.821
242.008	0.82
242.879	0.871
243.775	0.896
244.71	0.935
245.614	0.904
246.568	0.954
247.51	0.942
248.446	0.936
249.341	0.895
250.282	0.941
251.251	0.969
252.179	0.928
253.174	0.995
254.09	0.916
255.035	0.945
256.002	0.967
256.974	0.972
257.9	0.926
258.854	0.954
259.781	0.927
260.685	0.904
261.623	0.938
262.571	0.948
263.51	0.939
264.428	0.918
265.361	0.933
266.323	0.962
267.261	0.938

268.185	0.924
269.093	0.908
270	0.907
270.905	0.905
271.752	0.847
272.565	0.813
273.401	0.836
274.21	0.809
275.057	0.847
275.946	0.889
276.872	0.926
277.795	0.923
278.701	0.906
279.581	0.88
280.415	0.834
281.231	0.816
282.005	0.774
282.811	0.806
283.63	0.819
284.432	0.802
285.241	0.809
286.084	0.843
286.95	0.866
287.83	0.88
288.704	0.874
289.589	0.885
290.458	0.869
291.341	0.883
292.194	0.853
293.103	0.909
294.033	0.93
294.89	0.857
295.729	0.839

296.628	0.899
297.549	0.921
298.428	0.879
299.332	0.904
300.231	0.899
301.146	0.915
302.093	0.947
303.049	0.956
304	0.951
304.916	0.916
305.788	0.872
306.693	0.905
307.613	0.92
308.546	0.933
309.448	0.902
310.392	0.944
311.315	0.923
312.271	0.956
313.205	0.934
314.102	0.897
315.024	0.922
315.952	0.928
316.88	0.928
317.846	0.966
318.838	0.992
319.831	0.993
320.821	0.99
321.783	0.962
322.723	0.94
323.653	0.93
324.567	0.914
325.463	0.896
326.337	0.874

327.194	0.857
328.048	0.854
328.926	0.878
329.84	0.914
330.753	0.913
331.604	0.851
332.414	0.81
333.193	0.779
333.959	0.766
334.753	0.794
335.549	0.796
336.341	0.792
337.12	0.779

HRV ANALYSIS RESULTS - 02-Mar-2020 12:30:27
Kubios HRV Standard
3.3.1
released in August 2019

Analyzed by: Participant 2 - -

File name: C:\Users
Measurement date: xx/xx/xx xx:xx:xx
File type: asciiRR
Channel label: RR data
Data length: 00:06:00 (h:min:s)
Measurement rate: -

Parameters
 Number of samples: 1
 Detrending method: Smoothn priors (lambda: 500)
 Min/Max HR as average of: 5 beats
 Threshold for NNxx/pNNxx: 50 ms
 Frequency bands
  VLF: 0 - 0.04 Hz
  LF: 0.04 - 0.15 Hz
  HF: 0.15 - 0.4 Hz
 Interpolation rate: 4 Hz
 Points in frequency-domain: 300 points/Hz
 FFT spectrum options
  Window width: 300 s
  Window overlap: 50 %
 AR spectrum options
  AR model order: 16
  Use factorization: No
 Apply detrending for nonlinear analysis: 1
 Entropy embedding dimension: 2

Entropy	tolerance: 0.2 x SD
DFA	short-term fluctuations: 4-12 beats
DFA	long-term fluctuations: 13-64 beats

RR Interval Samples Selected for Analysis

	Sample 1
Sample limit:	00:01:00-00:06:00
Sample Analysis Type:	Single sample
Artifact correction:	none
Artifacts (%):	-

RESULTS FOR A SINGLE SAMPLE

Results Overview

PNS index:	-1.3898
SNS index:	1.609
Stress index:	15.5258

Time-Domain Results

Statistical parameters

Mean RR (m	813.4797
STD RR (ms):	26.4195
Mean HR (be	73.7572
STD HR (beat	2.3603
Min HR (beat	67.9409
Max HR (bea	79.0222
RMSSD (ms):	13.7896
NNxx (beats)	3
pNNxx (%):	0.8152
SDANN (ms):	
SDNN index (	

Geometric parameters

RR tri index:	7.235294	
TINN (ms):	125	
Frequency-Do	FFT spectrum	AR spectrum
Peak frequencies		
VLF (Hz):	0.036667	0.04
LF (Hz):	0.063333	0.073333
HF (Hz):	0.23	0.15
Absolute powers		
VLF (ms^2):	62.3754	98.0494
LF (ms^2):	376.4238	555.7639
HF (ms^2):	33.9356	43.9873
VLF (log):	4.1332	4.5855
LF (log):	5.9307	6.3203
HF (log):	3.5245	3.7839
Relative powers		
VLF (%):	13.1941	14.0498
LF (%):	79.6238	79.6368
HF (%):	7.1783	6.3031
Normalized powers		
LF (n.u.):	91.7262	92.6546
HF (n.u.):	8.2694	7.3334
Total power (	472.753	697.8728
LF/HF ratio:	11.0923	12.6346
Nonlinear Results		
Poincare plot		
SD1 (ms):	9.764019	
SD2 (ms):	36.109568	
SD2/SD1 rati	3.698228	
Approximate	1.0742	
Sample entro	1.2799	

Detrended fluctuation analysis (DFA)

alpha 1:	1.517
alpha 2:	0.4049

RR INTERVAL DATA and SPECTRUM ESTIMATES

SAMPLE 1

RR Data		FFT spectrum		AR Spectrum				
Time (s)	RR interval (s)	Frequency (Hz)	PSD (ms^2/Hz)	Frequency (Hz)	PSD (ms^2/Hz)	VLF comp. (ms^2/Hz)	LF comp. (ms^2/Hz)	HF comp. (ms^2/Hz)
60.152	0.803	0	68.9197	0	1008.0066			
61	0.848	0.003	33.0535	0.003	2023.8206			
61.857	0.857	0.007	0.2514	0.007	2047.4802			
62.711	0.854	0.01	11.8205	0.01	2087.7186			
63.547	0.836	0.013	47.3065	0.013	2145.7956			
64.4	0.853	0.017	150.2724	0.017	2223.583			
65.247	0.847	0.02	1066.4555	0.02	2323.6839			
66.072	0.825	0.023	2838.6641	0.023	2449.6051			
66.892	0.82	0.027	2261.0309	0.027	2605.9964			
67.694	0.802	0.03	3348.245	0.03	2798.9803			
68.51	0.816	0.033	4293.9383	0.033	3036.5966			
69.331	0.821	0.037	4474.9492	0.037	3329.392			
70.152	0.821	0.04	399.8962	0.04	3691.1731			
70.96	0.808	0.043	6244.9419	0.043	4139.8989			
71.766	0.806	0.047	8312.0339	0.047	4698.5612			
72.575	0.809	0.05	9561.9887	0.05	5395.5595			
73.377	0.802	0.053	945.4548	0.053	6263.289			
74.178	0.801	0.057	13726.6923	0.057	7331.9717			
74.976	0.798	0.06	4709.7438	0.06	8612.8797			
75.773	0.797	0.063	13860.8546	0.063	10062.5596			
76.576	0.803	0.067	7090.2967	0.067	11526.33			
77.384	0.808	0.07	4994.8258	0.07	12695.5157			
78.199	0.815	0.073	5631.3949	0.073	13175.0289			

79.013	0.814	0.077	254.7634	0.077	12720.5125
79.839	0.826	0.08	624.4354	0.08	11452.4184
80.651	0.812	0.083	243.0105	0.083	9767.3243
81.472	0.821	0.087	585.9251	0.087	8056.5379
82.277	0.805	0.09	1456.9427	0.09	6544.4248
83.082	0.805	0.093	5921.9096	0.093	5301.5472
83.869	0.787	0.097	5366.4798	0.097	4315.2698
84.653	0.784	0.1	628.2831	0.1	3543.3479
85.454	0.801	0.103	2387.5979	0.103	2940.3361
86.27	0.816	0.107	8300.804	0.107	2467.1322
87.072	0.802	0.11	3471.1947	0.11	2092.8777
87.864	0.792	0.113	1916.6128	0.113	1794.111
88.648	0.784	0.117	2711.8088	0.117	1553.2547
89.416	0.768	0.12	562.5022	0.12	1357.1816
90.169	0.753	0.123	5.1051	0.123	1196.0587
90.913	0.744	0.127	882.2388	0.127	1062.4742
91.654	0.741	0.13	971.4073	0.13	950.7961
92.406	0.752	0.133	1153.3896	0.133	856.7072
93.181	0.775	0.137	0.3816	0.137	776.8673
93.997	0.816	0.14	184.4962	0.14	708.6688
94.872	0.875	0.143	267.1694	0.143	650.0579
95.763	0.891	0.147	41.7049	0.147	599.4027
96.652	0.889	0.15	43.0963	0.15	555.396
97.509	0.857	0.153	9.4062	0.153	516.9825
98.346	0.837	0.157	29.2381	0.157	483.3033
99.168	0.822	0.16	46.6361	0.16	453.655
99.983	0.815	0.163	99.8474	0.163	427.457
100.767	0.784	0.167	67.2649	0.167	404.2269
101.526	0.759	0.17	469.6169	0.17	383.5614
102.303	0.777	0.173	977.4832	0.173	365.1211
103.072	0.769	0.177	296.8472	0.177	348.6185
103.827	0.755	0.18	24.4024	0.18	333.8087
104.568	0.741	0.183	153.6057	0.183	320.4813

105.323	0.755	0.187	71.2919	0.187	308.4547
106.093	0.77	0.19	24.9651	0.19	297.5711
106.864	0.771	0.193	161.9184	0.193	287.6921
107.645	0.781	0.197	127.6787	0.197	278.6957
108.434	0.789	0.2	101.6047	0.2	270.4734
109.231	0.797	0.203	35.8359	0.203	262.9279
110.057	0.826	0.207	219.4889	0.207	255.9715
110.914	0.857	0.21	280.3848	0.21	249.5242
111.802	0.888	0.213	173.2029	0.213	243.5129
112.708	0.906	0.217	205.0898	0.217	237.8704
113.621	0.913	0.22	57.9899	0.22	232.5341
114.513	0.892	0.223	20.8628	0.223	227.4462
115.379	0.866	0.227	226.0639	0.227	222.5532
116.226	0.847	0.23	1202.3699	0.23	217.8053
117.051	0.825	0.233	733.7074	0.233	213.1568
117.855	0.804	0.237	227.021	0.237	208.5661
118.647	0.792	0.24	135.5403	0.24	203.9959
119.441	0.794	0.243	3.0496	0.243	199.4134
120.226	0.785	0.247	42.6145	0.247	194.791
121.005	0.779	0.25	176.2827	0.25	190.106
121.778	0.773	0.253	96.9953	0.253	185.3412
122.553	0.775	0.257	143.8339	0.257	180.4854
123.325	0.772	0.26	86.2694	0.26	175.5329
124.105	0.78	0.263	145.7005	0.263	170.4837
124.897	0.792	0.267	327.0843	0.267	165.3435
125.697	0.8	0.27	351.5206	0.27	160.1227
126.515	0.818	0.273	348.2583	0.273	154.8362
127.34	0.825	0.277	310.2709	0.277	149.5026
128.165	0.825	0.28	370.6548	0.28	144.1435
128.99	0.825	0.283	163.5676	0.283	138.7822
129.81	0.82	0.287	4.193	0.287	133.4431
130.624	0.814	0.29	33.2191	0.29	128.1509
131.429	0.805	0.293	28.1908	0.293	122.9295

132.231	0.802	0.297	22.9434	0.297	117.8016
133.032	0.801	0.3	3.7342	0.3	112.7879
133.828	0.796	0.303	43.4979	0.303	107.9069
134.624	0.796	0.307	67.217	0.307	103.1744
135.416	0.792	0.31	152.0125	0.31	98.6035
136.203	0.787	0.313	39.164	0.313	94.2048
136.997	0.794	0.317	13.366	0.317	89.9859
137.795	0.798	0.32	49.8959	0.32	85.952
138.584	0.789	0.323	76.4199	0.323	82.1062
139.382	0.798	0.327	121.5731	0.327	78.4491
140.187	0.805	0.33	172.4697	0.33	74.98
141.013	0.826	0.333	113.8309	0.333	71.6964
141.855	0.842	0.337	68.4865	0.337	68.5945
142.685	0.83	0.34	33.0331	0.34	65.6695
143.5	0.815	0.343	12.5087	0.343	62.9161
144.301	0.801	0.347	26.6065	0.347	60.328
145.096	0.795	0.35	9.8531	0.35	57.8987
145.881	0.785	0.353	0.2125	0.353	55.6216
146.676	0.795	0.357	56.1626	0.357	53.4896
147.469	0.793	0.36	13.1102	0.36	51.4959
148.281	0.812	0.363	2.8934	0.363	49.6336
149.106	0.825	0.367	3.9405	0.367	47.8962
149.94	0.834	0.37	6.9172	0.37	46.2771
150.772	0.832	0.373	14.1452	0.373	44.7701
151.606	0.834	0.377	20.9231	0.377	43.3692
152.419	0.813	0.38	21.2045	0.38	42.0687
153.224	0.805	0.383	87.3369	0.383	40.8634
154.022	0.798	0.387	42.9905	0.387	39.7481
154.807	0.785	0.39	5.8559	0.39	38.7181
155.604	0.797	0.393	22.7628	0.393	37.7689
156.392	0.788	0.397	7.0062	0.397	36.8965
157.187	0.795	0.4	9.4616	0.4	36.0968
157.986	0.799	0.403	7.6375	0.403	35.3663

158.807	0.821	0.407	44.9499	0.407	34.7017
159.632	0.825	0.41	114.1405	0.41	34.0999
160.451	0.819	0.413	32.5289	0.413	33.5579
161.271	0.82	0.417	25.7944	0.417	33.0731
162.084	0.813	0.42	25.1551	0.42	32.6431
162.897	0.813	0.423	54.7392	0.423	32.2653
163.724	0.827	0.427	66.3417	0.427	31.9378
164.549	0.825	0.43	91.714	0.43	31.6583
165.368	0.819	0.433	84.0069	0.433	31.4249
166.182	0.814	0.437	10.1607	0.437	31.2356
166.995	0.813	0.44	3.718	0.44	31.0885
167.799	0.804	0.443	23.1992	0.443	30.9818
168.605	0.806	0.447	13.6788	0.447	30.9134
169.418	0.813	0.45	28.97	0.45	30.8813
170.231	0.813	0.453	37.8509	0.453	30.8834
171.034	0.803	0.457	27.0094	0.457	30.9173
171.833	0.799	0.46	43.2252	0.46	30.9805
172.616	0.783	0.463	52.1616	0.463	31.0701
173.395	0.779	0.467	8.5541	0.467	31.1832
174.161	0.766	0.47	0.3141	0.47	31.3162
174.92	0.759	0.473	7.3346	0.473	31.4652
175.674	0.754	0.477	24.8272	0.477	31.626
176.443	0.769	0.48	23.3128	0.48	31.7936
177.215	0.772	0.483	7.3475	0.483	31.963
177.986	0.771	0.487	53.5425	0.487	32.1283
178.756	0.77	0.49	9.9687	0.49	32.2833
179.529	0.773	0.493	0.4022	0.493	32.4216
180.306	0.777	0.497	10.5364	0.497	32.5362
181.094	0.788	0.5	14.2065	0.5	32.6202
181.874	0.78				
182.642	0.768				
183.403	0.761				
184.145	0.742				

184.9	0.755
185.66	0.76
186.437	0.777
187.239	0.802
188.029	0.79
188.855	0.826
189.675	0.82
190.517	0.842
191.327	0.81
192.14	0.813
192.946	0.806
193.76	0.814
194.583	0.823
195.402	0.819
196.215	0.813
197.028	0.813
197.852	0.824
198.685	0.833
199.51	0.825
200.325	0.815
201.129	0.804
201.936	0.807
202.741	0.805
203.557	0.816
204.387	0.83
205.228	0.841
206.078	0.85
206.921	0.843
207.773	0.852
208.628	0.855
209.469	0.841
210.312	0.843
211.167	0.855

212.022	0.855
212.882	0.86
213.732	0.85
214.582	0.85
215.447	0.865
216.307	0.86
217.163	0.856
218.009	0.846
218.851	0.842
219.702	0.851
220.548	0.846
221.381	0.833
222.213	0.832
223.065	0.852
223.911	0.846
224.743	0.832
225.556	0.813
226.354	0.798
227.145	0.791
227.921	0.776
228.689	0.768
229.45	0.761
230.223	0.773
230.999	0.776
231.8	0.801
232.62	0.82
233.452	0.832
234.307	0.855
235.157	0.85
235.985	0.828
236.804	0.819
237.618	0.814
238.424	0.806

239.21	0.786
239.984	0.774
240.758	0.774
241.585	0.827
242.382	0.797
243.171	0.789
243.946	0.775
244.733	0.787
245.532	0.799
246.337	0.805
247.147	0.81
247.962	0.815
248.78	0.818
249.613	0.833
250.485	0.872
251.366	0.881
252.264	0.898
253.155	0.891
254.052	0.897
254.931	0.879
255.798	0.867
256.648	0.85
257.477	0.829
258.277	0.8
259.057	0.78
259.828	0.771
260.583	0.755
261.343	0.76
262.11	0.767
262.889	0.779
263.664	0.775
264.443	0.779
265.23	0.787

266.022	0.792
266.821	0.799
267.62	0.799
268.408	0.788
269.194	0.786
269.965	0.771
270.759	0.794
271.541	0.782
272.329	0.788
273.132	0.803
273.957	0.825
274.78	0.823
275.61	0.83
276.448	0.838
277.278	0.83
278.102	0.824
278.925	0.823
279.74	0.815
280.567	0.827
281.402	0.835
282.235	0.833
283.085	0.85
283.945	0.86
284.811	0.866
285.691	0.88
286.565	0.874
287.424	0.859
288.261	0.837
289.102	0.841
289.936	0.834
290.775	0.839
291.607	0.832
292.452	0.845

293.305	0.853
294.15	0.845
294.981	0.831
295.808	0.827
296.631	0.823
297.46	0.829
298.288	0.828
299.121	0.833
299.956	0.835
300.791	0.835
301.634	0.843
302.472	0.838
303.294	0.822
304.106	0.812
304.898	0.792
305.692	0.794
306.49	0.798
307.296	0.806
308.109	0.813
308.916	0.807
309.718	0.802
310.513	0.795
311.301	0.788
312.083	0.782
312.858	0.775
313.629	0.771
314.403	0.774
315.183	0.78
315.993	0.81
316.817	0.824
317.675	0.858
318.54	0.865
319.42	0.88

320.305	0.885
321.187	0.882
322.055	0.868
322.907	0.852
323.755	0.848
324.586	0.831
325.412	0.826
326.219	0.807
327.004	0.785
327.779	0.775
328.576	0.797
329.389	0.813
330.202	0.813
331.008	0.806
331.822	0.814
332.639	0.817
333.456	0.817
334.27	0.814
335.08	0.81
335.897	0.817
336.725	0.828
337.562	0.837
338.393	0.831
339.215	0.822
340.023	0.808
340.858	0.835
341.667	0.809
342.45	0.783
343.234	0.784
344.007	0.773
344.781	0.774
345.559	0.778
346.336	0.777

347.133	0.797
347.921	0.788
348.71	0.789
349.505	0.795
350.323	0.818
351.189	0.866
352.085	0.896
352.981	0.896
353.864	0.883
354.695	0.831
355.501	0.806
356.32	0.819
357.125	0.805
357.911	0.786
358.712	0.801
359.523	0.811

HRV ANALYSIS RESULTS - 02-Mar-2020 12:32:19
Kubios HRV Standard
3.3.1
released in August 2019

Analyzed by: Participant 3 - -

File name: C:\Users
Measurement date: xx/xx/xx xx:xx:xx
File type: asciiRR
Channel label: RR data
Data length: 00:06:01 (h:min:s)
Measurement rate: -

Parameters
 Number of samples: 1
 Detrending method: Smoothn priors (lambda: 500)
 Min/Max HR as average of: 5 beats
 Threshold for NNxx/pNNxx: 50 ms
 Frequency bands
  VLF: 0 - 0.04 Hz
  LF: 0.04 - 0.15 Hz
  HF: 0.15 - 0.4 Hz
 Interpolation rate: 4 Hz
 Points in frequency-domain: 300 points/Hz
 FFT spectrum options
  Window width: 300 s
  Window overlap: 50 %
 AR spectrum options
  AR model order: 16
  Use factorization: No
 Apply detrending for nonlinear analysis: 1
 Entropy embedding dimension: 2

Entropy	tolerance: 0.2 x SD
DFA	short-term fluctuations: 4-12 beats
DFA	long-term fluctuations: 13-64 beats

RR Interval Samples Selected for Analysis

	Sample 1
Sample limit:	00:01:01-00:06:01
Sample Analysis Type:	Single sample
Artifact correction:	none
Artifacts (%):	-

RESULTS FOR A SINGLE SAMPLE

Results Overview

PNS index:	-1.0575
SNS index:	3.0121
Stress index:	26.5407

Time-Domain Results

Statistical parameters

Mean RR (m	826.978
STD RR (ms):	13.8197
Mean HR (be	72.5533
STD HR (beat	1.2209
Min HR (beat	69.7967
Max HR (bea	75.2936
RMSSD (ms):	15.3355
NNxx (beats)	0
pNNxx (%):	0
SDANN (ms):	
SDNN index (	

Geometric parameters

RR tri index:	4.270588	
TINN (ms):	67	
Frequency-Do	FFT spectrum	AR spectrum
Peak frequencies		
VLF (Hz):	0.04	0.04
LF (Hz):	0.08	0.103333
HF (Hz):	0.253333	0.273333
Absolute powers		
VLF (ms^2):	4.8097	11.6775
LF (ms^2):	58.542	49.7054
HF (ms^2):	117.4809	112.8776
VLF (log):	1.5706	2.4577
LF (log):	4.0697	3.9061
HF (log):	4.7663	4.7263
Relative powers		
VLF (%):	2.6593	6.6982
LF (%):	32.3679	28.5111
HF (%):	64.9552	64.7469
Normalized powers		
LF (n.u.):	33.2521	30.558
HF (n.u.):	66.7297	69.3951
Total power (	180.8645	174.3367
LF/HF ratio:	0.4983	0.4403

Nonlinear Results	
Poincare plot	
SD1 (ms):	10.858874
SD2 (ms):	16.262917
SD2/SD1 rati	1.497662
Approximate	1.182
Sample entro	2.0604

Detrended fluctuation analysis (DFA)

alpha 1:	0.7088
alpha 2:	0.2848

RR INTERVAL DATA and SPECTRUM ESTIMATES

SAMPLE 1

RR Data		FFT spectrum		AR Spectrum				
Time (s)	RR interval (s)	Frequency (Hz)	PSD (ms^2/Hz)	Frequency (Hz)	PSD (ms^2/Hz)	VLF comp. (ms^2/Hz)	LF comp. (ms^2/Hz)	HF comp. (ms^2/Hz)
61.257	0.822	0	4.1424	0	141.0559			
62.072	0.815	0.003	1.9122	0.003	282.4313			
62.901	0.829	0.007	0.2481	0.007	283.3911			
63.732	0.831	0.01	4.5826	0.01	284.9953			
64.545	0.813	0.013	17.6224	0.013	287.2505			
65.364	0.819	0.017	3.7309	0.017	290.1657			
66.201	0.837	0.02	4.2648	0.02	293.7522			
67.041	0.84	0.023	100.3669	0.023	298.0228			
67.872	0.831	0.027	391.935	0.027	302.992			
68.702	0.83	0.03	328.877	0.03	308.6744			
69.55	0.848	0.033	243.0539	0.033	315.0847			
70.387	0.837	0.037	110.3977	0.037	322.2359			
71.199	0.812	0.04	462.2364	0.04	330.1383			
72.009	0.81	0.043	273.807	0.043	338.7974			
72.843	0.834	0.047	92.84	0.047	348.2118			
73.681	0.838	0.05	32.417	0.05	358.3705			
74.506	0.825	0.053	581.2842	0.053	369.2496			
75.312	0.806	0.057	236.8566	0.057	380.8087			
76.143	0.831	0.06	142.3157	0.06	392.9868			
76.98	0.837	0.063	410.8251	0.063	405.698			
77.823	0.843	0.067	170.7914	0.067	418.827			
78.651	0.828	0.07	16.0489	0.07	432.2254			
79.463	0.812	0.073	379.8347	0.073	445.7087			

80.293	0.83	0.077	3526.3893	0.077	459.0555
81.121	0.828	0.08	3745.1274	0.08	472.0082
81.945	0.824	0.083	2208.012	0.083	484.2787
82.745	0.8	0.087	949.9445	0.087	495.5568
83.566	0.821	0.09	75.5931	0.09	505.5234
84.407	0.841	0.093	248.5682	0.093	513.8678
85.246	0.839	0.097	666.3875	0.097	520.3075
86.063	0.817	0.1	453.8236	0.1	524.6091
86.868	0.805	0.103	27.1714	0.103	526.6072
87.706	0.838	0.107	68.7965	0.107	526.2193
88.548	0.842	0.11	78.536	0.11	523.4539
89.392	0.844	0.113	215.8737	0.113	518.4108
90.231	0.839	0.117	174.1536	0.117	511.2728
91.059	0.828	0.12	14.9662	0.12	502.2912
91.931	0.872	0.123	257.9572	0.123	491.7665
92.795	0.864	0.127	593.1903	0.127	480.0272
93.652	0.857	0.13	216.802	0.13	467.4095
94.508	0.856	0.133	55.4151	0.133	454.2399
95.357	0.849	0.137	463.62	0.137	440.8213
96.187	0.83	0.14	282.489	0.14	427.4248
97.031	0.844	0.143	377.2202	0.143	414.284
97.885	0.854	0.147	25.4514	0.147	401.5948
98.744	0.859	0.15	555.3341	0.15	389.5165
99.596	0.852	0.153	430.5299	0.153	378.1751
100.456	0.86	0.157	261.0428	0.157	367.6686
101.313	0.857	0.16	309.9853	0.16	358.0712
102.144	0.831	0.163	146.3303	0.163	349.4392
102.979	0.835	0.167	466.4455	0.167	341.8153
103.842	0.863	0.17	326.2127	0.17	335.2333
104.699	0.857	0.173	42.8144	0.173	329.7226
105.561	0.862	0.177	215.9565	0.177	325.3114
106.428	0.867	0.18	167.2749	0.18	322.0306
107.263	0.835	0.183	612.593	0.183	319.9171

108.086	0.823	0.187	393.6729	0.187	319.0165
108.934	0.848	0.19	338.6674	0.19	319.3872
109.777	0.843	0.193	320.5761	0.193	321.1033
110.609	0.832	0.197	58.0175	0.197	324.259
111.419	0.81	0.2	249.8316	0.2	328.9734
112.248	0.829	0.203	679.3257	0.203	335.3959
113.097	0.849	0.207	693.4819	0.207	343.7139
113.951	0.854	0.21	25.033	0.21	354.161
114.776	0.825	0.213	316.6217	0.213	367.029
115.601	0.825	0.217	291.0541	0.217	382.6819
116.447	0.846	0.22	183.2491	0.22	401.5741
117.306	0.859	0.223	172.9485	0.223	424.2745
118.135	0.829	0.227	67.1022	0.227	451.4954
118.98	0.845	0.23	1326.7573	0.23	484.1294
119.841	0.861	0.233	2261.5362	0.233	523.2937
120.681	0.84	0.237	1352.6533	0.237	570.3781
121.498	0.817	0.24	1642.9565	0.24	627.091
122.336	0.838	0.243	1531.1573	0.243	695.4801
123.175	0.839	0.247	302.8548	0.247	777.8813
124.014	0.839	0.25	708.475	0.25	876.6947
124.829	0.815	0.253	3310.6576	0.253	993.7968
125.655	0.826	0.257	2867.2299	0.257	1129.2738
126.488	0.833	0.26	1052.2586	0.26	1279.1049
127.309	0.821	0.263	670.1818	0.263	1431.7847
128.103	0.794	0.267	194.3018	0.267	1565.3546
128.921	0.818	0.27	164.9547	0.27	1649.0084
129.76	0.839	0.273	1406.3126	0.273	1654.0085
130.6	0.84	0.277	2248.2073	0.277	1570.7171
131.42	0.82	0.28	1579.1547	0.28	1416.7974
132.254	0.834	0.283	1522.1004	0.283	1226.7632
133.079	0.825	0.287	242.618	0.287	1033.5504
133.897	0.818	0.29	1089.3588	0.29	857.9089
134.694	0.797	0.293	128.938	0.293	708.3178

135.486	0.792	0.297	713.0504	0.297	585.4364
136.294	0.808	0.3	163.6352	0.3	486.2981
137.121	0.827	0.303	14.4811	0.303	406.865
137.942	0.821	0.307	84.8545	0.307	343.2346
138.745	0.803	0.31	104.7993	0.31	292.0764
139.573	0.828	0.313	145.1297	0.313	250.7062
140.408	0.835	0.317	116.612	0.317	217.0194
141.242	0.834	0.32	80.2984	0.32	189.3868
142.064	0.822	0.323	48.234	0.323	166.5521
142.907	0.843	0.327	234.509	0.327	147.5454
143.752	0.845	0.33	21.6465	0.33	131.6156
144.582	0.83	0.333	82.5302	0.333	118.1775
145.393	0.811	0.337	33.0287	0.337	106.7724
146.216	0.823	0.34	3.8231	0.34	97.0384
147.042	0.826	0.343	63.8019	0.343	88.6879
147.869	0.827	0.347	98.9224	0.347	81.4908
148.666	0.797	0.35	34.375	0.35	75.262
149.479	0.813	0.353	58.5505	0.353	69.8513
150.314	0.835	0.357	7.208	0.357	65.1364
151.15	0.836	0.36	66.5174	0.36	61.0169
151.972	0.822	0.363	11.6861	0.363	57.4101
152.808	0.836	0.367	79.539	0.367	54.2478
153.656	0.848	0.37	38.1435	0.37	51.4729
154.51	0.854	0.373	9.715	0.373	49.0379
155.348	0.838	0.377	14.067	0.377	46.9033
156.184	0.836	0.38	93.7457	0.38	45.0356
157.037	0.853	0.383	127.1807	0.383	43.4069
157.896	0.859	0.387	21.1927	0.387	41.9938
158.748	0.852	0.39	22.7253	0.39	40.7765
159.57	0.822	0.393	23.6404	0.393	39.7386
160.407	0.837	0.397	25.281	0.397	38.8665
161.239	0.832	0.4	16.3092	0.4	38.1487
162.058	0.819	0.403	2.6889	0.403	37.5762

162.863	0.805	0.407	45.2564	0.407	37.1414
163.715	0.852	0.41	69.4085	0.41	36.8388
164.595	0.88	0.413	52.3056	0.413	36.6639
165.473	0.878	0.417	9.8426	0.417	36.6136
166.33	0.857	0.42	2.1018	0.42	36.6861
167.175	0.845	0.423	5.9376	0.423	36.8802
168.03	0.855	0.427	12.5018	0.427	37.1958
168.898	0.868	0.43	120.3682	0.43	37.6333
169.747	0.849	0.433	102.1597	0.433	38.1933
170.602	0.855	0.437	52.5937	0.437	38.8766
171.475	0.873	0.44	29.0359	0.44	39.6836
172.339	0.864	0.443	4.4631	0.443	40.6139
173.171	0.832	0.447	42.019	0.447	41.6655
173.991	0.82	0.45	25.3059	0.45	42.8339
174.833	0.842	0.453	4.0806	0.453	44.1115
175.687	0.854	0.457	19.3374	0.457	45.4857
176.543	0.856	0.46	47.879	0.46	46.9379
177.389	0.846	0.463	23.512	0.463	48.4418
178.222	0.833	0.467	15.23	0.467	49.9622
179.08	0.858	0.47	11.5409	0.47	51.4536
179.956	0.876	0.473	109.6043	0.473	52.86
180.842	0.886	0.477	76.2336	0.477	54.116
181.699	0.857	0.48	7.7209	0.48	55.1496
182.538	0.839	0.483	67.7998	0.483	55.8872
183.377	0.839	0.487	41.2963	0.487	56.2599
184.217	0.84	0.49	17.8257	0.49	56.212
185.064	0.847	0.493	12.7605	0.493	55.7089
185.899	0.835	0.497	68.5508	0.497	54.7428
186.714	0.815	0.5	101.2958	0.5	53.335
187.523	0.809				
188.36	0.837				
189.214	0.854				
190.067	0.853				

190.893	0.826
191.727	0.834
192.583	0.856
193.437	0.854
194.277	0.84
195.098	0.821
195.931	0.833
196.774	0.843
197.61	0.836
198.422	0.812
199.219	0.797
200.046	0.827
200.897	0.851
201.744	0.847
202.572	0.828
203.407	0.835
204.251	0.844
205.084	0.833
205.902	0.818
206.714	0.812
207.548	0.834
208.38	0.832
209.197	0.817
210.005	0.808
210.838	0.833
211.661	0.823
212.488	0.827
213.301	0.813
214.124	0.823
214.965	0.841
215.8	0.835
216.616	0.816
217.428	0.812

218.258	0.83
219.095	0.837
219.926	0.831
220.728	0.802
221.518	0.79
222.333	0.815
223.146	0.813
223.954	0.808
224.741	0.787
225.526	0.785
226.335	0.809
227.15	0.815
227.953	0.803
228.757	0.804
229.564	0.807
230.387	0.823
231.213	0.826
232.033	0.82
232.84	0.807
233.649	0.809
234.469	0.82
235.297	0.828
236.121	0.824
236.931	0.81
237.734	0.803
238.555	0.821
239.382	0.827
240.203	0.821
241.01	0.807
241.819	0.809
242.65	0.831
243.494	0.844
244.344	0.85

245.179	0.835
245.982	0.803
246.802	0.82
247.623	0.821
248.452	0.829
249.262	0.81
250.073	0.811
250.9	0.827
251.718	0.818
252.531	0.813
253.334	0.803
254.156	0.822
254.975	0.819
255.779	0.804
256.571	0.792
257.378	0.807
258.188	0.81
259.012	0.824
259.808	0.796
260.599	0.791
261.41	0.811
262.216	0.806
263.033	0.817
263.854	0.821
264.658	0.804
265.457	0.799
266.274	0.817
267.095	0.821
267.915	0.82
268.727	0.812
269.525	0.798
270.347	0.822
271.172	0.825

271.997	0.825
272.808	0.811
273.621	0.813
274.447	0.826
275.263	0.816
276.076	0.813
276.871	0.795
277.671	0.8
278.485	0.814
279.293	0.808
280.092	0.799
280.883	0.791
281.676	0.793
282.493	0.817
283.323	0.83
284.159	0.836
284.985	0.826
285.781	0.796
286.591	0.81
287.403	0.812
288.226	0.823
289.041	0.815
289.838	0.797
290.648	0.81
291.477	0.829
292.29	0.813
293.094	0.804
293.889	0.795
294.695	0.806
295.51	0.815
296.344	0.834
297.167	0.823
297.956	0.789

298.75	0.794
299.555	0.805
300.354	0.799
301.131	0.777
301.933	0.802
302.746	0.813
303.552	0.806
304.342	0.79
305.169	0.827
306.001	0.832
306.829	0.828
307.633	0.804
308.46	0.827
309.297	0.837
310.129	0.832
310.95	0.821
311.779	0.829
312.62	0.841
313.465	0.845
314.301	0.836
315.112	0.811
315.929	0.817
316.764	0.835
317.591	0.827
318.418	0.827
319.229	0.811
320.058	0.829
320.892	0.834
321.724	0.832
322.555	0.831
323.367	0.812
324.199	0.832
325.045	0.846

325.883	0.838
326.708	0.825
327.519	0.811
328.347	0.828
329.188	0.841
330.016	0.828
330.826	0.81
331.633	0.807
332.459	0.826
333.295	0.836
334.123	0.828
334.932	0.809
335.739	0.807
336.566	0.827
337.392	0.826
338.219	0.827
339.015	0.796
339.813	0.798
340.645	0.832
341.482	0.837
342.322	0.84
343.138	0.816
343.96	0.822
344.801	0.841
345.655	0.854
346.508	0.853
347.343	0.835
348.166	0.823
349.003	0.837
349.831	0.828
350.64	0.809
351.428	0.788
352.243	0.815

353.07	0.827
353.91	0.84
354.737	0.827
355.554	0.817
356.403	0.849
357.257	0.854
358.116	0.859
358.958	0.842
359.793	0.835
360.628	0.835

HRV ANALYSIS RESULTS - 09-Mar-2020 10:14:09
Kubios HRV Standard
3.3.1
released in August 2019

Analyzed by: Participant 4 - -

File name: C:\Users
Measurement date: xx/xx/xx xx:xx:xx
File type: asciiRR
Channel label: RR data
Data length: 00:06:00 (h:min:s)
Measurement rate: -

Parameters
 Number of samples: 1
 Detrending method: Smoothn priors (lambda: 500)
 Min/Max HR as average of: 5 beats
 Threshold for NNxx/pNNxx: 50 ms
 Frequency bands
  VLF: 0 - 0.04 Hz
  LF: 0.04 - 0.15 Hz
  HF: 0.15 - 0.4 Hz
 Interpolation rate: 4 Hz
 Points in frequency-domain: 300 points/Hz
 FFT spectrum options
  Window width: 300 s
  Window overlap: 50 %
 AR spectrum options
  AR model order: 16
  Use factorization: No
 Apply detrending for nonlinear analysis: 1
 Entropy embedding dimension: 2

Entropy	tolerance: 0.2 x SD
DFA	short-term fluctuations: 4-12 beats
DFA	long-term fluctuations: 13-64 beats

RR Interval Samples Selected for Analysis

	Sample 1
Sample limit:	00:01:00-00:06:00
Sample Analysis Type:	Single sample
Artifact correction:	none
Artifacts (%):	-

RESULTS FOR A SINGLE SAMPLE

Results Overview

PNS index:	-0.3937
SNS index:	0.7644
Stress index:	14.4452

Time-Domain Results

Statistical parameters

Mean RR (m	909.4711
STD RR (ms):	28.5811
Mean HR (be	65.9724
STD HR (beat	2.1041
Min HR (beat	62.967
Max HR (bea	73.3532
RMSSD (ms):	28.1068
NNxx (beats)	19
pNNxx (%):	5.7927
SDANN (ms):	
SDNN index (	

Geometric parameters

RR tri index:	6.58	
TINN (ms):	131	
Frequency-Do	FFT spectrum	AR spectrum
Peak frequencies		
VLF (Hz):	0.04	0.04
LF (Hz):	0.096667	0.09
HF (Hz):	0.396667	0.15
Absolute powers		
VLF (ms^2):	17.8656	63.2486
LF (ms^2):	520.0288	451.3042
HF (ms^2):	207.4398	183.1683
VLF (log):	2.8829	4.1471
LF (log):	6.2539	6.1121
HF (log):	5.3348	5.2104
Relative powers		
VLF (%):	2.3879	9.0286
LF (%):	69.5054	64.4229
HF (%):	27.7257	26.147
Normalized powers		
LF (n.u.):	71.2057	70.8167
HF (n.u.):	28.404	28.742
Total power (	748.1847	700.5338
LF/HF ratio:	2.5069	2.4639
Nonlinear Results		
Poincare plot		
SD1 (ms):	19.90545	
SD2 (ms):	35.144931	
SD2/SD1 rati	1.765593	
Approximate	1.0833	
Sample entro	1.7039	

Detrended fluctuation analysis (DFA)

alpha 1:	1.2162
alpha 2:	0.3064

RR INTERVAL DATA and SPECTRUM ESTIMATES

SAMPLE 1

RR Data		FFT spectrum		AR Spectrum				
Time (s)	RR interval (s)	Frequency (Hz)	PSD (ms^2/Hz)	Frequency (Hz)	PSD (ms^2/Hz)	VLF comp. (ms^2/Hz)	LF comp. (ms^2/Hz)	HF comp. (ms^2/Hz)
60.609	0.814	0	63.2574	0	700.5852			
61.435	0.826	0.003	28.8948	0.003	1404.9476			
62.27	0.835	0.007	0.2255	0.007	1416.3513			
63.071	0.801	0.01	0.3552	0.01	1435.5996			
63.879	0.808	0.013	25.7941	0.013	1463.0639			
64.692	0.813	0.017	90.9058	0.017	1499.2818			
65.511	0.819	0.02	79.86	0.02	1544.9747			
66.362	0.851	0.023	70.5404	0.023	1601.0722			
67.22	0.858	0.027	1160.7584	0.027	1668.7428			
68.03	0.81	0.03	927.2027	0.03	1749.4329			
68.81	0.78	0.033	937.1399	0.033	1844.9119			
69.609	0.799	0.037	392.1812	0.037	1957.3265			
70.422	0.813	0.04	3177.3856	0.04	2089.2583			
71.277	0.855	0.043	1512.8081	0.043	2243.7819			
72.146	0.869	0.047	2840.9051	0.047	2424.5113			
73.019	0.873	0.05	847.3832	0.05	2635.6124			
73.927	0.908	0.053	1929.6885	0.053	2881.7433			
74.872	0.945	0.057	10976.6552	0.057	3167.8521			
75.827	0.955	0.06	6807.2332	0.06	3498.7232			
76.761	0.934	0.063	584.2914	0.063	3878.1045			
77.675	0.914	0.067	3749.8328	0.067	4307.196			
78.58	0.905	0.07	4755.0512	0.07	4782.2855			
79.476	0.896	0.073	871.205	0.073	5291.4933			

80.35	0.874	0.077	4026.3243	0.077	5811.1336
81.244	0.894	0.08	3099.767	0.08	6303.2437
82.126	0.882	0.083	2363.6595	0.083	6717.0443
83	0.874	0.087	12019.0883	0.087	6997.1727
83.904	0.904	0.09	12224.1941	0.09	7098.6074
84.84	0.936	0.093	14037.4383	0.093	7002.5524
85.749	0.909	0.097	18973.4204	0.097	6724.0343
86.668	0.919	0.1	11431.1129	0.1	6306.02
87.571	0.903	0.103	6719.4506	0.103	5804.0334
88.452	0.881	0.107	11403.0839	0.107	5270.5038
89.369	0.917	0.11	3687.2853	0.11	4745.437
90.273	0.904	0.113	205.3726	0.113	4254.1987
91.154	0.881	0.117	511.5206	0.117	3809.7241
92.043	0.889	0.12	1073.7319	0.12	3416.213
92.926	0.883	0.123	1633.0086	0.123	3072.5553
93.802	0.876	0.127	5089.8696	0.127	2774.8616
94.709	0.907	0.13	4311.2234	0.13	2518.093
95.618	0.909	0.133	2301.9812	0.133	2297.0073
96.511	0.893	0.137	912.0562	0.137	2106.6513
97.429	0.918	0.14	865.4823	0.14	1942.5781
98.304	0.875	0.143	121.092	0.143	1800.9121
99.187	0.883	0.147	2187.2293	0.147	1678.3333
100.087	0.9	0.15	751.7047	0.15	1572.0259
100.996	0.909	0.153	3121.138	0.153	1479.6131
101.918	0.922	0.157	1862.518	0.157	1399.0923
102.877	0.959	0.16	1439.3581	0.16	1328.7744
103.787	0.91	0.163	1117.9615	0.163	1267.231
104.719	0.932	0.167	1649.4391	0.167	1213.2483
105.63	0.911	0.17	1070.3737	0.17	1165.7891
106.516	0.886	0.173	796.3391	0.173	1123.9608
107.423	0.907	0.177	2206.365	0.177	1086.9883
108.33	0.907	0.18	965.4618	0.18	1054.1927
109.217	0.887	0.183	1518.4673	0.183	1024.9728

110.121	0.904	0.187	4151.1142	0.187	998.7909
111.015	0.894	0.19	2162.3354	0.19	975.1613
111.885	0.87	0.193	1415.1456	0.193	953.641
112.802	0.917	0.197	364.2161	0.197	933.8235
113.723	0.921	0.2	33.5033	0.2	915.3342
114.607	0.884	0.203	25.6745	0.203	897.8278
115.513	0.906	0.207	51.5935	0.207	880.9883
116.418	0.905	0.21	452.5744	0.21	864.5288
117.284	0.866	0.213	252.0384	0.213	848.1947
118.163	0.879	0.217	59.0504	0.217	831.766
119.041	0.878	0.22	617.11	0.22	815.0606
119.941	0.9	0.223	1751.0394	0.223	797.9379
120.879	0.938	0.227	997.1747	0.227	780.3004
121.795	0.916	0.23	181.894	0.23	762.0957
122.745	0.95	0.233	768.4989	0.233	743.3155
123.658	0.913	0.237	2741.6669	0.237	723.9935
124.515	0.857	0.24	1593.3629	0.24	704.2014
125.419	0.904	0.243	624.6167	0.243	684.0428
126.328	0.909	0.247	627.7829	0.247	663.6468
127.26	0.932	0.25	254.3576	0.25	643.1598
128.209	0.949	0.253	31.5371	0.253	622.7382
129.106	0.897	0.257	54.5862	0.257	602.5406
130.032	0.926	0.26	840.0081	0.26	582.7219
130.947	0.915	0.263	751.6487	0.263	563.4276
131.86	0.913	0.267	113.2276	0.267	544.7901
132.798	0.938	0.27	220.2393	0.27	526.9264
133.715	0.917	0.273	1817.4676	0.273	509.9369
134.641	0.926	0.277	1490.4111	0.277	493.905
135.59	0.949	0.28	406.3809	0.28	478.8988
136.499	0.909	0.283	1704.2369	0.283	464.9719
137.448	0.949	0.287	2227.4801	0.287	452.1659
138.443	0.995	0.29	1014.3914	0.29	440.5125
139.38	0.937	0.293	545.0085	0.293	430.036

140.319	0.939	0.297	883.8548	0.297	420.7561
141.239	0.92	0.3	1512.1552	0.3	412.6899
142.16	0.921	0.303	331.7251	0.303	405.8547
143.098	0.938	0.307	86.1485	0.307	400.27
143.953	0.855	0.31	177.6894	0.31	395.9601
144.826	0.873	0.313	93.7894	0.313	392.9563
145.73	0.904	0.317	28.1005	0.317	391.2992
146.614	0.884	0.32	87.8955	0.32	391.0414
147.546	0.932	0.323	314.5023	0.323	392.25
148.484	0.938	0.327	18.1037	0.327	395.0099
149.421	0.937	0.33	319.9663	0.33	399.4272
150.408	0.987	0.333	110.6921	0.333	405.6334
151.331	0.923	0.337	48.1682	0.337	413.7904
152.262	0.931	0.34	257.7515	0.34	424.0961
153.209	0.947	0.343	467.1648	0.343	436.7916
154.12	0.911	0.347	191.6176	0.347	452.1695
155.079	0.959	0.35	90.0924	0.35	470.5833
156.065	0.986	0.353	3.9196	0.353	492.4587
157.012	0.947	0.357	25.3657	0.357	518.3055
157.959	0.947	0.36	125.7395	0.36	548.7295
158.908	0.949	0.363	452.2798	0.363	584.4429
159.859	0.951	0.367	779.6096	0.367	626.2663
160.819	0.96	0.37	160.3144	0.37	675.1176
161.755	0.936	0.373	152.3975	0.373	731.9696
162.713	0.958	0.377	297.1798	0.377	797.7521
163.654	0.941	0.38	133.443	0.38	873.1592
164.585	0.931	0.383	216.4004	0.383	958.3039
165.528	0.943	0.387	332.4001	0.387	1052.1568
166.452	0.924	0.39	1601.4537	0.39	1151.7433
167.368	0.916	0.393	336.0568	0.393	1251.2057
168.292	0.924	0.397	6309.3798	0.397	1341.1441
169.193	0.901	0.4	969.1791	0.4	1409.0428
170.092	0.899	0.403	1710.5997	0.403	1441.6793

171.014	0.922	0.407	1142.6119	0.407	1429.4726
171.922	0.908	0.41	119.7524	0.41	1370.8088
172.842	0.92	0.413	14.4465	0.413	1273.3227
173.785	0.943	0.417	713.2269	0.417	1150.9392
174.7	0.915	0.42	1557.4876	0.42	1018.7561
175.621	0.921	0.423	357.8921	0.423	888.959
176.552	0.931	0.427	405.6466	0.427	769.2546
177.466	0.914	0.43	255.3874	0.43	663.2944
178.394	0.928	0.433	40.2575	0.433	571.9065
179.311	0.917	0.437	271.5322	0.437	494.3053
180.219	0.908	0.44	224.5646	0.44	428.9653
181.139	0.92	0.443	14.9165	0.443	374.1477
182.033	0.894	0.447	202.8658	0.447	328.1769
182.944	0.911	0.45	373.2581	0.45	289.5597
183.857	0.913	0.453	184.866	0.453	257.0217
184.758	0.901	0.457	265.8377	0.457	229.501
185.677	0.919	0.46	204.3713	0.46	206.124
186.585	0.908	0.463	224.6815	0.463	186.1779
187.524	0.939	0.467	115.1271	0.467	169.0819
188.458	0.934	0.47	38.0267	0.47	154.3635
189.386	0.928	0.473	234.5052	0.473	141.637
190.305	0.919	0.477	542.4775	0.477	130.5874
191.216	0.911	0.48	821.3292	0.48	120.9559
192.146	0.93	0.483	909.071	0.483	112.5293
193.067	0.921	0.487	440.1538	0.487	105.131
194.005	0.938	0.49	108.3926	0.49	98.6143
194.928	0.923	0.493	40.5053	0.493	92.8565
195.864	0.936	0.497	35.9574	0.497	87.7543
196.79	0.926	0.5	74.1312	0.5	83.2207
197.724	0.934				
198.639	0.915				
199.549	0.91				
200.475	0.926				

201.387	0.912
202.336	0.949
203.28	0.944
204.221	0.941
205.158	0.937
206.083	0.925
207.019	0.936
207.939	0.92
208.88	0.941
209.816	0.936
210.75	0.934
211.71	0.96
212.618	0.908
213.516	0.898
214.422	0.906
215.292	0.87
216.124	0.832
216.984	0.86
217.825	0.841
218.799	0.974
219.709	0.91
220.621	0.912
221.511	0.89
222.466	0.955
223.408	0.942
224.329	0.921
225.256	0.927
226.191	0.935
227.093	0.902
227.983	0.89
228.901	0.918
229.803	0.902
230.721	0.918

231.651	0.93
232.585	0.934
233.521	0.936
234.375	0.854
235.244	0.869
236.101	0.857
236.937	0.836
237.748	0.811
238.598	0.85
239.535	0.937
240.5	0.965
241.468	0.968
242.395	0.927
243.322	0.927
244.25	0.928
245.12	0.87
246.002	0.882
246.841	0.839
247.638	0.797
248.456	0.818
249.318	0.862
250.241	0.923
251.183	0.942
252.154	0.971
253.074	0.92
253.983	0.909
254.865	0.882
255.77	0.905
256.696	0.926
257.596	0.9
258.492	0.896
259.368	0.876
260.242	0.874

261.161	0.919
262.102	0.941
263.048	0.946
264.03	0.982
264.981	0.951
265.919	0.938
266.834	0.915
267.716	0.882
268.636	0.92
269.545	0.909
270.42	0.875
271.34	0.92
272.26	0.92
273.181	0.921
274.118	0.937
275.007	0.889
275.928	0.921
276.882	0.954
277.794	0.912
278.732	0.938
279.657	0.925
280.581	0.924
281.538	0.957
282.459	0.921
283.395	0.936
284.311	0.916
285.197	0.886
286.102	0.905
286.997	0.895
287.872	0.875
288.778	0.906
289.694	0.916
290.602	0.908

291.523	0.921
292.423	0.9
293.335	0.912
294.248	0.913
295.128	0.88
296.038	0.91
296.958	0.92
297.866	0.908
298.84	0.974
299.779	0.939
300.626	0.847
301.478	0.852
302.355	0.877
303.23	0.875
304.133	0.903
305.08	0.947
306.018	0.938
306.997	0.979
307.941	0.944
308.853	0.912
309.804	0.951
310.738	0.934
311.691	0.953
312.639	0.948
313.569	0.93
314.534	0.965
315.448	0.914
316.347	0.899
317.252	0.905
318.101	0.849
318.946	0.845
319.784	0.838
320.621	0.837

321.484	0.863
322.336	0.852
323.224	0.888
324.148	0.924
325.055	0.907
326.015	0.96
326.946	0.931
327.841	0.895
328.745	0.904
329.621	0.876
330.515	0.894
331.404	0.889
332.275	0.871
333.183	0.908
334.105	0.922
335.043	0.938
335.945	0.902
336.829	0.884
337.736	0.907
338.693	0.957
339.605	0.912
340.546	0.941
341.469	0.923
342.391	0.922
343.365	0.974
344.297	0.932
345.212	0.915
346.149	0.937
347.06	0.911
347.994	0.934
348.93	0.936
349.846	0.916
350.763	0.917

351.657	0.894
352.554	0.897
353.452	0.898
354.359	0.907
355.281	0.922
356.194	0.913
357.125	0.931
358.063	0.938
359.011	0.948

HRV ANALYSIS RESULTS - 02-Mar-2020 12:37:00
Kubios HRV Standard
3.3.1
released in August 2019

Analyzed by: Participant 5 - -

File name: C:\Users
Measurement date: xx/xx/xx xx:xx:xx
File type: asciiRR
Channel label: RR data
Data length: 00:05:56 (h:min:s)
Measurement rate: -

Parameters
 Number of samples: 1
 Detrending method: Smoothn priors (lambda: 500)
 Min/Max HR as average of: 5 beats
 Threshold for NNxx/pNNxx: 50 ms
 Frequency bands
  VLF: 0 - 0.04 Hz
  LF: 0.04 - 0.15 Hz
  HF: 0.15 - 0.4 Hz
 Interpolation rate: 4 Hz
 Points in frequency-domain: 300 points/Hz
 FFT spectrum options
  Window width: 300 s
  Window overlap: 50 %
 AR spectrum options
  AR model order: 16
  Use factorization: No
 Apply detrending for nonlinear analysis: 1
 Entropy embedding dimension: 2

Entropy	tolerance: 0.2 x SD
DFA	short-term fluctuations: 4-12 beats
DFA	long-term fluctuations: 13-64 beats

RR Interval Samples Selected for Analysis

	Sample 1
Sample limit:	00:00:56-00:05:56

Sample Analysis Type: Single sample
Artifact correction: none

Artifacts (%):	-

RESULTS FOR A SINGLE SAMPLE

Results Overview

PNS index:	-1.0071
SNS index:	0.7953
Stress index:	9.5049

Time-Domain Results
Statistical parameters

Mean RR (m	783.5535
STD RR (ms):	46.7917
Mean HR (be	76.5742
STD HR (beat	4.5425
Min HR (beat	68.2594
Max HR (bea	87.0423
RMSSD (ms):	30.9494
NNxx (beats)	37
pNNxx (%):	9.6859
SDANN (ms):	
SDNN index (	

Geometric parameters

RR tri index:	10.942857	
TINN (ms):	210	
Frequency-Do	FFT spectrum	AR spectrum
Peak frequencies		
VLF (Hz):	0.033333	0.04
LF (Hz):	0.126667	0.12
HF (Hz):	0.16	0.15
Absolute powers		
VLF (ms^2):	88.5047	67.713
LF (ms^2):	1917.1909	1836.74
HF (ms^2):	227.8133	311.0828
VLF (log):	4.4831	4.2153
LF (log):	7.5586	7.5157
HF (log):	5.4285	5.7401
Relative powers		
VLF (%):	3.9625	3.0561
LF (%):	85.8364	82.8985
HF (%):	10.1997	14.0403
Normalized powers		
LF (n.u.):	89.378	85.5118
HF (n.u.):	10.6205	14.4829
Total power (	2233.5406	2215.6496
LF/HF ratio:	8.4156	5.9043

Nonlinear Results	
Poincare plot	
SD1 (ms):	21.913247
SD2 (ms):	62.508534
SD2/SD1 rati	2.852545
Approximate	0.8466
Sample entro	1.0029

Detrended fluctuation analysis (DFA)

alpha 1:	1.4673
alpha 2:	0.2032

RR INTERVAL DATA and SPECTRUM ESTIMATES

SAMPLE 1

RR Data		FFT spectrum		AR Spectrum				
Time (s)	RR interval (s)	Frequency (Hz)	PSD (ms^2/Hz)	Frequency (Hz)	PSD (ms^2/Hz)	VLF comp. (ms^2/Hz)	LF comp. (ms^2/Hz)	HF comp. (ms^2/Hz)
56.43	0.789	0	69.9987	0	787.8281			
57.222	0.792	0.003	34.1787	0.003	1578.4555			
58.016	0.794	0.007	3.6018	0.007	1586.8946			
58.801	0.785	0.01	1.5169	0.01	1601.0972			
59.555	0.754	0.013	57.9929	0.013	1621.2745			
60.306	0.751	0.017	223.1202	0.017	1647.7311			
61.051	0.745	0.02	774.1873	0.02	1680.875			
61.822	0.771	0.023	962.3361	0.023	1721.2313			
62.659	0.837	0.027	1029.5961	0.027	1769.4599			
63.514	0.855	0.03	7781.4845	0.03	1826.3794			
64.326	0.812	0.033	12164.7452	0.033	1892.9974			
65.088	0.762	0.037	3472.9614	0.037	1970.5501			
65.823	0.735	0.04	35.914	0.04	2060.5539			
66.552	0.729	0.043	2203.7733	0.043	2164.8721			
67.282	0.73	0.047	3204.5801	0.047	2285.8025			
68.03	0.748	0.05	2109.5279	0.05	2426.1933			
68.794	0.764	0.053	1372.7746	0.053	2589.5977			
69.585	0.791	0.057	6210.9302	0.057	2780.4811			
70.367	0.782	0.06	9473.4084	0.06	3004.5046			
71.157	0.79	0.063	4813.8928	0.063	3268.9136			
71.97	0.813	0.067	1733.5004	0.067	3583.0817			
72.813	0.843	0.07	11337.4144	0.07	3959.2781			
73.644	0.831	0.073	9727.4688	0.073	4413.7685			

74.438	0.794	0.077	3086.8378	0.077	4968.4163
75.204	0.766	0.08	1311.4265	0.08	5653.0439
75.961	0.757	0.083	10804.9182	0.083	6508.9609
76.72	0.759	0.087	5984.3989	0.087	7594.2903
77.487	0.767	0.09	7030.6828	0.09	8992.0332
78.259	0.772	0.093	32240.6253	0.093	10822.1554
79.016	0.757	0.097	41634.1584	0.097	13258.9399
79.77	0.754	0.1	9880.3478	0.1	16552.8268
80.54	0.77	0.103	9917.0869	0.103	21046.4281
81.378	0.838	0.107	21593.0414	0.107	27141.011
82.261	0.883	0.11	13693.1953	0.11	35079.0075
83.117	0.856	0.113	946.1933	0.113	44275.9362
83.934	0.817	0.117	8178.4254	0.117	52232.9099
84.711	0.777	0.12	24679.6315	0.12	54856.0227
85.462	0.751	0.123	52745.4381	0.123	50346.6825
86.208	0.746	0.127	121786.657	0.127	41589.4748
86.955	0.747	0.13	87148.5223	0.13	32514.5267
87.715	0.76	0.133	1566.5836	0.133	25030.0382
88.517	0.802	0.137	14462.8819	0.137	19391.546
89.37	0.853	0.14	15725.046	0.14	15262.7108
90.222	0.852	0.143	34350.689	0.143	12240.6592
91.043	0.821	0.147	2880.6021	0.147	10003.1756
91.827	0.784	0.15	3080.3179	0.15	8319.9953
92.584	0.757	0.153	752.9425	0.153	7032.1779
93.324	0.74	0.157	5692.1075	0.157	6030.5487
94.062	0.738	0.16	13198.6301	0.16	5239.5241
94.795	0.733	0.163	7370.7851	0.163	4606.0729
95.544	0.749	0.167	592.7904	0.167	4092.4079
96.333	0.789	0.17	337.1772	0.17	3671.1716
97.179	0.846	0.173	4.552	0.173	3322.245
98.119	0.94	0.177	1129.2601	0.177	3030.6091
99.034	0.915	0.18	289.5899	0.18	2784.8908
99.879	0.845	0.183	1069.7161	0.183	2576.3604

100.663	0.784	0.187	667.7441	0.187	2398.2299
101.411	0.748	0.19	569.6739	0.19	2245.1541
102.14	0.729	0.193	2513.7568	0.193	2112.8716
102.873	0.733	0.197	1299.5636	0.197	1997.9422
103.641	0.768	0.2	69.3313	0.2	1897.5527
104.431	0.79	0.203	9.0957	0.203	1809.3712
105.265	0.834	0.207	58.1514	0.207	1731.4362
106.122	0.857	0.21	141.7776	0.21	1662.0728
106.968	0.846	0.213	490.0463	0.213	1599.8273
107.804	0.836	0.217	117.3909	0.217	1543.4172
108.617	0.813	0.22	387.9844	0.22	1491.6939
109.446	0.829	0.223	1901.1623	0.223	1443.6153
110.266	0.82	0.227	2870.7464	0.227	1398.2271
111.056	0.79	0.23	3312.4139	0.23	1354.6534
111.808	0.752	0.233	770.827	0.233	1312.0938
112.539	0.731	0.237	257.468	0.237	1269.8288
113.271	0.732	0.24	373.0622	0.24	1227.2307
114.003	0.732	0.243	1073.3046	0.243	1183.7784
114.737	0.734	0.247	3493.7452	0.247	1139.0756
115.477	0.74	0.25	1208.518	0.25	1092.8663
116.22	0.743	0.253	5.2505	0.253	1045.0475
116.992	0.772	0.257	483.367	0.257	995.6726
117.801	0.809	0.26	143.6551	0.26	944.9455
118.646	0.845	0.263	1000.7015	0.263	893.2031
119.491	0.845	0.267	674.6287	0.267	840.8871
120.301	0.81	0.27	2170.0783	0.27	788.5086
121.072	0.771	0.273	2468.406	0.273	736.6087
121.815	0.743	0.277	652.9806	0.277	685.7202
122.541	0.726	0.28	214.8799	0.28	636.3339
123.268	0.727	0.283	964.6881	0.283	588.8739
124.001	0.733	0.287	704.6479	0.287	543.6807
124.753	0.752	0.29	39.6734	0.29	501.005
125.543	0.79	0.293	494.7637	0.293	461.0084

126.366	0.823	0.297	312.7758	0.297	423.7708
127.221	0.855	0.3	95.543	0.3	389.3011
128.072	0.851	0.303	211.776	0.303	357.5505
128.884	0.812	0.307	206.4849	0.307	328.4249
129.661	0.777	0.31	54.0489	0.31	301.7983
130.422	0.761	0.313	14.9765	0.313	277.5229
131.17	0.748	0.317	123.5363	0.317	255.4387
131.916	0.746	0.32	119.5363	0.32	235.3806
132.663	0.747	0.323	9.8639	0.323	217.184
133.441	0.778	0.327	459.5943	0.327	200.689
134.232	0.791	0.33	792.5377	0.33	185.7429
134.996	0.764	0.333	84.665	0.333	172.2022
135.725	0.729	0.337	70.4115	0.337	159.9335
136.438	0.713	0.34	24.581	0.34	148.8141
137.137	0.699	0.343	24.2591	0.343	138.7314
137.833	0.696	0.347	108.7839	0.347	129.5834
138.539	0.706	0.35	56.462	0.35	121.2774
139.273	0.734	0.353	103.6729	0.353	113.7301
140.062	0.789	0.357	115.6913	0.357	106.8664
140.898	0.836	0.36	163.8469	0.36	100.6186
141.758	0.86	0.363	83.8779	0.363	94.9264
142.595	0.837	0.367	262.9574	0.367	89.7355
143.393	0.798	0.37	190.3967	0.37	84.9975
144.169	0.776	0.373	15.9679	0.373	80.6688
144.925	0.756	0.377	221.1531	0.377	76.7106
145.655	0.73	0.38	164.1725	0.38	73.0881
146.393	0.738	0.383	19.6531	0.383	69.7701
147.154	0.761	0.387	108.854	0.387	66.7287
147.991	0.837	0.39	263.4838	0.39	63.9387
148.867	0.876	0.393	160.2697	0.393	61.3777
149.749	0.882	0.397	21.3426	0.397	59.0254
150.558	0.809	0.4	13.4165	0.4	56.8635
151.323	0.765	0.403	37.6848	0.403	54.8755

152.052	0.729	0.407	67.0738	0.407	53.0466
152.753	0.701	0.41	46.4471	0.41	51.3634
153.445	0.692	0.413	22.6246	0.413	49.8135
154.159	0.714	0.417	14.4218	0.417	48.3857
154.98	0.821	0.42	25.5246	0.42	47.07
155.947	0.967	0.423	68.6715	0.423	45.8569
156.877	0.93	0.427	27.9934	0.427	44.7379
157.784	0.907	0.43	56.7069	0.43	43.7049
158.66	0.876	0.433	80.9942	0.433	42.7505
159.506	0.846	0.437	92.4548	0.437	41.8679
160.314	0.808	0.44	97.5793	0.44	41.0505
161.119	0.805	0.443	29.4444	0.443	40.2922
161.941	0.822	0.447	13.6977	0.447	39.587
162.814	0.873	0.45	39.5225	0.45	38.9294
163.698	0.884	0.453	98.5819	0.453	38.314
164.548	0.85	0.457	29.1592	0.457	37.7354
165.365	0.817	0.46	15.7266	0.46	37.1886
166.167	0.802	0.463	140.9324	0.463	36.6686
166.957	0.79	0.467	97.1786	0.467	36.1705
167.744	0.787	0.47	7.3315	0.47	35.6895
168.534	0.79	0.473	21.3622	0.473	35.2209
169.342	0.808	0.477	45.0412	0.477	34.7601
170.195	0.853	0.48	30.022	0.48	34.3028
171.077	0.882	0.483	5.8074	0.483	33.8446
171.926	0.849	0.487	26.55	0.487	33.3814
172.738	0.812	0.49	55.9297	0.49	32.9095
173.54	0.802	0.493	47.1687	0.493	32.4253
174.326	0.786	0.497	18.9706	0.497	31.9256
175.103	0.777	0.5	25.3795	0.5	31.4077
175.87	0.767				
176.63	0.76				
177.387	0.757				
178.15	0.763				

178.933	0.783
179.766	0.833
180.615	0.849
181.451	0.836
182.263	0.812
183.037	0.774
183.789	0.752
184.533	0.744
185.277	0.744
186.049	0.772
186.848	0.799
187.711	0.863
188.617	0.906
189.508	0.891
190.367	0.859
191.192	0.825
191.998	0.806
192.78	0.782
193.549	0.769
194.307	0.758
195.078	0.771
195.878	0.8
196.702	0.824
197.494	0.792
198.264	0.77
199.013	0.749
199.765	0.752
200.536	0.771
201.312	0.776
202.09	0.778
202.885	0.795
203.723	0.838
204.585	0.862

205.429	0.844
206.214	0.785
206.967	0.753
207.691	0.724
208.395	0.704
209.096	0.701
209.842	0.746
210.647	0.805
211.533	0.886
212.453	0.92
213.371	0.918
214.22	0.849
215.009	0.789
215.745	0.736
216.461	0.716
217.194	0.733
217.938	0.744
218.727	0.789
219.539	0.812
220.345	0.806
221.158	0.813
221.974	0.816
222.77	0.796
223.554	0.784
224.341	0.787
225.125	0.784
225.91	0.785
226.712	0.802
227.499	0.787
228.26	0.761
229.001	0.741
229.724	0.723
230.446	0.722

231.175	0.729
231.901	0.726
232.626	0.725
233.331	0.705
234.03	0.699
234.735	0.705
235.452	0.717
236.175	0.723
236.901	0.726
237.621	0.72
238.287	0.666
238.938	0.651
239.61	0.672
240.313	0.703
241.036	0.723
241.772	0.736
242.531	0.759
243.315	0.784
244.127	0.812
244.915	0.788
245.655	0.74
246.361	0.706
247.051	0.69
247.735	0.684
248.423	0.688
249.13	0.707
249.863	0.733
250.632	0.769
251.404	0.772
252.191	0.787
253.012	0.821
253.819	0.807
254.589	0.77

255.329	0.74
256.043	0.714
256.756	0.713
257.489	0.733
258.241	0.752
258.981	0.74
259.718	0.737
260.461	0.743
261.236	0.775
262.02	0.784
262.787	0.767
263.533	0.746
264.249	0.716
264.943	0.694
265.633	0.69
266.324	0.691
267.038	0.714
267.772	0.734
268.517	0.745
269.256	0.739
269.978	0.722
270.681	0.703
271.39	0.709
272.113	0.723
272.861	0.748
273.644	0.783
274.457	0.813
275.25	0.793
276.012	0.762
276.756	0.744
277.479	0.723
278.208	0.729
278.962	0.754

279.778	0.816
280.671	0.893
281.543	0.872
282.363	0.82
283.14	0.777
283.893	0.753
284.637	0.744
285.388	0.751
286.145	0.757
286.906	0.761
287.719	0.813
288.627	0.908
289.535	0.908
290.382	0.847
291.167	0.785
291.913	0.746
292.644	0.731
293.365	0.721
294.086	0.721
294.844	0.758
295.665	0.821
296.58	0.915
297.534	0.954
298.454	0.92
299.314	0.86
300.139	0.825
300.94	0.801
301.739	0.799
302.526	0.787
303.305	0.779
304.089	0.784
304.883	0.794
305.711	0.828

306.569	0.858
307.414	0.845
308.236	0.822
309.04	0.804
309.843	0.803
310.632	0.789
311.421	0.789
312.205	0.784
312.985	0.78
313.762	0.777
314.553	0.791
315.395	0.842
316.279	0.884
317.154	0.875
317.986	0.832
318.757	0.771
319.495	0.738
320.215	0.72
320.934	0.719
321.679	0.745
322.454	0.775
323.294	0.84
324.187	0.893
325.087	0.9
325.936	0.849
326.747	0.811
327.534	0.787
328.328	0.794
329.116	0.788
329.899	0.783
330.691	0.792
331.511	0.82
332.373	0.862

333.23	0.857
334.036	0.806
334.803	0.767
335.538	0.735
336.265	0.727
337.001	0.736
337.768	0.767
338.604	0.836
339.459	0.855
340.276	0.817
341.061	0.785
341.83	0.769
342.598	0.768
343.356	0.758
344.114	0.758
344.892	0.778
345.693	0.801
346.501	0.808
347.284	0.783
348.035	0.751
348.763	0.728
349.491	0.728
350.221	0.73
350.967	0.746
351.738	0.771
352.553	0.815
353.388	0.835
354.213	0.825
354.992	0.779
355.742	0.75

HRV ANALYSIS RESULTS - 02-Mar-2020 12:40:29
Kubios HRV Standard
3.3.1
released in August 2019

Analyzed by: Participant 6 - -

File name: C:\Users
Measurement date: xx/xx/xx xx:xx:xx
File type: asciiRR
Channel label: RR data
Data length: 00:05:58 (h:min:s)
Measurement rate: -

Parameters
 Number of samples: 1
 Detrending method: Smoothn priors (lambda: 500)
 Min/Max HR as average of: 5 beats
 Threshold for NNxx/pNNxx: 50 ms
 Frequency bands
  VLF: 0 - 0.04 Hz
  LF: 0.04 - 0.15 Hz
  HF: 0.15 - 0.4 Hz
 Interpolation rate: 4 Hz
 Points in frequency-domain: 300 points/Hz
 FFT spectrum options
  Window width: 300 s
  Window overlap: 50 %
 AR spectrum options
  AR model order: 16
  Use factorization: No
 Apply detrending for nonlinear analysis: 1
 Entropy embedding dimension: 2

Entropy	tolerance: 0.2 x SD
DFA	short-term fluctuations: 4-12 beats
DFA	long-term fluctuations: 13-64 beats

RR Interval Samples Selected for Analysis

	Sample 1
Sample limit:	00:00:58-00:05:58
Sample Analysis Type:	Single sample
Artifact correction:	none
Artifacts (%):	-

RESULTS FOR A SINGLE SAMPLE

Results Overview

PNS index:	-0.1349
SNS index:	0.1987
Stress index:	11.2309

Time-Domain Results

Statistical parameters

Mean RR (m	926.6265
STD RR (ms):	36.7818
Mean HR (be	64.751
STD HR (beat	2.668
Min HR (beat	60.8396
Max HR (bea	72.0946
RMSSD (ms):	35.2787
NNxx (beats)	45
pNNxx (%):	13.9319
SDANN (ms):	
SDNN index (	

Geometric parameters

RR tri index:	7.902439	
TINN (ms):	164	
Frequency-Do	FFT spectrum	AR spectrum
Peak frequencies		
VLF (Hz):	0.04	0.04
LF (Hz):	0.083333	0.096667
HF (Hz):	0.156667	0.15
Absolute powers		
VLF (ms^2):	70.6212	109.7074
LF (ms^2):	984.5081	737.3617
HF (ms^2):	382.884	307.3606
VLF (log):	4.2573	4.6978
LF (log):	6.8921	6.6031
HF (log):	5.9477	5.728
Relative powers		
VLF (%):	4.8995	9.4917
LF (%):	68.3019	63.7954
HF (%):	26.5632	26.5924
Normalized powers		
LF (n.u.):	71.8207	70.4857
HF (n.u.):	27.9317	29.3811
Total power (	1441.4064	1155.8229
LF/HF ratio:	2.5713	2.399
Nonlinear Results		
Poincare plot		
SD1 (ms):	24.984733	
SD2 (ms):	45.60823	
SD2/SD1 rati	1.825444	
Approximate	1.1556	
Sample entro	1.896	

Detrended fluctuation analysis (DFA)

alpha 1:	1.1215
alpha 2:	0.3318

RR INTERVAL DATA and SPECTRUM ESTIMATES

SAMPLE 1

RR Data		FFT spectrum		AR Spectrum				
Time (s)	RR interval (s)	Frequency (Hz)	PSD (ms^2/Hz)	Frequency (Hz)	PSD (ms^2/Hz)	VLF comp. (ms^2/Hz)	LF comp. (ms^2/Hz)	HF comp. (ms^2/Hz)
58.293	0.906	0	38.5364	0	1247.3652			
59.237	0.944	0.003	17.6468	0.003	2500.3021			
60.171	0.934	0.007	0.5633	0.007	2517.1039			
61.068	0.897	0.01	5.3069	0.01	2545.3968			
61.999	0.931	0.013	108.8939	0.013	2585.6238			
62.928	0.929	0.017	561.4276	0.017	2638.4207			
63.865	0.937	0.02	2045.3042	0.02	2704.6325			
64.792	0.927	0.023	3563.567	0.023	2785.3343			
65.721	0.929	0.027	1396.0546	0.027	2881.8578			
66.657	0.936	0.03	1755.9642	0.03	2995.8232			
67.585	0.928	0.033	5759.7669	0.033	3129.1749			
68.53	0.945	0.037	395.6681	0.037	3284.2217			
69.487	0.957	0.04	10977.3348	0.04	3463.677			
70.42	0.933	0.043	13102.8305	0.043	3670.6951			
71.364	0.944	0.047	3476.4193	0.047	3908.8934			
72.203	0.839	0.05	1075.3019	0.05	4182.3443			
73.166	0.963	0.053	1388.751	0.053	4495.5121			
74.09	0.924	0.057	6101.3679	0.057	4853.0908			
75.015	0.925	0.06	28491.4238	0.06	5259.6805			
75.918	0.903	0.063	7200.6405	0.063	5719.2115			
76.812	0.894	0.067	213.2928	0.067	6233.9956			
77.719	0.907	0.07	7484.9348	0.07	6803.2743			
78.608	0.889	0.073	11784.627	0.073	7421.168			

79.51	0.902	0.077	4275.3853	0.077	8074.0864
80.446	0.936	0.08	5134.406	0.08	8738.0041
81.401	0.955	0.083	30926.9991	0.083	9376.5854
82.378	0.977	0.087	13998.356	0.087	9941.7817
83.337	0.959	0.09	3308.9234	0.09	10378.7049
84.286	0.949	0.093	5304.4179	0.093	10635.4642
85.231	0.945	0.097	11952.4772	0.097	10675.9523
86.193	0.962	0.1	24281.3896	0.1	10490.6181
87.13	0.937	0.103	10490.8136	0.103	10099.8123
88.067	0.937	0.107	709.979	0.107	9547.7826
89.029	0.962	0.11	12693.4497	0.11	8890.4795
89.955	0.926	0.113	12975.864	0.113	8182.9335
90.915	0.96	0.117	7533.9947	0.117	7470.6172
91.848	0.933	0.12	9791.6996	0.12	6785.9887
92.797	0.949	0.123	5065.231	0.123	6148.9887
93.744	0.947	0.127	3103.329	0.127	5569.5457
94.692	0.948	0.13	1646.5408	0.13	5050.5481
95.674	0.982	0.133	11805.1458	0.133	4590.4499
96.64	0.966	0.137	21338.1509	0.137	4185.2295
97.596	0.956	0.14	5809.6131	0.14	3829.7181
98.52	0.924	0.143	2072.3413	0.143	3518.4286
99.438	0.918	0.147	4697.6549	0.147	3246.0333
100.357	0.919	0.15	821.6867	0.15	3007.6104
101.259	0.902	0.153	3458.9067	0.153	2798.7476
102.165	0.906	0.157	13082.8242	0.157	2615.5642
103.08	0.915	0.16	6286.1339	0.16	2454.6879
103.934	0.854	0.163	169.2126	0.163	2313.2097
104.819	0.885	0.167	2197.3111	0.167	2188.6312
105.66	0.841	0.17	2641.8638	0.17	2078.8114
106.461	0.801	0.173	168.7667	0.173	1981.9164
107.326	0.865	0.177	1936.0899	0.177	1896.3738
108.183	0.857	0.18	2154.5589	0.18	1820.8335
109.11	0.927	0.183	1375.9351	0.183	1754.1327

110.021	0.911	0.187	1557.0385	0.187	1695.2667
110.935	0.914	0.19	409.6301	0.19	1643.3633
111.909	0.974	0.193	1263.7767	0.193	1597.6611
112.858	0.949	0.197	446.3271	0.197	1557.4915
113.81	0.952	0.2	1025.1976	0.2	1522.2626
114.714	0.904	0.203	817.1738	0.203	1491.4458
115.629	0.915	0.207	109.3084	0.207	1464.564
116.54	0.911	0.21	155.7168	0.21	1441.1818
117.419	0.879	0.213	2228.0074	0.213	1420.8968
118.28	0.861	0.217	5330.113	0.217	1403.3322
119.167	0.887	0.22	3569.2953	0.22	1388.1302
120.075	0.908	0.223	610.758	0.223	1374.9475
120.987	0.912	0.227	2029.1966	0.227	1363.4504
121.94	0.953	0.23	5632.9849	0.23	1353.3124
122.862	0.922	0.233	2344.9226	0.233	1344.2117
123.804	0.942	0.237	2713.5455	0.237	1335.8309
124.726	0.922	0.24	2098.5665	0.24	1327.8576
125.625	0.899	0.243	324.758	0.243	1319.9861
126.577	0.952	0.247	253.7134	0.247	1311.9204
127.534	0.957	0.25	1325.4005	0.25	1303.3789
128.439	0.905	0.253	1754.9104	0.253	1294.0994
129.37	0.931	0.257	3725.1959	0.257	1283.8454
130.252	0.882	0.26	1483.4496	0.26	1272.412
131.158	0.906	0.263	1064.9323	0.263	1259.6324
132.042	0.884	0.267	185.3507	0.267	1245.383
132.956	0.914	0.27	1314.9947	0.27	1229.5877
133.85	0.894	0.273	2250.0134	0.273	1212.2206
134.778	0.928	0.277	285.754	0.277	1193.3059
135.694	0.916	0.28	740.3473	0.28	1172.9169
136.523	0.829	0.283	1370.3029	0.283	1151.1713
137.353	0.83	0.287	172.1917	0.287	1128.2261
138.219	0.866	0.29	437.4115	0.29	1104.2699
139.091	0.872	0.293	902.4249	0.293	1079.5148

139.936	0.845	0.297	1379.8241	0.297	1054.1876
140.734	0.798	0.3	1538.4671	0.3	1028.5215
141.593	0.859	0.303	58.208	0.303	1002.7484
142.491	0.898	0.307	475.4493	0.307	977.0919
143.425	0.934	0.31	1653.3074	0.31	951.7621
144.373	0.948	0.313	3577.6758	0.313	926.9513
145.315	0.942	0.317	3709.8572	0.317	902.8317
146.28	0.965	0.32	1653.0529	0.32	879.5538
147.224	0.944	0.323	1843.3661	0.323	857.2462
148.166	0.942	0.327	2891.6177	0.327	836.016
149.104	0.938	0.33	1772.9921	0.33	815.9505
150.039	0.935	0.333	150.6608	0.333	797.1185
150.947	0.908	0.337	1049.7869	0.337	779.5727
151.867	0.92	0.34	117.2378	0.34	763.3513
152.771	0.904	0.343	988.1241	0.343	748.4809
153.679	0.908	0.347	763.3474	0.347	734.9776
154.617	0.938	0.35	119.1488	0.35	722.8499
155.567	0.95	0.353	496.4996	0.353	712.0991
156.51	0.943	0.357	396.932	0.357	702.7217
157.475	0.965	0.36	1802.1155	0.36	694.7097
158.451	0.976	0.363	803.4376	0.363	688.0518
159.419	0.968	0.367	159.3383	0.367	682.7335
160.376	0.957	0.37	478.3223	0.37	678.7373
161.335	0.959	0.373	525.3314	0.373	676.0421
162.298	0.963	0.377	72.1412	0.377	674.6227
163.202	0.904	0.38	437.1016	0.38	674.4483
164.181	0.979	0.383	286.1276	0.383	675.4809
165.11	0.929	0.387	462.519	0.387	677.6728
165.929	0.819	0.39	492.3557	0.39	680.9639
166.797	0.868	0.393	243.4634	0.393	685.2775
167.638	0.841	0.397	146.824	0.397	690.5168
168.44	0.802	0.4	1816.0277	0.4	696.5592
169.207	0.767	0.403	1892.8881	0.403	703.2514

170.007	0.8	0.407	94.5942	0.407	710.4039
170.923	0.916	0.41	318.7929	0.41	717.7851
171.884	0.961	0.413	847.1082	0.413	725.1173
172.841	0.957	0.417	891.7045	0.417	732.0741
173.801	0.96	0.42	2389.2402	0.42	738.2807
174.756	0.955	0.423	1559.1458	0.423	743.3185
175.589	0.833	0.427	177.7248	0.427	746.736
176.426	0.837	0.43	122.8502	0.43	748.0657
177.269	0.843	0.433	361.3974	0.433	746.8485
178.097	0.828	0.437	257.0803	0.437	742.6644
178.988	0.891	0.44	278.6563	0.44	735.1664
179.932	0.944	0.443	158.3613	0.443	724.1157
180.865	0.933	0.447	486.6512	0.447	709.4107
181.776	0.911	0.45	208.6476	0.45	691.1063
182.72	0.944	0.453	1030.516	0.453	669.4191
183.656	0.936	0.457	93.0358	0.457	644.7158
184.569	0.913	0.46	489.2826	0.46	617.4863
185.521	0.952	0.463	752.4194	0.463	588.3058
186.446	0.925	0.467	780.3502	0.467	557.7906
187.317	0.871	0.47	882.8788	0.47	526.5554
188.157	0.84	0.473	0.1168	0.473	495.1758
188.988	0.831	0.477	328.2507	0.477	464.1605
189.87	0.882	0.48	172.4036	0.48	433.9349
190.804	0.934	0.483	303.5675	0.483	404.8339
191.761	0.957	0.487	460.323	0.487	377.1036
192.745	0.984	0.49	363.2745	0.49	350.9087
193.684	0.939	0.493	17.0849	0.493	326.3438
194.651	0.967	0.497	298.7491	0.497	303.4452
195.612	0.961	0.5	175.7328	0.5	282.204
196.568	0.956				
197.511	0.943				
198.488	0.977				
199.492	1.004				

200.451	0.959
201.415	0.964
202.395	0.98
203.336	0.941
204.316	0.98
205.3	0.984
206.272	0.972
207.249	0.977
208.237	0.988
209.225	0.988
210.168	0.943
211.066	0.898
211.998	0.932
212.92	0.922
213.889	0.969
214.82	0.931
215.758	0.938
216.706	0.948
217.651	0.945
218.584	0.933
219.434	0.85
220.236	0.802
221.091	0.855
222.023	0.932
222.968	0.945
223.91	0.942
224.818	0.908
225.739	0.921
226.678	0.939
227.547	0.869
228.452	0.905
229.363	0.911
230.281	0.918

231.219	0.938
232.12	0.901
232.985	0.865
233.838	0.853
234.706	0.868
235.56	0.854
236.382	0.822
237.237	0.855
238.12	0.883
239.011	0.891
239.909	0.898
240.847	0.938
241.791	0.944
242.757	0.966
243.73	0.973
244.72	0.99
245.72	1
246.686	0.966
247.625	0.939
248.568	0.943
249.469	0.901
250.388	0.919
251.287	0.899
252.293	1.006
253.235	0.942
254.214	0.979
255.169	0.955
256.141	0.972
257.093	0.952
257.964	0.871
258.855	0.891
259.781	0.926
260.706	0.925

261.64	0.934
262.583	0.943
263.542	0.959
264.434	0.892
265.349	0.915
266.259	0.91
267.211	0.952
268.181	0.97
269.187	1.006
270.096	0.909
270.984	0.888
271.937	0.953
272.892	0.955
273.899	1.007
274.893	0.994
275.831	0.938
276.801	0.97
277.753	0.952
278.737	0.984
279.713	0.976
280.734	1.021
281.747	1.013
282.738	0.991
283.697	0.959
284.574	0.877
285.449	0.875
286.334	0.885
287.253	0.919
288.198	0.945
289.124	0.926
290.043	0.919
291.013	0.97
291.979	0.966

292.941	0.962
293.875	0.934
294.854	0.979
295.826	0.972
296.811	0.985
297.742	0.931
298.77	1.028
299.743	0.973
300.697	0.954
301.672	0.975
302.605	0.933
303.563	0.958
304.495	0.932
305.425	0.93
306.347	0.922
307.243	0.896
308.194	0.951
309.125	0.931
310.063	0.938
311.04	0.977
311.989	0.949
312.959	0.97
313.909	0.95
314.852	0.943
315.826	0.974
316.793	0.967
317.752	0.959
318.703	0.951
319.616	0.913
320.438	0.822
321.317	0.879
322.247	0.93
323.188	0.941

324.15	0.962
325.127	0.977
326.093	0.966
327.026	0.933
327.9	0.874
328.813	0.913
329.751	0.938
330.737	0.986
331.749	1.012
332.714	0.965
333.663	0.949
334.625	0.962
335.576	0.951
336.504	0.928
337.459	0.955
338.415	0.956
339.402	0.987
340.381	0.979
341.362	0.981
342.357	0.995
343.258	0.901
344.17	0.912
345.075	0.905
345.953	0.878
346.839	0.886
347.708	0.869
348.629	0.921
349.538	0.909
350.449	0.911
351.347	0.898
352.24	0.893
353.078	0.838
353.936	0.858

354.837	0.901
355.747	0.91
356.672	0.925
357.614	0.942

HRV ANALYSIS RESULTS - 02-Mar-2020 12:44:05
Kubios HRV Standard
3.3.1
released in August 2019

Analyzed by: Participant 7 - -

File name: C:\Users
Measurement date: xx/xx/xx xx:xx:xx
File type: asciiRR
Channel label: RR data
Data length: 00:06:00 (h:min:s)
Measurement rate: -

Parameters
 Number of samples: 1
 Detrending method: Smoothn priors (lambda: 500)
 Min/Max HR as average of: 5 beats
 Threshold for NNxx/pNNxx: 50 ms
 Frequency bands
  VLF: 0 - 0.04 Hz
  LF: 0.04 - 0.15 Hz
  HF: 0.15 - 0.4 Hz
 Interpolation rate: 4 Hz
 Points in frequency-domain: 300 points/Hz
 FFT spectrum options
  Window width: 300 s
  Window overlap: 50 %
 AR spectrum options
  AR model order: 16
  Use factorization: No
 Apply detrending for nonlinear analysis: 1
 Entropy embedding dimension: 2

Entropy	tolerance: 0.2 x SD
DFA	short-term fluctuations: 4-12 beats
DFA	long-term fluctuations: 13-64 beats

RR Interval Samples Selected for Analysis

	Sample 1
Sample limits (hh:mm:ss):	00:01:00-00:06:00
Sample Analysis Type: Single sample	
Artifact correction: none	
Artifacts (%):	-

RESULTS FOR A SINGLE SAMPLE

Results Overview

PNS index:	-1.06
SNS index:	1.4175
Stress index:	16.2225

Time-Domain Results

Statistical parameters

Mean RR (ms):	859.4556
STD RR (ms):	25.9014
Mean HR (beats/min):	69.8116
STD HR (beats/min):	2.1278
Min HR (beats/min):	65.0026
Max HR (beats/min):	80.3772
RMSSD (ms):	16.5744
NNxx (beats):	5
pNNxx (%):	1.4368
SDANN (ms):	
SDNN index (ms):	

Geometric parameters

RR tri index:	7.122449	
TINN (ms):	126	

Frequency-Domain Results	FFT spectrum	AR spectrum
Peak frequencies		
VLF (Hz):	0.036667	0.003333
LF (Hz):	0.11	0.11
HF (Hz):	0.183333	0.15
Absolute powers		
VLF (ms^2):	60.9646	101.9863
LF (ms^2):	629.64	477.5612
HF (ms^2):	78.634	69.9963
VLF (log):	4.1103	4.6248
LF (log):	6.4451	6.1687
HF (log):	4.3648	4.2484
Relative powers		
VLF (%):	7.9251	15.6989
LF (%):	81.85	73.5117
HF (%):	10.222	10.7746
Normalized powers		
LF (n.u.):	88.895	87.2014
HF (n.u.):	11.1018	12.7811
Total power (ms^2):	769.261	649.6394
LF/HF ratio:	8.0072	6.8227

Nonlinear Results	
Poincare plot	
SD1 (ms):	11.736823
SD2 (ms):	34.688077
SD2/SD1 ratio:	2.955491
Approximate entropy (ApEn):	1.0277
Sample entropy (SampEn):	1.3549

Detrended fluctuation analysis (DFA)

alpha 1:	1.4771
alpha 2:	0.4042

RR INTERVAL DATA and SPECTRUM ESTIMATES

SAMPLE 1

RR Data		FFT spectrum		AR Spectrum				
Time (s)	RR interval (s)	Frequency (Hz)	PSD (ms^2/Hz)	Frequency (Hz)	PSD (ms^2/Hz)	VLF comp. (ms^2/Hz)	LF comp. (ms^2/Hz)	HF comp. (ms^2/Hz)
60.792	0.899	0	4.2167	0	1329.3815			
61.672	0.88	0.003	1.3215	0.003	2656.8336			
62.547	0.875	0.007	1.076	0.007	2651.1848			
63.422	0.875	0.01	13.2423	0.01	2642.2251			
64.313	0.891	0.013	78.7472	0.013	2630.6079			
65.207	0.894	0.017	79.4389	0.017	2617.1935			
66.105	0.898	0.02	512.9179	0.02	2603.0056			
66.998	0.893	0.023	517.6577	0.023	2589.1857			
67.869	0.871	0.027	3076.1035	0.027	2576.9527			
68.722	0.853	0.03	4048.04	0.03	2567.5713			
69.565	0.843	0.033	3758.811	0.033	2562.3325			
70.401	0.836	0.037	4149.7102	0.037	2562.546			
71.262	0.861	0.04	4069.2366	0.04	2569.5468			
72.157	0.895	0.043	3776.6656	0.043	2584.7142			
73.07	0.913	0.047	4499.56	0.047	2609.502			
73.983	0.913	0.05	3099.8691	0.05	2645.4812			
74.89	0.907	0.053	107.7402	0.053	2694.3923			
75.775	0.885	0.057	1594.8843	0.057	2758.2101			
76.656	0.881	0.06	2661.0753	0.06	2839.2185			
77.538	0.882	0.063	4636.2171	0.063	2940.0976			
78.422	0.884	0.067	5986.6681	0.067	3064.0196			
79.326	0.904	0.07	1722.9795	0.07	3214.7489			
80.239	0.913	0.073	7073.5836	0.073	3396.7311			

81.151	0.912	0.077	7524.2184	0.077	3615.1424
82.051	0.9	0.08	1628.7174	0.08	3875.8352
82.937	0.886	0.083	3004.611	0.083	4185.0642
83.798	0.861	0.087	2451.6153	0.087	4548.7799
84.666	0.868	0.09	928.9981	0.09	4971.1343
85.527	0.861	0.093	4568.5106	0.093	5451.6766
86.414	0.887	0.097	13250.9407	0.097	5980.6464
87.304	0.89	0.1	12545.675	0.1	6532.2025
88.189	0.885	0.103	8666.7447	0.103	7057.0854
89.073	0.884	0.107	8053.0294	0.107	7479.7413
89.952	0.879	0.11	17343.272	0.11	7708.6447
90.816	0.864	0.113	11184.0914	0.113	7665.7169
91.674	0.858	0.117	8210.0024	0.117	7324.7168
92.526	0.852	0.12	7734.7332	0.12	6730.6392
93.389	0.863	0.123	10051.6749	0.123	5980.8021
94.257	0.868	0.127	14873.3919	0.127	5182.2927
95.135	0.878	0.13	8523.1655	0.13	4417.5361
96.02	0.885	0.133	4885.6004	0.133	3733.2406
96.895	0.875	0.137	4020.0929	0.137	3146.4551
97.775	0.88	0.14	1722.0818	0.14	2655.8572
98.665	0.89	0.143	446.0971	0.143	2251.2691
99.557	0.892	0.147	25.9433	0.147	1919.6385
100.467	0.91	0.15	284.2079	0.15	1648.1265
101.371	0.904	0.153	667.2319	0.153	1425.4049
102.277	0.906	0.157	999.5494	0.157	1242.0068
103.175	0.898	0.16	156.2109	0.16	1090.2462
104.059	0.884	0.163	177.9083	0.163	963.9766
104.937	0.878	0.167	464.4875	0.167	858.3171
105.812	0.875	0.17	83.2976	0.17	769.3996
106.689	0.877	0.173	275.7159	0.173	694.1563
107.556	0.867	0.177	1261.8162	0.177	630.1464
108.416	0.86	0.18	3681.4208	0.18	575.4201
109.265	0.849	0.183	4535.9668	0.183	528.4126

110.118	0.853	0.187	2294.5354	0.187	487.8608
110.976	0.858	0.19	71.6863	0.19	452.7399
111.852	0.876	0.193	38.5249	0.193	422.2135
112.718	0.866	0.197	146.8442	0.197	395.5951
113.546	0.828	0.2	32.4389	0.2	372.3176
114.329	0.783	0.203	88.5519	0.203	351.91
115.123	0.794	0.207	175.9508	0.207	333.9788
115.92	0.797	0.21	148.9291	0.21	318.1927
116.712	0.792	0.213	305.4487	0.213	304.2711
117.501	0.789	0.217	478.6701	0.217	291.9744
118.311	0.81	0.22	231.5709	0.22	281.0965
119.154	0.843	0.223	239.4907	0.223	271.458
120.007	0.853	0.227	159.6005	0.227	262.9016
120.866	0.859	0.23	136.7207	0.23	255.2872
121.72	0.854	0.233	815.6674	0.233	248.4887
122.571	0.851	0.237	44.5672	0.237	242.391
123.432	0.861	0.24	594.1167	0.24	236.8874
124.309	0.877	0.243	264.5474	0.243	231.8779
125.183	0.874	0.247	41.6953	0.247	227.2673
126.047	0.864	0.25	113.9922	0.25	222.9646
126.922	0.875	0.253	172.6999	0.253	218.882
127.819	0.897	0.257	166.6873	0.257	214.9354
128.719	0.9	0.26	59.1974	0.26	211.0442
129.628	0.909	0.263	102.045	0.263	207.1326
130.5	0.872	0.267	21.4038	0.267	203.1308
131.359	0.859	0.27	81.9477	0.27	198.9767
132.247	0.888	0.273	291.2779	0.273	194.6182
133.159	0.912	0.277	89.6658	0.277	190.0146
134.044	0.885	0.28	33.906	0.28	185.1387
134.92	0.876	0.283	72.2282	0.283	179.9779
135.781	0.861	0.287	349.5358	0.287	174.5349
136.646	0.865	0.29	77.3734	0.29	168.8275
137.519	0.873	0.293	23.7302	0.293	162.887

138.397	0.878	0.297	55.4324	0.297	156.7566
139.273	0.876	0.3	42.3943	0.3	150.4883
140.147	0.874	0.303	150.8123	0.303	144.1401
140.999	0.852	0.307	123.1173	0.307	137.7722
141.855	0.856	0.31	19.0502	0.31	131.4444
142.721	0.866	0.313	55.5329	0.313	125.2131
143.614	0.893	0.317	13.1461	0.317	119.1291
144.525	0.911	0.32	183.9476	0.32	113.2364
145.433	0.908	0.323	244.7295	0.323	107.5711
146.326	0.893	0.327	67.4738	0.327	102.1614
147.204	0.878	0.33	29.1845	0.33	97.0278
148.086	0.882	0.333	43.2217	0.333	92.1837
148.976	0.89	0.337	375.2304	0.337	87.6361
149.865	0.889	0.34	501.3874	0.34	83.3868
150.752	0.887	0.343	29.7771	0.343	79.4334
151.616	0.864	0.347	182.3289	0.347	75.7696
152.477	0.861	0.35	72.9134	0.35	72.387
153.348	0.871	0.353	36.051	0.353	69.2752
154.247	0.899	0.357	66.9851	0.357	66.4227
155.145	0.898	0.36	33.7562	0.36	63.8174
156.048	0.903	0.363	170.1371	0.363	61.4468
156.933	0.885	0.367	192.4934	0.367	59.2987
157.813	0.88	0.37	115.6417	0.37	57.3613
158.688	0.875	0.373	7.9129	0.373	55.6232
159.574	0.886	0.377	82.5655	0.377	54.0739
160.443	0.869	0.38	38.7971	0.38	52.7036
161.31	0.867	0.383	95.7994	0.383	51.5031
162.209	0.899	0.387	38.8307	0.387	50.4643
163.083	0.874	0.39	5.079	0.39	49.5797
163.952	0.869	0.393	75.2749	0.393	48.8426
164.842	0.89	0.397	68.1215	0.397	48.2471
165.742	0.9	0.4	3.3366	0.4	47.7878
166.647	0.905	0.403	43.3437	0.403	47.4598

167.54	0.893	0.407	35.5125	0.407	47.2585
168.42	0.88	0.41	11.8331	0.41	47.1795
169.298	0.878	0.413	17.7118	0.413	47.2185
170.185	0.887	0.417	39.4961	0.417	47.3708
171.093	0.908	0.42	62.9011	0.42	47.631
172.011	0.918	0.423	4.5556	0.423	47.9928
172.922	0.911	0.427	16.4331	0.427	48.4484
173.804	0.882	0.43	8.9182	0.43	48.9881
174.683	0.879	0.433	13.8207	0.433	49.5995
175.557	0.874	0.437	14.3966	0.437	50.2672
176.444	0.887	0.44	41.0489	0.44	50.9718
177.336	0.892	0.443	19.4335	0.443	51.6895
178.214	0.878	0.447	39.6759	0.447	52.3918
179.068	0.854	0.45	120.4839	0.45	53.0453
179.917	0.849	0.453	49.8462	0.453	53.6122
180.769	0.852	0.457	125.3099	0.457	54.0511
181.654	0.885	0.46	43.7033	0.46	54.3192
182.55	0.896	0.463	0.9818	0.463	54.3745
183.454	0.904	0.467	9.7971	0.467	54.1793
184.348	0.894	0.47	23.3047	0.47	53.7037
185.243	0.895	0.473	117.3208	0.473	52.9289
186.131	0.888	0.477	122.9716	0.477	51.8504
187.024	0.893	0.48	12.3515	0.48	50.4787
187.92	0.896	0.483	27.8088	0.483	48.8394
188.808	0.888	0.487	18.6244	0.487	46.9707
189.685	0.877	0.49	81.5914	0.49	44.9202
190.539	0.854	0.493	43.5429	0.493	42.7406
191.382	0.843	0.497	28.7158	0.497	40.4852
192.221	0.839	0.5	2.8776	0.5	38.2042
193.067	0.846				
193.927	0.86				
194.796	0.869				
195.676	0.88				

196.546	0.87
197.417	0.871
198.297	0.88
199.187	0.89
200.079	0.892
200.962	0.883
201.828	0.866
202.699	0.871
203.581	0.882
204.462	0.881
205.358	0.896
206.246	0.888
207.122	0.876
207.988	0.866
208.861	0.873
209.727	0.866
210.594	0.867
211.456	0.862
212.326	0.87
213.209	0.883
214.115	0.906
215.028	0.913
215.943	0.915
216.839	0.896
217.719	0.88
218.588	0.869
219.469	0.881
220.349	0.88
221.234	0.885
222.109	0.875
222.953	0.844
223.79	0.837
224.624	0.834

225.467	0.843
226.333	0.866
227.271	0.938
228.237	0.966
229.184	0.947
230.125	0.941
231.041	0.916
231.913	0.872
232.745	0.832
233.561	0.816
234.382	0.821
235.238	0.856
236.17	0.932
237.115	0.945
238.068	0.953
238.999	0.931
239.897	0.898
240.751	0.854
241.582	0.831
242.417	0.835
243.26	0.843
244.146	0.886
245.067	0.921
245.996	0.929
246.911	0.915
247.803	0.892
248.682	0.879
249.523	0.841
250.343	0.82
251.167	0.824
251.988	0.821
252.824	0.836
253.668	0.844

254.507	0.839
255.331	0.824
256.144	0.813
256.926	0.782
257.711	0.785
258.476	0.765
259.242	0.766
260.013	0.771
260.795	0.782
261.581	0.786
262.379	0.798
263.192	0.813
264.02	0.828
264.872	0.852
265.756	0.884
266.653	0.897
267.551	0.898
268.444	0.893
269.324	0.88
270.18	0.856
271.037	0.857
271.893	0.856
272.759	0.866
273.658	0.899
274.569	0.911
275.487	0.918
276.387	0.9
277.285	0.898
278.161	0.876
279.038	0.877
279.916	0.878
280.79	0.874
281.624	0.834

282.482	0.858
283.335	0.853
284.193	0.858
285.016	0.823
285.834	0.818
286.71	0.876
287.536	0.826
288.364	0.828
289.206	0.842
290.068	0.862
290.965	0.897
291.863	0.898
292.766	0.903
293.664	0.898
294.55	0.886
295.432	0.882
296.298	0.866
297.171	0.873
298.042	0.871
298.928	0.886
299.829	0.901
300.729	0.9
301.619	0.89
302.498	0.879
303.358	0.86
304.206	0.848
305.051	0.845
305.902	0.851
306.775	0.873
307.67	0.895
308.567	0.897
309.46	0.893
310.339	0.879

311.192	0.853
312.006	0.814
312.806	0.8
313.608	0.802
314.431	0.823
315.304	0.873
316.199	0.895
317.1	0.901
317.996	0.896
318.886	0.89
319.754	0.868
320.61	0.856
321.468	0.858
322.319	0.851
323.15	0.831
323.963	0.813
324.753	0.79
325.535	0.782
326.316	0.781
327.088	0.772
327.847	0.759
328.592	0.745
329.334	0.742
330.066	0.732
330.803	0.737
331.55	0.747
332.319	0.769
333.101	0.782
333.891	0.79
334.691	0.8
335.482	0.791
336.267	0.785
337.041	0.774

337.816	0.775
338.593	0.777
339.375	0.782
340.167	0.792
340.961	0.794
341.749	0.788
342.534	0.785
343.317	0.783
344.087	0.77
344.852	0.765
345.616	0.764
346.376	0.76
347.138	0.762
347.914	0.776
348.687	0.773
349.458	0.771
350.227	0.769
350.987	0.76
351.724	0.737
352.471	0.747
353.232	0.761
354.006	0.774
354.807	0.801
355.623	0.816
356.456	0.833
357.294	0.838
358.133	0.839
358.979	0.846
359.843	0.864

HRV ANALYSIS RESULTS - 02-Mar-2020 12:45:48
Kubios HRV Standard
3.3.1
released in August 2019

Analyzed by: Participant 8 - -

File name: C:\Users
Measurement date: xx/xx/xx xx:xx:xx
File type: asciiRR
Channel label: RR data
Data length: 00:05:59 (h:min:s)
Measurement rate: -

Parameters
 Number of samples: 1
 Detrending method: Smoothn priors (lambda: 500)
 Min/Max HR as average of: 5 beats
 Threshold for NNxx/pNNxx: 50 ms
 Frequency bands
  VLF: 0 - 0.04 Hz
  LF: 0.04 - 0.15 Hz
  HF: 0.15 - 0.4 Hz
 Interpolation rate: 4 Hz
 Points in frequency-domain: 300 points/Hz
 FFT spectrum options
  Window width: 300 s
  Window overlap: 50 %
 AR spectrum options
  AR model order: 16
  Use factorization: No
 Apply detrending for nonlinear analysis: 1
 Entropy embedding dimension: 2

Entropy	tolerance: 0.2 x SD
DFA	short-term fluctuations: 4-12 beats
DFA	long-term fluctuations: 13-64 beats

RR Interval Samples Selected for Analysis

	Sample 1
Sample limits (hh:mm:ss):	00:00:59-00:05:59
Sample Analysis Type: Single sample	
Artifact correction: none	
Artifacts (%):	-

RESULTS FOR A SINGLE SAMPLE

Results Overview

PNS index:	-0.3258
SNS index:	1.321
Stress index:	20.0061

Time-Domain Results

Statistical parameters

Mean RR (ms):	971.1327
STD RR (ms):	17.3499
Mean HR (beats/min):	61.7835
STD HR (beats/min):	1.1103
Min HR (beats/min):	58.3862
Max HR (beats/min):	65.1268
RMSSD (ms):	18.8668
NNxx (beats):	0
pNNxx (%):	0
SDANN (ms):	
SDNN index (ms):	

Geometric parameters

RR tri index:	5.618182	
TINN (ms):	97	
Frequency-Domain Results	FFT spectrum	AR spectrum
Peak frequencies		
VLF (Hz):	0.033333	0.04
LF (Hz):	0.046667	0.073333
HF (Hz):	0.316667	0.336667
Absolute powers		
VLF (ms^2):	12.8912	25.4868
LF (ms^2):	144.2264	142.2071
HF (ms^2):	97.77	108.0117
VLF (log):	2.5565	3.2382
LF (log):	4.9714	4.9573
HF (log):	4.5826	4.6822
Relative powers		
VLF (%):	5.0482	9.2403
LF (%):	56.4792	51.5575
HF (%):	38.2869	39.1599
Normalized powers		
LF (n.u.):	59.482	56.8066
HF (n.u.):	40.3224	43.1468
Total power (ms^2):	255.3618	275.8226
LF/HF ratio:	1.4752	1.3166
Nonlinear Results		
Poincare plot		
SD1 (ms):	13.362734	
SD2 (ms):	20.59193	
SD2/SD1 ratio:	1.540997	
Approximate entropy (ApE	1.1459	
Sample entropy (SampEn)	2.0006	

Detrended fluctuation analysis (DFA)

alpha 1:	0.9711
alpha 2:	0.2984

RR INTERVAL DATA and SPECTRUM ESTIMATES

SAMPLE 1								
RR Data		FFT spectrum		AR Spectrum				
Time (s)	RR interval (s)	Frequency (Hz)	PSD (ms^2/Hz)	Frequency (Hz)	PSD (ms^2/Hz)	VLF comp. (ms^2/Hz)	LF comp. (ms^2/Hz)	HF comp. (ms^2/Hz)
59.405	1.02	0	8.51	0	258.7235			
60.441	1.036	0.003	4.2248	0.003	519.5783			
61.461	1.02	0.007	0.3589	0.007	526.038			
62.5	1.039	0.01	6.7229	0.01	537.0278			
63.525	1.025	0.013	32.6957	0.013	552.8972			
64.528	1.003	0.017	55.8067	0.017	574.1644			
65.528	1	0.02	185.7877	0.02	601.5484			
66.501	0.973	0.023	712.3189	0.023	636.0134			
67.471	0.97	0.027	153.0549	0.027	678.831			
68.443	0.972	0.03	384.709	0.03	731.6615			
69.424	0.981	0.033	1375.807	0.033	796.66			
70.381	0.957	0.037	818.2699	0.037	876.606			
71.356	0.975	0.04	300.4787	0.04	975.0507			
72.324	0.968	0.043	4386.8822	0.043	1096.4484			
73.275	0.951	0.047	6109.7868	0.047	1246.1901			
74.239	0.964	0.05	3387.6155	0.05	1430.3346			
75.209	0.97	0.053	1080.6569	0.053	1654.6131			
76.162	0.953	0.057	2704.6529	0.057	1921.913			
77.137	0.975	0.06	3395.8561	0.06	2227.1302			
78.116	0.979	0.063	828.6297	0.063	2548.8844			
79.078	0.962	0.067	122.057	0.067	2841.2278			
80.028	0.95	0.07	2489.5716	0.07	3036.1051			
80.973	0.945	0.073	2802.8777	0.073	3069.5972			

81.904	0.931	0.077	32.9534	0.077	2922.823
82.842	0.938	0.08	1860.7703	0.08	2637.6771
83.821	0.979	0.083	2807.2685	0.083	2287.7248
84.782	0.961	0.087	1641.241	0.087	1937.2475
85.737	0.955	0.09	9.1062	0.09	1623.0109
86.707	0.97	0.093	1422.4186	0.093	1357.9287
87.662	0.955	0.097	2442.9357	0.097	1141.2868
88.593	0.931	0.1	1058.7364	0.1	966.7479
89.533	0.94	0.103	189.2934	0.103	826.7092
90.471	0.938	0.107	521.0409	0.107	714.1651
91.393	0.922	0.11	166.6521	0.11	623.2822
92.337	0.944	0.113	139.721	0.113	549.4243
93.288	0.951	0.117	132.4535	0.117	488.9847
94.224	0.936	0.12	612.341	0.12	439.1817
95.183	0.959	0.123	218.9679	0.123	397.873
96.168	0.985	0.127	150.5239	0.127	363.4045
97.133	0.965	0.13	40.8963	0.13	334.4928
98.073	0.94	0.133	298.7677	0.133	310.1366
99.041	0.968	0.137	507.3815	0.137	289.5489
100.012	0.971	0.14	721.4826	0.14	272.108
100.963	0.951	0.143	362.3726	0.143	257.3188
101.916	0.953	0.147	211.0179	0.147	244.7852
102.888	0.972	0.15	537.3056	0.15	234.1886
103.834	0.946	0.153	405.4242	0.153	225.2712
104.772	0.938	0.157	402.9042	0.157	217.8239
105.72	0.948	0.16	202.7825	0.16	211.6764
106.645	0.925	0.163	269.2184	0.163	206.6896
107.565	0.92	0.167	385.5226	0.167	202.7501
108.49	0.925	0.17	9.9122	0.17	199.7647
109.418	0.928	0.173	384.5074	0.173	197.6575
110.326	0.908	0.177	343.9936	0.177	196.3661
111.252	0.926	0.18	126.154	0.18	195.8391
112.178	0.926	0.183	58.4118	0.183	196.0344

113.089	0.911	0.187	46.7434	0.187	196.9164
114.025	0.936	0.19	107.0354	0.19	198.4551
114.976	0.951	0.193	52.5438	0.193	200.6236
115.913	0.937	0.197	180.182	0.197	203.3973
116.866	0.953	0.2	131.3517	0.2	206.7519
117.83	0.964	0.203	16.1342	0.203	210.6621
118.76	0.93	0.207	0.8409	0.207	215.1002
119.699	0.939	0.21	34.426	0.21	220.0347
120.649	0.95	0.213	3.9495	0.213	225.4295
121.562	0.913	0.217	242.3411	0.217	231.243
122.48	0.918	0.22	156.0201	0.22	237.4278
123.412	0.932	0.223	3.5967	0.223	243.9308
124.327	0.915	0.227	18.4852	0.227	250.6945
125.272	0.945	0.23	1.1362	0.23	257.6588
126.246	0.974	0.233	48.2998	0.233	264.7636
127.182	0.936	0.237	105.7789	0.237	271.9525
128.142	0.96	0.24	124.1364	0.24	279.1773
129.101	0.959	0.243	191.8938	0.243	286.4025
130.044	0.943	0.247	328.4354	0.247	293.6109
130.975	0.931	0.25	170.4527	0.25	300.808
131.94	0.965	0.253	11.0085	0.253	308.0266
132.883	0.943	0.257	34.6566	0.257	315.3304
133.837	0.954	0.26	80.8071	0.26	322.8174
134.813	0.976	0.263	102.6656	0.263	330.6218
135.775	0.962	0.267	319.8686	0.267	338.9167
136.745	0.97	0.27	195.5653	0.27	347.9168
137.729	0.984	0.273	163.9664	0.273	357.8824
138.695	0.966	0.277	321.3562	0.277	369.1251
139.655	0.96	0.28	48.8964	0.28	382.0175
140.64	0.985	0.283	285.3879	0.283	397.0059
141.612	0.972	0.287	1195.589	0.287	414.6303
142.574	0.962	0.29	1041.7866	0.29	435.5507
143.563	0.989	0.293	310.4135	0.293	460.5835

144.549	0.986	0.297	744.5245	0.297	490.7506
145.517	0.968	0.3	476.4332	0.3	527.3415
146.492	0.975	0.303	118.1255	0.303	571.9909
147.471	0.979	0.307	771.7788	0.307	626.7638
148.427	0.956	0.31	1360.3933	0.31	694.2241
149.409	0.982	0.313	3254.633	0.313	777.421
150.394	0.985	0.317	3613.3692	0.317	879.6244
151.357	0.963	0.32	1332.7929	0.32	1003.4469
152.34	0.983	0.323	6.5926	0.323	1148.6566
153.327	0.987	0.327	468.8345	0.327	1307.772
154.316	0.989	0.33	575.0607	0.33	1459.5477
155.301	0.985	0.333	165.2048	0.333	1565.1511
156.285	0.984	0.337	1528.4781	0.337	1578.988
157.221	0.936	0.34	2236.9733	0.34	1479.1568
158.161	0.94	0.343	1109.4892	0.343	1290.1053
159.082	0.921	0.347	654.8615	0.347	1065.3487
159.97	0.888	0.35	92.4099	0.35	851.2087
160.888	0.918	0.353	133.0176	0.353	670.4326
161.821	0.933	0.357	145.6221	0.357	527.2258
162.757	0.936	0.36	161.7663	0.36	417.097
163.705	0.948	0.363	515.0724	0.363	333.2619
164.673	0.968	0.367	206.5992	0.367	269.4017
165.628	0.955	0.37	114.2037	0.37	220.4466
166.6	0.972	0.373	79.7203	0.373	182.5721
167.596	0.996	0.377	17.8341	0.377	152.9659
168.564	0.968	0.38	113.5798	0.38	129.5768
169.534	0.97	0.383	95.0089	0.383	110.9076
170.511	0.977	0.387	77.0008	0.387	95.8588
171.46	0.949	0.39	38.8634	0.39	83.6168
172.413	0.953	0.393	33.3159	0.393	73.5739
173.389	0.976	0.397	113.0713	0.397	65.2714
174.339	0.95	0.4	255.1446	0.4	58.36
175.284	0.945	0.403	156.524	0.403	52.5713

176.259	0.975	0.407	41.7657	0.407	47.697
177.207	0.948	0.41	42.6911	0.41	43.574
178.156	0.949	0.413	123.1413	0.413	40.0741
179.121	0.965	0.417	97.6789	0.417	37.0952
180.058	0.937	0.42	68.7577	0.42	34.5562
181.008	0.95	0.423	82.3589	0.423	32.3918
181.96	0.952	0.427	31.5992	0.427	30.5498
182.9	0.94	0.43	93.3746	0.43	28.988
183.866	0.966	0.433	97.4646	0.433	27.6725
184.866	1	0.437	41.0793	0.437	26.5764
185.856	0.99	0.44	3.2141	0.44	25.6784
186.832	0.976	0.443	52.2769	0.443	24.9621
187.839	1.007	0.447	222.1243	0.447	24.4152
188.844	1.005	0.45	76.1331	0.45	24.0294
189.83	0.986	0.453	26.3436	0.453	23.7997
190.84	1.01	0.457	142.9476	0.457	23.7247
191.852	1.012	0.46	96.261	0.46	23.8059
192.838	0.986	0.463	25.4677	0.463	24.0485
193.809	0.971	0.467	103.8794	0.467	24.4607
194.807	0.998	0.47	106.0777	0.47	25.0543
195.804	0.997	0.473	23.9226	0.473	25.8443
196.779	0.975	0.477	34.8024	0.477	26.8487
197.775	0.996	0.48	22.11	0.48	28.0875
198.771	0.996	0.483	41.8353	0.483	29.5805
199.742	0.971	0.487	19.3523	0.487	31.3435
200.728	0.986	0.49	26.7266	0.49	33.3803
201.712	0.984	0.493	59.9211	0.493	35.6704
202.684	0.972	0.497	2.7731	0.497	38.1491
203.653	0.969	0.5	10.1793	0.5	40.6821
204.639	0.986				
205.631	0.992				
206.599	0.968				
207.581	0.982				

208.572	0.991
209.542	0.97
210.534	0.992
211.528	0.994
212.505	0.977
213.496	0.991
214.473	0.977
215.436	0.963
216.411	0.975
217.391	0.98
218.358	0.967
219.321	0.963
220.299	0.978
221.268	0.969
222.241	0.973
223.224	0.983
224.185	0.961
225.148	0.963
226.134	0.986
227.116	0.982
228.086	0.97
229.078	0.992
230.068	0.99
231.031	0.963
231.977	0.946
232.957	0.98
233.925	0.968
234.901	0.976
235.905	1.004
236.916	1.011
237.885	0.969
238.86	0.975
239.825	0.965

240.765	0.94
241.728	0.963
242.657	0.929
243.591	0.934
244.493	0.902
245.412	0.919
246.354	0.942
247.275	0.921
248.22	0.945
249.188	0.968
250.159	0.971
251.113	0.954
252.091	0.978
253.07	0.979
254.023	0.953
254.999	0.976
255.977	0.978
256.933	0.956
257.906	0.973
258.886	0.98
259.847	0.961
260.822	0.975
261.812	0.99
262.795	0.983
263.759	0.964
264.743	0.984
265.717	0.974
266.665	0.948
267.649	0.984
268.648	0.999
269.618	0.97
270.61	0.992
271.6	0.99

272.565	0.965
273.526	0.961
274.518	0.992
275.494	0.976
276.472	0.978
277.479	1.007
278.483	1.004
279.463	0.98
280.457	0.994
281.466	1.009
282.483	1.017
283.475	0.992
284.454	0.979
285.443	0.989
286.448	1.005
287.423	0.975
288.422	0.999
289.424	1.002
290.411	0.987
291.396	0.985
292.402	1.006
293.39	0.988
294.368	0.978
295.371	1.003
296.373	1.002
297.333	0.96
298.3	0.967
299.272	0.972
300.234	0.962
301.191	0.957
302.177	0.986
303.154	0.977
304.123	0.969

305.119	0.996
306.121	1.002
307.097	0.976
308.086	0.989
309.072	0.986
310.047	0.975
311.053	1.006
312.057	1.004
313.037	0.98
314.02	0.983
315.023	1.003
316.001	0.978
316.993	0.992
318.008	1.015
319.004	0.996
320	0.996
320.998	0.998
321.973	0.975
322.965	0.992
323.986	1.021
324.982	0.996
325.982	1
326.993	1.011
327.966	0.973
328.904	0.938
329.829	0.925
330.759	0.93
331.688	0.929
332.646	0.958
333.597	0.951
334.503	0.906
335.429	0.926
336.367	0.938

337.343	0.976
338.338	0.995
339.318	0.98
340.289	0.971
341.285	0.996
342.254	0.969
343.248	0.994
344.236	0.988
345.219	0.983
346.224	1.005
347.244	1.02
348.239	0.995
349.269	1.03
350.326	1.057
351.345	1.019
352.373	1.028
353.412	1.039
354.424	1.012
355.439	1.015
356.455	1.016
357.453	0.998
358.465	1.012

S1: The R code

1. # Jags-MultivariateNormal.R

```
# John Kruschke, November 2015 - June 2017.
# For further info, see:
# Kruschke, J. K. (2015). Doing Bayesian Data Analysis, Second Edition:
# A Tutorial with R, JAGS, and Stan. Academic Press / Elsevier.



# Load the data:
#----------------------------------------------------------------------
myData = read.csv("HRV.csv") # must have file in curr. work. dir.
# y must have named columns, with no missing values!
y = myData[,c("MeanHR","SD1",”SD2” ETC…)]

#----------------------------------------------------------------------
# The rest can remain unchanged, except for the specification of difference of
# correlations at the very end.
#----------------------------------------------------------------------

# Load some functions used below:
source("DBDA2E-utilities.R") # Must be in R's current working directory.
# Install the ellipse package if not already:
want = c("ellipse")
have = want %in% rownames(installed.packages())
if ( any(!have) ) { install.packages( want[!have] ) }

# Standardize the data:
sdOrig = apply(y,2,sd)
meanOrig = apply(y,2,mean)
zy = apply(y,2,function(yVec){(yVec-mean(yVec))/sd(yVec)})
# Assemble data for sending to JAGS:
dataList = list(
  zy = zy ,
  Ntotal =  nrow(zy) ,
  Nvar = ncol(zy) ,
  # Include original data info for transforming to original scale:
  sdOrig = sdOrig ,
  meanOrig = meanOrig ,
  # For wishart (dwish) prior on inverse covariance matrix:
  zRscal = ncol(zy) ,  # for dwish prior
  zRmat = diag(x=1,nrow=ncol(zy))  # Rmat = diag(apply(y,2,var))
)

# Define the model:
modelString = "
model {
```

```
  for ( i in 1:Ntotal ) {
    zy[i,1:Nvar] ~ dmnorm( zMu[1:Nvar] , zInvCovMat[1:Nvar,1:Nvar] )
  }
  for ( varIdx in 1:Nvar ) { zMu[varIdx] ~ dnorm( 0 , 1/2^2 ) }
  zInvCovMat ~ dwish( zRmat[1:Nvar,1:Nvar] , zRscal )
  # Convert invCovMat to sd and correlation:
  zCovMat <- inverse( zInvCovMat )
  for ( varIdx in 1:Nvar ) { zSigma[varIdx] <- sqrt(zCovMat[varIdx,varIdx]) }
  for ( varIdx1 in 1:Nvar ) { for ( varIdx2 in 1:Nvar ) {
    zRho[varIdx1,varIdx2] <- ( zCovMat[varIdx1,varIdx2]
                               / (zSigma[varIdx1]*zSigma[varIdx2]) )
  } }
  # Convert to original scale:
  for ( varIdx in 1:Nvar ) {
    sigma[varIdx] <- zSigma[varIdx] * sdOrig[varIdx]
    mu[varIdx] <- zMu[varIdx] * sdOrig[varIdx] + meanOrig[varIdx]
  }
  for ( varIdx1 in 1:Nvar ) { for ( varIdx2 in 1:Nvar ) {
    rho[varIdx1,varIdx2] <- zRho[varIdx1,varIdx2]
  } }
}
" # close quote for modelString
writeLines( modelString , con="Jags-MultivariateNormal-model.txt" )

# Run the chains:
nChain = 3
nAdapt = 500
nBurnIn = 500
nThin = 10
nStepToSave = 20000
require(rjags)
jagsModel = jags.model( file="Jags-MultivariateNormal-model.txt" ,
                        data=dataList , n.chains=nChain , n.adapt=nAdapt )
update( jagsModel , n.iter=nBurnIn )
codaSamples = coda.samples( jagsModel ,
                            variable.names=c("mu","sigma","rho") ,
                            n.iter=nStepToSave/nChain*nThin , thin=nThin )

# Convergence diagnostics:
parameterNames = varnames(codaSamples) # get all parameter names
for ( parName in parameterNames ) {
  diagMCMC( codaObject=codaSamples , parName=parName )
}

# Examine the posterior distribution:
mcmcMat = as.matrix(codaSamples)
chainLength = nrow(mcmcMat)
Nvar = ncol(y)
```

```
# Create subsequence of steps through chain for plotting:
stepVec = floor(seq(1,chainLength,length=20))

# Make plots of posterior distribution:

# Preparation -- define useful functions:
library(ellipse)
expandRange = function( x , exMult=0.2 ) {
  lowVal = min(x)
  highVal = max(x)
  wid = max(x)-min(x)
  return( c( lowVal - exMult*wid , highVal + exMult*wid ) )
}

for ( varIdx in 1:Nvar ) {
  openGraph(width=7,height=3.5)
  par( mar=c(3.5,3,2,1) , mgp=c(2.0,0.7,0) )
  layout(matrix(1:2,nrow=1))
  # Marginal posterior on means:
  plotPost( mcmcMat[ , paste0("mu[",varIdx,"]") ] ,
            xlab=paste0("mu[",varIdx,"]") ,
            main=paste( "Mean of" , colnames(y)[varIdx] ) )
  # Marginal posterior on standard deviations:
  plotPost( mcmcMat[ , paste0("sigma[",varIdx,"]") ] ,
            xlab=paste0("sigma[",varIdx,"]") ,
            main=paste( "SD of" , colnames(y)[varIdx] ) )
}

for ( varIdx1 in 1:(Nvar-1) ) {
  for ( varIdx2 in (varIdx1+1):Nvar ) {
    openGraph(width=7,height=3.5)
    par( mar=c(3.5,3,2,1) , mgp=c(2.0,0.7,0) )
    layout(matrix(1:2,nrow=1))
    # Marginal posterior on correlation coefficient
    plotPost( mcmcMat[ , paste0("rho[",varIdx1,",",varIdx2,"]") ] ,
              xlab=paste0("rho[",varIdx1,",",varIdx2,"]") ,
              main=paste( "Corr. of" , colnames(y)[varIdx1] ,
                          "and" , colnames(y)[varIdx2] ) )
    # Data with posterior ellipse
    ellipseLevel = 0.90
    plot( y[,c(varIdx1,varIdx2)] , # pch=19 ,
          xlim=expandRange(y[,varIdx1]) , ylim=expandRange(y[,varIdx2]) ,
          xlab=colnames(y)[varIdx1] , ylab=colnames(y)[varIdx2] ,
          main=bquote("Data with posterior "*.(ellipseLevel)*" level contour") )
    # Posterior ellipses:
    for ( stepIdx in stepVec ) {
      points( ellipse( mcmcMat[ stepIdx ,
                          paste0("rho[",varIdx1,",",varIdx2,"]") ] ,
```

```
                    scale=mcmcMat[ stepIdx ,
                                   c( paste0("sigma[",varIdx1,"]") ,
                                      paste0("sigma[",varIdx2,"]") ) ] ,
                    centre=mcmcMat[ stepIdx ,
                                    c( paste0("mu[",varIdx1,"]") ,
                                       paste0("mu[",varIdx2,"]") ) ] ,
                    level=ellipseLevel ) ,
            type="l" , col="skyblue" , lwd=1 )
    }
    # replot data:
    points( y[,c(varIdx1,varIdx2)] )
  }
}

# Show data descriptives on console:
cor( y )
apply(y,2,mean)
apply(y,2,sd)
```

2. DBDA2E-utilities.R

```
# Utility programs for use with the book,
#   Kruschke, J. K. (2015). Doing Bayesian Data Analysis, Second Edition:
#   A Tutorial with R, JAGS, and Stan. Academic Press / Elsevier.
# This file contains several functions that are called by other programs
# or can be called directly by the user. To load all the functions into
# R's working memory, at R's command line type:
# source("DBDA2E-utilities.R")

#------------------------------------------------------------------------------

bookInfo = "Kruschke, J. K. (2015). Doing Bayesian Data Analysis, Second Edition:\nA Tutorial with R, JAGS, and Stan. Academic Press / Elsevier."
bannerBreak = "\n**********************************************************************\n"
cat(paste0(bannerBreak,bookInfo,bannerBreak,"\n"))

#------------------------------------------------------------------------------
# Check that required packages are installed:
want = c("parallel","rjags","runjags","compute.es")
have = want %in% rownames(installed.packages())
if ( any(!have) ) { install.packages( want[!have] ) }

# Load rjags. Assumes JAGS is already installed.
try( library(rjags) )
# Load runjags. Assumes JAGS is already installed.
try( library(runjags) )
try( runjags.options( inits.warning=FALSE , rng.warning=FALSE ) )

# set default number of chains and parallelness for MCMC:
library(parallel) # for detectCores().
nCores = detectCores()
if ( !is.finite(nCores) ) { nCores = 1 }
if ( nCores > 4 ) {
  nChainsDefault = 4  # because JAGS has only 4 rng's.
  runjagsMethodDefault = "parallel"
}
if ( nCores == 4 ) {
  nChainsDefault = 3  # save 1 core for other processes.
  runjagsMethodDefault = "parallel"
}
if ( nCores < 4 ) {
  nChainsDefault = 3
  runjagsMethodDefault = "rjags" # NOT parallel
}
```

```
#------------------------------------------------------------------------------
# Functions for opening and saving graphics that operate the same for
# Windows and Macintosh and Linux operating systems. At least, that's the hope!

openGraph = function( width=7 , height=7 , mag=1.0 , ... ) {
  if ( .Platform$OS.type != "windows" ) { # Mac OS, Linux
    tryInfo = try( X11( width=width*mag , height=height*mag , type="cairo" ,
                        ... ) )
    if ( class(tryInfo)=="try-error" ) {
      lineInput = readline("WARNING: Previous graphics windows will be closed because of too many open
windows.\nTO CONTINUE, PRESS <ENTER> IN R CONSOLE.\n")
      graphics.off()
      X11( width=width*mag , height=height*mag , type="cairo" , ... )
    }
  } else { # Windows OS
    tryInfo = try( windows( width=width*mag , height=height*mag , ... ) )
    if ( class(tryInfo)=="try-error" ) {
      lineInput = readline("WARNING: Previous graphics windows will be closed because of too many open
windows.\nTO CONTINUE, PRESS <ENTER> IN R CONSOLE.\n")
      graphics.off()
      windows( width=width*mag , height=height*mag , ... )
    }
  }
}

saveGraph = function( file="saveGraphOutput" , type="pdf" , ... ) {
  if ( .Platform$OS.type != "windows" ) { # Mac OS, Linux
    if ( any( type == c("png","jpeg","jpg","tiff","bmp")) ) {
      sptype = type
      if ( type == "jpg" ) { sptype = "jpeg" }
      savePlot( file=paste0(file,".",type) , type=sptype , ... )
    }
    if ( type == "pdf" ) {
      dev.copy2pdf(file=paste0(file,".",type) , ... )
    }
    if ( type == "eps" ) {
      dev.copy2eps(file=paste0(file,".",type) , ... )
    }
  } else { # Windows OS
    file=paste0(file,".",type)
    savePlot( file=file , type=type , ... )
  }
}

#------------------------------------------------------------------------------
# Functions for computing limits of HDI's:
```

```
HDIofMCMC = function( sampleVec , credMass=0.95 ) {
  # Computes highest density interval from a sample of representative values,
  #   estimated as shortest credible interval.
  # Arguments:
  #   sampleVec
  #     is a vector of representative values from a probability distribution.
  #   credMass
  #     is a scalar between 0 and 1, indicating the mass within the credible
  #     interval that is to be estimated.
  # Value:
  #   HDIlim is a vector containing the limits of the HDI
  sortedPts = sort( sampleVec )
  ciIdxInc = ceiling( credMass * length( sortedPts ) )
  nCIs = length( sortedPts ) - ciIdxInc
  ciWidth = rep( 0 , nCIs )
  for ( i in 1:nCIs ) {
    ciWidth[ i ] = sortedPts[ i + ciIdxInc ] - sortedPts[ i ]
  }
  HDImin = sortedPts[ which.min( ciWidth ) ]
  HDImax = sortedPts[ which.min( ciWidth ) + ciIdxInc ]
  HDIlim = c( HDImin , HDImax )
  return( HDIlim )
}

HDIofICDF = function( ICDFname , credMass=0.95 , tol=1e-8 , ... ) {
  # Arguments:
  #   ICDFname is R's name for the inverse cumulative density function
  #     of the distribution.
  #   credMass is the desired mass of the HDI region.
  #   tol is passed to R's optimize function.
  # Return value:
  #   Highest density iterval (HDI) limits in a vector.
  # Example of use: For determining HDI of a beta(30,12) distribution, type
  #   HDIofICDF( qbeta , shape1 = 30 , shape2 = 12 )
  #   Notice that the parameters of the ICDFname must be explicitly named;
  #   e.g., HDIofICDF( qbeta , 30 , 12 ) does not work.
  # Adapted and corrected from Greg Snow's TeachingDemos package.
  incredMass =  1.0 - credMass
  intervalWidth = function( lowTailPr , ICDFname , credMass , ... ) {
    ICDFname( credMass + lowTailPr , ... ) - ICDFname( lowTailPr , ... )
  }
  optInfo = optimize( intervalWidth , c( 0 , incredMass ) , ICDFname=ICDFname ,
                      credMass=credMass , tol=tol , ... )
  HDIlowTailPr = optInfo$minimum
  return( c( ICDFname( HDIlowTailPr , ... ) ,
             ICDFname( credMass + HDIlowTailPr , ... ) ) )
}
```

```
HDIofGrid = function( probMassVec , credMass=0.95 ) {
  # Arguments:
  #   probMassVec is a vector of probability masses at each grid point.
  #   credMass is the desired mass of the HDI region.
  # Return value:
  #   A list with components:
  #   indices is a vector of indices that are in the HDI
  #   mass is the total mass of the included indices
  #   height is the smallest component probability mass in the HDI
  # Example of use: For determining HDI of a beta(30,12) distribution
  #   approximated on a grid:
  #   > probDensityVec = dbeta( seq(0,1,length=201) , 30 , 12 )
  #   > probMassVec = probDensityVec / sum( probDensityVec )
  #   > HDIinfo = HDIofGrid( probMassVec )
  #   > show( HDIinfo )
  sortedProbMass = sort( probMassVec , decreasing=TRUE )
  HDIheightIdx = min( which( cumsum( sortedProbMass ) >= credMass ) )
  HDIheight = sortedProbMass[ HDIheightIdx ]
  HDImass = sum( probMassVec[ probMassVec >= HDIheight ] )
  return( list( indices = which( probMassVec >= HDIheight ) ,
               mass = HDImass , height = HDIheight ) )
}

#------------------------------------------------------------------------------
# Function(s) for plotting properties of mcmc coda objects.

DbdaAcfPlot = function( codaObject , parName=varnames(codaObject)[1] , plColors=NULL ) {
  if ( all( parName != varnames(codaObject) ) ) {
    stop("parName must be a column name of coda object")
  }
  nChain = length(codaObject)
  if ( is.null(plColors) ) plColors=1:nChain
  xMat = NULL
  yMat = NULL
  for ( cIdx in 1:nChain ) {
    acfInfo = acf(codaObject[,c(parName)][[cIdx]],plot=FALSE)
    xMat = cbind(xMat,acfInfo$lag)
    yMat = cbind(yMat,acfInfo$acf)
  }
  matplot( xMat , yMat , type="o" , pch=20 , col=plColors , ylim=c(0,1) ,
           main="" , xlab="Lag" , ylab="Autocorrelation" )
  abline(h=0,lty="dashed")
  EffChnLngth = effectiveSize(codaObject[,c(parName)])
  text( x=max(xMat) , y=max(yMat) , adj=c(1.0,1.0) , cex=1.25 ,
        labels=paste("ESS =",round(EffChnLngth,1)) )
}

DbdaDensPlot = function( codaObject , parName=varnames(codaObject)[1] , plColors=NULL ) {
```

```
  if ( all( parName != varnames(codaObject) ) ) {
    stop("parName must be a column name of coda object")
  }
  nChain = length(codaObject) # or nchain(codaObject)
  if ( is.null(plColors) ) plColors=1:nChain
  xMat = NULL
  yMat = NULL
  hdiLims = NULL
  for ( cIdx in 1:nChain ) {
    densInfo = density(codaObject[,c(parName)][[cIdx]])
    xMat = cbind(xMat,densInfo$x)
    yMat = cbind(yMat,densInfo$y)
    hdiLims = cbind(hdiLims,HDIofMCMC(codaObject[,c(parName)][[cIdx]]))
  }
  matplot( xMat , yMat , type="l" , col=plColors ,
           main="" , xlab="Param. Value" , ylab="Density" )
  abline(h=0)
  points( hdiLims[1,] , rep(0,nChain) , col=plColors , pch="|" )
  points( hdiLims[2,] , rep(0,nChain) , col=plColors , pch="|" )
  text( mean(hdiLims) , 0 , "95% HDI" , adj=c(0.5,-0.2) )
  EffChnLngth = effectiveSize(codaObject[,c(parName)])
  MCSE = sd(as.matrix(codaObject[,c(parName)]))/sqrt(EffChnLngth)
  text( max(xMat) , max(yMat) , adj=c(1.0,1.0) , cex=1.25 ,
        paste("MCSE =\n",signif(MCSE,3)) )
}

diagMCMC = function( codaObject , parName=varnames(codaObject)[1] ,
                     saveName=NULL , saveType="jpg" ) {
  DBDAplColors = c("skyblue","black","royalblue","steelblue")
  openGraph(height=5,width=7)
  par( mar=0.5+c(3,4,1,0) , oma=0.1+c(0,0,2,0) , mgp=c(2.25,0.7,0) ,
       cex.lab=1.5 )
  layout(matrix(1:4,nrow=2))
  # traceplot and gelman.plot are from CODA package:
  require(coda)
  coda::traceplot( codaObject[,c(parName)] , main="" , ylab="Param. Value" ,
                   col=DBDAplColors )
  tryVal = try(
    coda::gelman.plot( codaObject[,c(parName)] , main="" , auto.layout=FALSE ,
                       col=DBDAplColors )
  )
  # if it runs, gelman.plot returns a list with finite shrink values:
  if ( class(tryVal)=="try-error" ) {
    plot.new()
    print(paste0("Warning: coda::gelman.plot fails for ",parName))
  } else {
    if ( class(tryVal)=="list" & !is.finite(tryVal$shrink[1]) ) {
      plot.new()
```

```
      print(paste0("Warning: coda::gelman.plot fails for ",parName))
    }
  }
  DbdaAcfPlot(codaObject,parName,plColors=DBDAplColors)
  DbdaDensPlot(codaObject,parName,plColors=DBDAplColors)
  mtext( text=parName , outer=TRUE , adj=c(0.5,0.5) , cex=2.0 )
  if ( !is.null(saveName) ) {
    saveGraph( file=paste0(saveName,"Diag",parName), type=saveType)
  }
}

diagStanFit = function( stanFit , parName ,
                        saveName=NULL , saveType="jpg" ) {
  codaFit = mcmc.list( lapply( 1:ncol(stanFit) ,
                               function(x) { mcmc(as.array(stanFit)[,x,]) } ) )
  DBDAplColors = c("skyblue","black","royalblue","steelblue")
  openGraph(height=5,width=7)
  par( mar=0.5+c(3,4,1,0) , oma=0.1+c(0,0,2,0) , mgp=c(2.25,0.7,0) , cex.lab=1.5 )
  layout(matrix(1:4,nrow=2))
  # traceplot is from rstan package
  require(rstan)
  traceplot(stanFit,pars=parName,nrow=1,ncol=1)#,main="",ylab="Param. Value",col=DBDAplColors)
  # gelman.plot are from CODA package:
  require(coda)
  tryVal = try(
    coda::gelman.plot( codaObject[,c(parName)] , main="" , auto.layout=FALSE ,
                       col=DBDAplColors )
  )
  # if it runs, gelman.plot returns a list with finite shrink values:
  if ( class(tryVal)=="try-error" ) {
    plot.new()
    print(paste0("Warning: coda::gelman.plot fails for ",parName))
  } else {
    if ( class(tryVal)=="list" & !is.finite(tryVal$shrink[1]) ) {
      plot.new()
      print(paste0("Warning: coda::gelman.plot fails for ",parName))
    }
  }
  DbdaAcfPlot(codaFit,parName,plColors=DBDAplColors)
  DbdaDensPlot(codaFit,parName,plColors=DBDAplColors)
  mtext( text=parName , outer=TRUE , adj=c(0.5,0.5) , cex=2.0 )
  if ( !is.null(saveName) ) {
    saveGraph( file=paste0(saveName,"Diag",parName), type=saveType)
  }
}

#------------------------------------------------------------------------------
# Functions for summarizing and plotting distribution of a large sample;
```

```
# typically applied to MCMC posterior.

normalize = function( v ){ return( v / sum(v) ) }

require(coda) # loaded by rjags, but redundancy doesn't hurt

summarizePost = function( paramSampleVec ,
                         compVal=NULL , ROPE=NULL , credMass=0.95 ) {
  meanParam = mean( paramSampleVec )
  medianParam = median( paramSampleVec )
  dres = density( paramSampleVec )
  modeParam = dres$x[which.max(dres$y)]
  mcmcEffSz = round( effectiveSize( paramSampleVec ) , 1 )
  names(mcmcEffSz) = NULL
  hdiLim = HDIofMCMC( paramSampleVec , credMass=credMass )
  if ( !is.null(compVal) ) {
    pcgtCompVal = ( 100 * sum( paramSampleVec > compVal )
                    / length( paramSampleVec ) )
  } else {
    compVal=NA
    pcgtCompVal=NA
  }
  if ( !is.null(ROPE) ) {
    pcltRope = ( 100 * sum( paramSampleVec < ROPE[1] )
                 / length( paramSampleVec ) )
    pcgtRope = ( 100 * sum( paramSampleVec > ROPE[2] )
                 / length( paramSampleVec ) )
    pcinRope = 100-(pcltRope+pcgtRope)
  } else {
    ROPE = c(NA,NA)
    pcltRope=NA
    pcgtRope=NA
    pcinRope=NA
  }
  return( c( Mean=meanParam , Median=medianParam , Mode=modeParam ,
             ESS=mcmcEffSz ,
             HDImass=credMass , HDIlow=hdiLim[1] , HDIhigh=hdiLim[2] ,
             CompVal=compVal , PcntGtCompVal=pcgtCompVal ,
             ROPElow=ROPE[1] , ROPEhigh=ROPE[2] ,
             PcntLtROPE=pcltRope , PcntInROPE=pcinRope , PcntGtROPE=pcgtRope ) )
}

plotPost = function( paramSampleVec , cenTend=c("mode","median","mean")[1] ,
                     compVal=NULL, ROPE=NULL, credMass=0.95, HDItextPlace=0.7,
                     xlab=NULL , xlim=NULL , yaxt=NULL , ylab=NULL ,
                     main=NULL , cex=NULL , cex.lab=NULL ,
                     col=NULL , border=NULL , showCurve=FALSE , breaks=NULL ,
                     ... ) {
```

```
# Override defaults of hist function, if not specified by user:
# (additional arguments "..." are passed to the hist function)
if ( is.null(xlab) ) xlab="Param. Val."
if ( is.null(cex.lab) ) cex.lab=1.5
if ( is.null(cex) ) cex=1.4
if ( is.null(xlim) ) xlim=range( c( compVal , ROPE , paramSampleVec ) )
if ( is.null(main) ) main=""
if ( is.null(yaxt) ) yaxt="n"
if ( is.null(ylab) ) ylab=""
if ( is.null(col) ) col="skyblue"
if ( is.null(border) ) border="white"

# convert coda object to matrix:
if ( class(paramSampleVec) == "mcmc.list" ) {
  paramSampleVec = as.matrix(paramSampleVec)
}

summaryColNames = c("ESS","mean","median","mode",
                    "hdiMass","hdiLow","hdiHigh",
                    "compVal","pGtCompVal",
                    "ROPElow","ROPEhigh","pLtROPE","pInROPE","pGtROPE")
postSummary = matrix( NA , nrow=1 , ncol=length(summaryColNames) ,
                      dimnames=list( c( xlab ) , summaryColNames ) )

# require(coda) # for effectiveSize function
postSummary[,"ESS"] = effectiveSize(paramSampleVec)

postSummary[,"mean"] = mean(paramSampleVec)
postSummary[,"median"] = median(paramSampleVec)
mcmcDensity = density(paramSampleVec)
postSummary[,"mode"] = mcmcDensity$x[which.max(mcmcDensity$y)]

HDI = HDIofMCMC( paramSampleVec , credMass )
postSummary[,"hdiMass"]=credMass
postSummary[,"hdiLow"]=HDI[1]
postSummary[,"hdiHigh"]=HDI[2]

# Plot histogram.
cvCol = "darkgreen"
ropeCol = "darkred"
if ( is.null(breaks) ) {
  if ( max(paramSampleVec) > min(paramSampleVec) ) {
    breaks = c( seq( from=min(paramSampleVec) , to=max(paramSampleVec) ,
                     by=(HDI[2]-HDI[1])/18 ) , max(paramSampleVec) )
  } else {
    breaks=c(min(paramSampleVec)-1.0E-6,max(paramSampleVec)+1.0E-6)
    border="skyblue"
  }
```

```
}
if ( !showCurve ) {
  par(xpd=NA)
  histinfo = hist( paramSampleVec , xlab=xlab , yaxt=yaxt , ylab=ylab ,
                   freq=F , border=border , col=col ,
                   xlim=xlim , main=main , cex=cex , cex.lab=cex.lab ,
                   breaks=breaks , ... )
}
if ( showCurve ) {
  par(xpd=NA)
  histinfo = hist( paramSampleVec , plot=F )
  densCurve = density( paramSampleVec , adjust=2 )
  plot( densCurve$x , densCurve$y , type="l" , lwd=5 , col=col , bty="n" ,
        xlim=xlim , xlab=xlab , yaxt=yaxt , ylab=ylab ,
        main=main , cex=cex , cex.lab=cex.lab , ... )
}
cenTendHt = 0.9*max(histinfo$density)
cvHt = 0.7*max(histinfo$density)
ROPEtextHt = 0.55*max(histinfo$density)
# Display central tendency:
mn = mean(paramSampleVec)
med = median(paramSampleVec)
mcmcDensity = density(paramSampleVec)
mo = mcmcDensity$x[which.max(mcmcDensity$y)]
if ( cenTend=="mode" ){
  text( mo , cenTendHt ,
        bquote(mode==.(signif(mo,3))) , adj=c(.5,0) , cex=cex )
}
if ( cenTend=="median" ){
  text( med , cenTendHt ,
        bquote(median==.(signif(med,3))) , adj=c(.5,0) , cex=cex , col=cvCol )
}
if ( cenTend=="mean" ){
  text( mn , cenTendHt ,
        bquote(mean==.(signif(mn,3))) , adj=c(.5,0) , cex=cex )
}
# Display the comparison value.
if ( !is.null( compVal ) ) {
  pGtCompVal = sum( paramSampleVec > compVal ) / length( paramSampleVec )
  pLtCompVal = 1 - pGtCompVal
  lines( c(compVal,compVal) , c(0.96*cvHt,0) ,
         lty="dotted" , lwd=2 , col=cvCol )
  text( compVal , cvHt ,
        bquote( .(round(100*pLtCompVal,1)) * "% < " *
                .(signif(compVal,3)) * " < " *
                .(round(100*pGtCompVal,1)) * "%" ) ,
        adj=c(pLtCompVal,0) , cex=0.8*cex , col=cvCol )
  postSummary[,"compVal"] = compVal
```

```
    postSummary[,"pGtCompVal"] = pGtCompVal
  }
  # Display the ROPE.
  if ( !is.null( ROPE ) ) {
    pInROPE = ( sum( paramSampleVec > ROPE[1] & paramSampleVec < ROPE[2] )
                / length( paramSampleVec ) )
    pGtROPE = ( sum( paramSampleVec >= ROPE[2] ) / length( paramSampleVec ) )
    pLtROPE = ( sum( paramSampleVec <= ROPE[1] ) / length( paramSampleVec ) )
    lines( c(ROPE[1],ROPE[1]) , c(0.96*ROPEtextHt,0) , lty="dotted" , lwd=2 ,
           col=ropeCol )
    lines( c(ROPE[2],ROPE[2]) , c(0.96*ROPEtextHt,0) , lty="dotted" , lwd=2 ,
           col=ropeCol)
    text( mean(ROPE) , ROPEtextHt ,
          bquote( .(round(100*pLtROPE,1)) * "% < " * .(ROPE[1]) * " < " *
                  .(round(100*pInROPE,1)) * "% < " * .(ROPE[2]) * " < " *
                  .(round(100*pGtROPE,1)) * "%" ) ,
          adj=c(pLtROPE+.5*pInROPE,0) , cex=1 , col=ropeCol )

    postSummary[,"ROPElow"]=ROPE[1]
    postSummary[,"ROPEhigh"]=ROPE[2]
    postSummary[,"pLtROPE"]=pLtROPE
    postSummary[,"pInROPE"]=pInROPE
    postSummary[,"pGtROPE"]=pGtROPE
  }
  # Display the HDI.
  lines( HDI , c(0,0) , lwd=4 , lend=1 )
  text( mean(HDI) , 0 , bquote(.(100*credMass) * "% HDI" ) ,
        adj=c(.5,-1.7) , cex=cex )
  text( HDI[1] , 0 , bquote(.(signif(HDI[1],3))) ,
        adj=c(HDItextPlace,-0.5) , cex=cex )
  text( HDI[2] , 0 , bquote(.(signif(HDI[2],3))) ,
        adj=c(1.0-HDItextPlace,-0.5) , cex=cex )
  par(xpd=F)
  #
  return( postSummary )
}

#------------------------------------------------------------------------------

# Shape parameters from central tendency and scale:

betaABfromMeanKappa = function( mean , kappa ) {
  if ( mean <=0 | mean >= 1) stop("must have 0 < mean < 1")
  if ( kappa <=0 ) stop("kappa must be > 0")
  a = mean * kappa
  b = ( 1.0 - mean ) * kappa
  return( list( a=a , b=b ) )
}
```

```
betaABfromModeKappa = function( mode , kappa ) {
  if ( mode <=0 | mode >= 1) stop("must have 0 < mode < 1")
  if ( kappa <=2 ) stop("kappa must be > 2 for mode parameterization")
  a = mode * ( kappa - 2 ) + 1
  b = ( 1.0 - mode ) * ( kappa - 2 ) + 1
  return( list( a=a , b=b ) )
}

betaABfromMeanSD = function( mean , sd ) {
  if ( mean <=0 | mean >= 1) stop("must have 0 < mean < 1")
  if ( sd <= 0 ) stop("sd must be > 0")
  kappa = mean*(1-mean)/sd^2 - 1
  if ( kappa <= 0 ) stop("invalid combination of mean and sd")
  a = mean * kappa
  b = ( 1.0 - mean ) * kappa
  return( list( a=a , b=b ) )
}

gammaShRaFromMeanSD = function( mean , sd ) {
  if ( mean <=0 ) stop("mean must be > 0")
  if ( sd <=0 ) stop("sd must be > 0")
  shape = mean^2/sd^2
  rate = mean/sd^2
  return( list( shape=shape , rate=rate ) )
}

gammaShRaFromModeSD = function( mode , sd ) {
  if ( mode <=0 ) stop("mode must be > 0")
  if ( sd <=0 ) stop("sd must be > 0")
  rate = ( mode + sqrt( mode^2 + 4 * sd^2 ) ) / ( 2 * sd^2 )
  shape = 1 + mode * rate
  return( list( shape=shape , rate=rate ) )
}

#------------------------------------------------------------------------------

# Make some data files for examples...
createDataFiles=FALSE
if ( createDataFiles ) {

  source("HtWtDataGenerator.R")
  N=300
  m = HtWtDataGenerator( N , rndsd=47405 )
  write.csv( file=paste0("HtWtData",N,".csv") , row.names=FALSE , m )


  # Function for generating normal data with normal outliers:
```

```
genYwithOut = function( N , pcntOut=15 , sdOut=3.0 ) {
  inl = rnorm( N-ceiling(pcntOut/100*N) )
  out = rnorm(   ceiling(pcntOut/100*N) )
  inl = (inl-mean(inl))/sd(inl)
  out = (out-mean(out))/sd(out) * sdOut
  return(c(inl,out))
}

# Two-group IQ scores with outliers
set.seed(47405)
y1 = round(pmax(50,genYwithOut(63,20,3.5)*17.5+106))
y2 = round(pmax(50,genYwithOut(57,20,3.5)*10+100))
write.csv( file="TwoGroupIQ.csv" , row.names=FALSE ,
           data.frame( Score=c(y1,y2) ,
                       Group=c(rep("Smart Drug",length(y1)),
                               rep("Placebo",length(y2))) ) )

# One-group log-normal
set.seed(47405)
z = rnorm(123)
logY = (z-mean(z))/sd(z) * 0.5 + 5.5 # logY has mean 5.5 and sd 0.5
y = round( exp(logY) , 2 )
write.csv( file="OneGroupLogNormal.csv" , row.names=FALSE ,
           cbind(y) )

# One-group gamma
desiredMode = 250
desiredSD = 100
desiredRate = (desiredMode+sqrt(desiredMode^2+4*desiredSD^2))/(2*desiredSD^2)
desiredShape = 1+desiredMode*desiredRate
set.seed(47405)
y = round( rgamma( 153 , shape=desiredShape , rate=desiredRate ) , 2 )
write.csv( file="OneGroupGamma.csv" , row.names=FALSE , cbind(y) )

} # end if createDataFiles
```

3. writeLines(modelString , con="Jags-MultivariateNormal-model.txt")

```
model {
  for ( i in 1:Ntotal ) {
    zy[i,1:Nvar] ~ dmnorm( zMu[1:Nvar] , zInvCovMat[1:Nvar,1:Nvar] )
  }
  for ( varIdx in 1:Nvar ) { zMu[varIdx] ~ dnorm( 0 , 1/2^2 ) }
  zInvCovMat ~ dwish( zRmat[1:Nvar,1:Nvar] , zRscal )
  # Convert invCovMat to sd and correlation:
  zCovMat <- inverse( zInvCovMat )
  for ( varIdx in 1:Nvar ) { zSigma[varIdx] <- sqrt(zCovMat[varIdx,varIdx]) }
  for ( varIdx1 in 1:Nvar ) { for ( varIdx2 in 1:Nvar ) {
    zRho[varIdx1,varIdx2] <- ( zCovMat[varIdx1,varIdx2]
                               / (zSigma[varIdx1]*zSigma[varIdx2]) )
  } }
  # Convert to original scale:
  for ( varIdx in 1:Nvar ) {
    sigma[varIdx] <- zSigma[varIdx] * sdOrig[varIdx]
    mu[varIdx] <- zMu[varIdx] * sdOrig[varIdx] + meanOrig[varIdx]
  }
  for ( varIdx1 in 1:Nvar ) { for ( varIdx2 in 1:Nvar ) {
    rho[varIdx1,varIdx2] <- zRho[varIdx1,varIdx2]
  } }
}
```

S1: The complete correlation figures

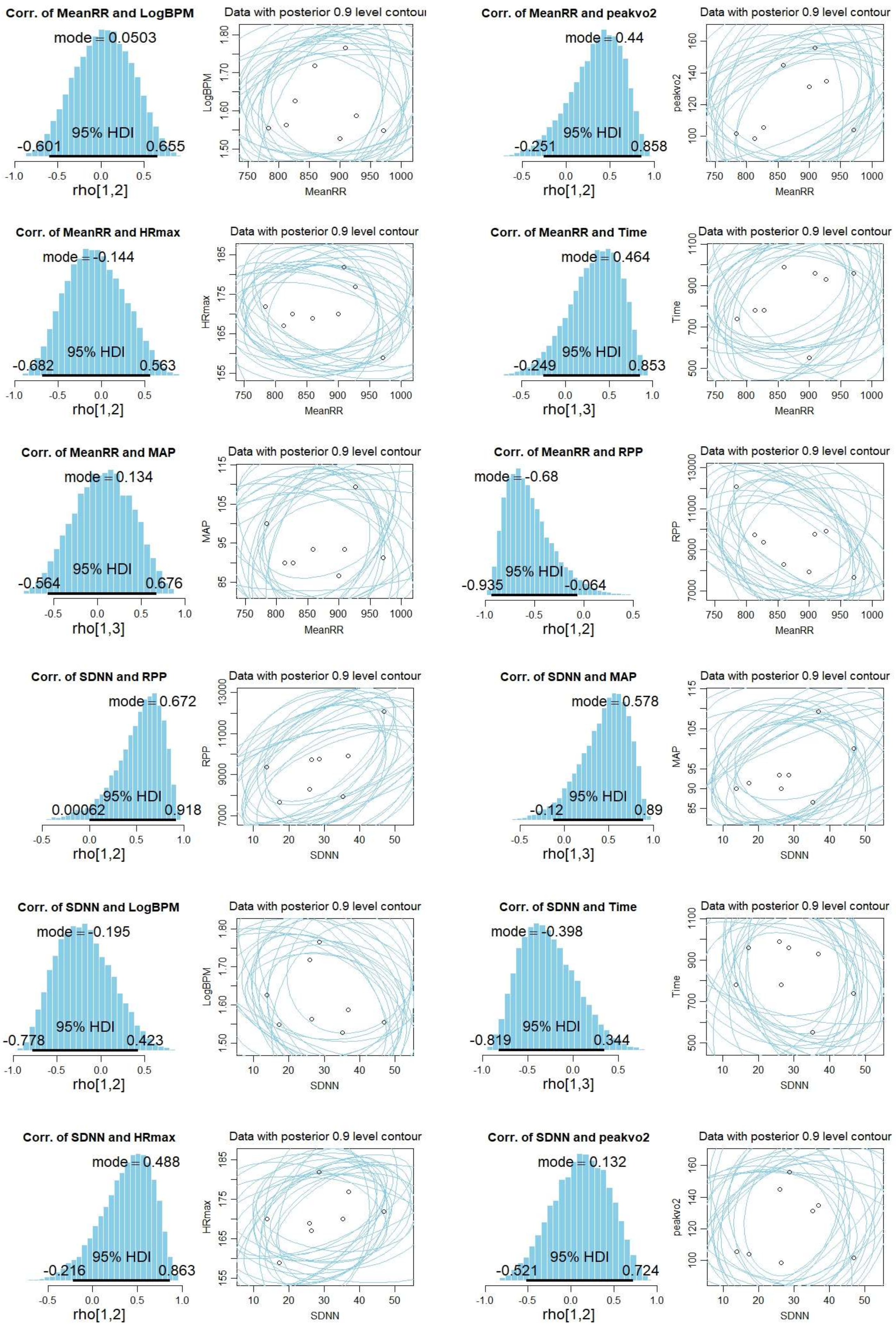

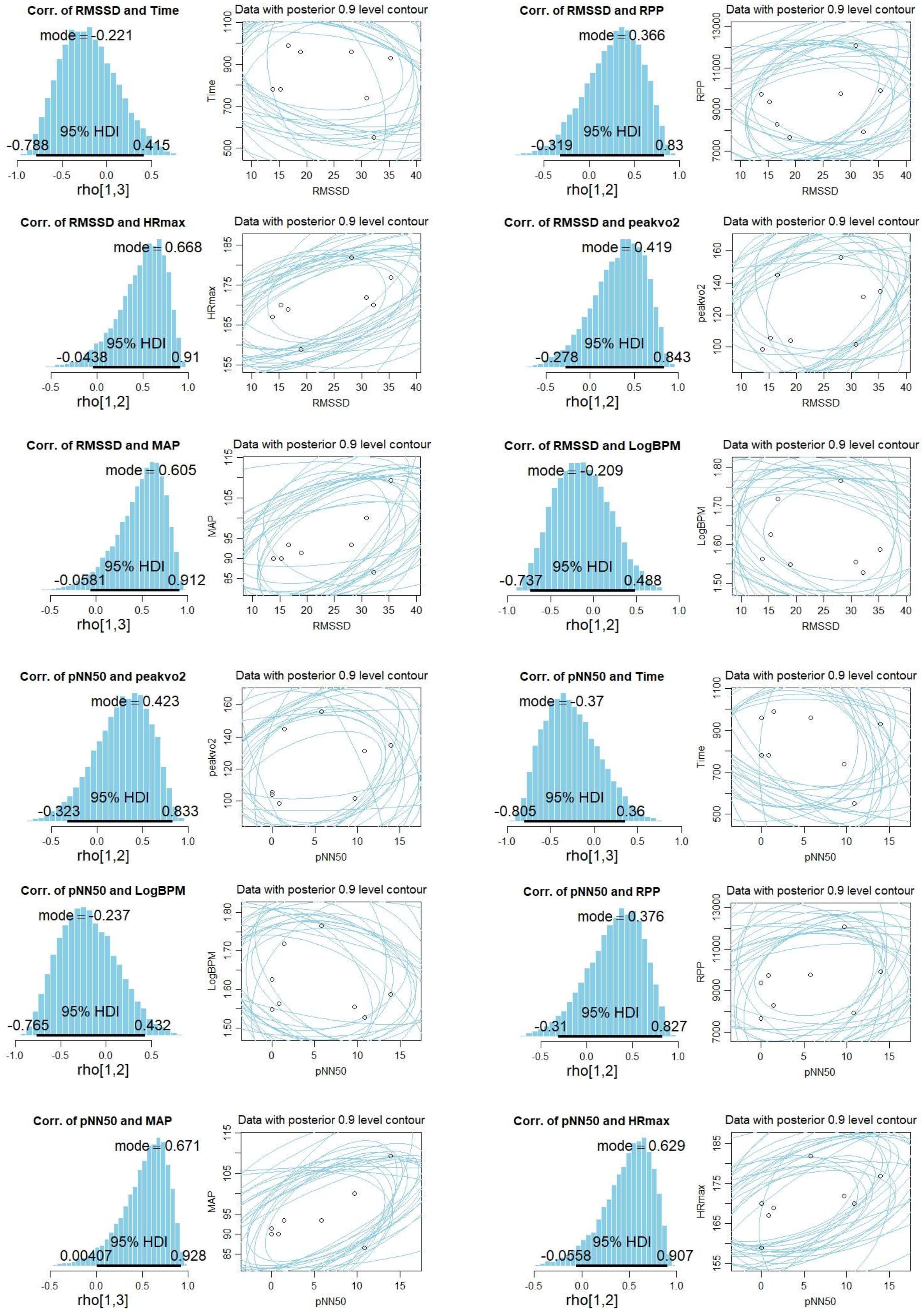

Corr. of RMSSD and Time
mode = -0.221
95% HDI
-0.788
0.415
rho[1,3]
Data with posterior 0.9 level contour
Time
RMSSD
Corr. of RMSSD and RPP
mode = 0.366
95% HDI
-0.319
0.83
rho[1,2]
Data with posterior 0.9 level contour
RPP
RMSSD
Corr. of RMSSD and HRmax
mode = 0.668
95% HDI
-0.0438
0.91
rho[1,2]
Data with posterior 0.9 level contour
HRmax
RMSSD
Corr. of RMSSD and peakvo2
mode = 0.419
95% HDI
-0.278
0.843
rho[1,2]
Data with posterior 0.9 level contour
peakvo2
RMSSD
Corr. of RMSSD and MAP
mode = 0.605
95% HDI
-0.0581
0.912
rho[1,3]
Data with posterior 0.9 level contour
MAP
RMSSD
Corr. of RMSSD and LogBPM
mode = -0.209
95% HDI
-0.737
0.488
rho[1,2]
Data with posterior 0.9 level contour
LogBPM
RMSSD
Corr. of pNN50 and peakvo2
mode = 0.423
95% HDI
-0.323
0.833
rho[1,2]
Data with posterior 0.9 level contour
peakvo2
pNN50
Corr. of pNN50 and Time
mode = -0.37
95% HDI
-0.805
0.36
rho[1,3]
Data with posterior 0.9 level contour
Time
pNN50
Corr. of pNN50 and LogBPM
mode = -0.237
95% HDI
-0.765
0.432
rho[1,2]
Data with posterior 0.9 level contour
LogBPM
pNN50
Corr. of pNN50 and RPP
mode = 0.376
95% HDI
-0.31
0.827
rho[1,2]
Data with posterior 0.9 level contour
RPP
pNN50
Corr. of pNN50 and MAP
mode = 0.671
95% HDI
0.00407
0.928
rho[1,3]
Data with posterior 0.9 level contour
MAP
pNN50
Corr. of pNN50 and HRmax
mode = 0.629
95% HDI
-0.0558
0.907
rho[1,2]
Data with posterior 0.9 level contour
HRmax
pNN50

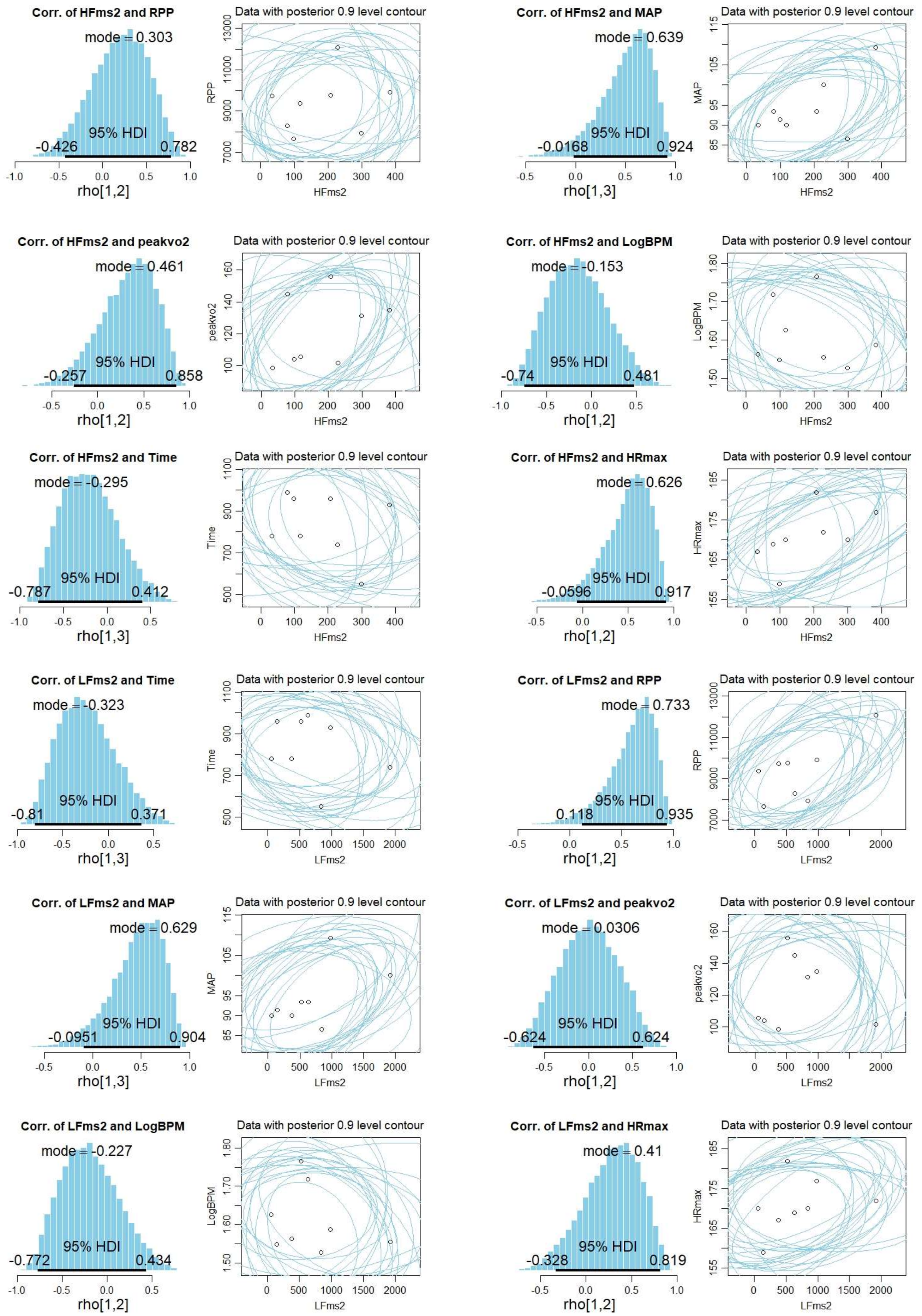
Corr. of HFms2 and RPP
mode = 0.303
95% HDI
-0.426
0.782
rho[1,2]
Data with posterior 0.9 level contour
RPP
HFms2
Corr. of HFms2 and MAP
mode = 0.639
95% HDI
-0.0168
0.924
rho[1,3]
Data with posterior 0.9 level contour
MAP
HFms2
Corr. of HFms2 and peakvo2
mode = 0.461
95% HDI
-0.257
0.858
rho[1,2]
Data with posterior 0.9 level contour
peakvo2
HFms2
Corr. of HFms2 and LogBPM
mode = -0.153
95% HDI
-0.74
0.481
rho[1,2]
Data with posterior 0.9 level contour
LogBPM
HFms2
Corr. of HFms2 and Time
mode = -0.295
95% HDI
-0.787
0.412
rho[1,3]
Data with posterior 0.9 level contour
Time
HFms2
Corr. of HFms2 and HRmax
mode = 0.626
95% HDI
-0.0596
0.917
rho[1,2]
Data with posterior 0.9 level contour
HRmax
HFms2
Corr. of LFms2 and Time
mode = -0.323
95% HDI
-0.81
0.371
rho[1,3]
Data with posterior 0.9 level contour
Time
LFms2
Corr. of LFms2 and RPP
mode = 0.733
95% HDI
0.118
0.935
rho[1,2]
Data with posterior 0.9 level contour
RPP
LFms2
Corr. of LFms2 and MAP
mode = 0.629
95% HDI
-0.0951
0.904
rho[1,3]
Data with posterior 0.9 level contour
MAP
LFms2
Corr. of LFms2 and peakvo2
mode = 0.0306
95% HDI
-0.624
0.624
rho[1,2]
Data with posterior 0.9 level contour
peakvo2
LFms2
Corr. of LFms2 and LogBPM
mode = -0.227
95% HDI
-0.772
0.434
rho[1,2]
Data with posterior 0.9 level contour
LogBPM
LFms2
Corr. of LFms2 and HRmax
mode = 0.41
95% HDI
-0.328
0.819
rho[1,2]
Data with posterior 0.9 level contour
HRmax
LFms2

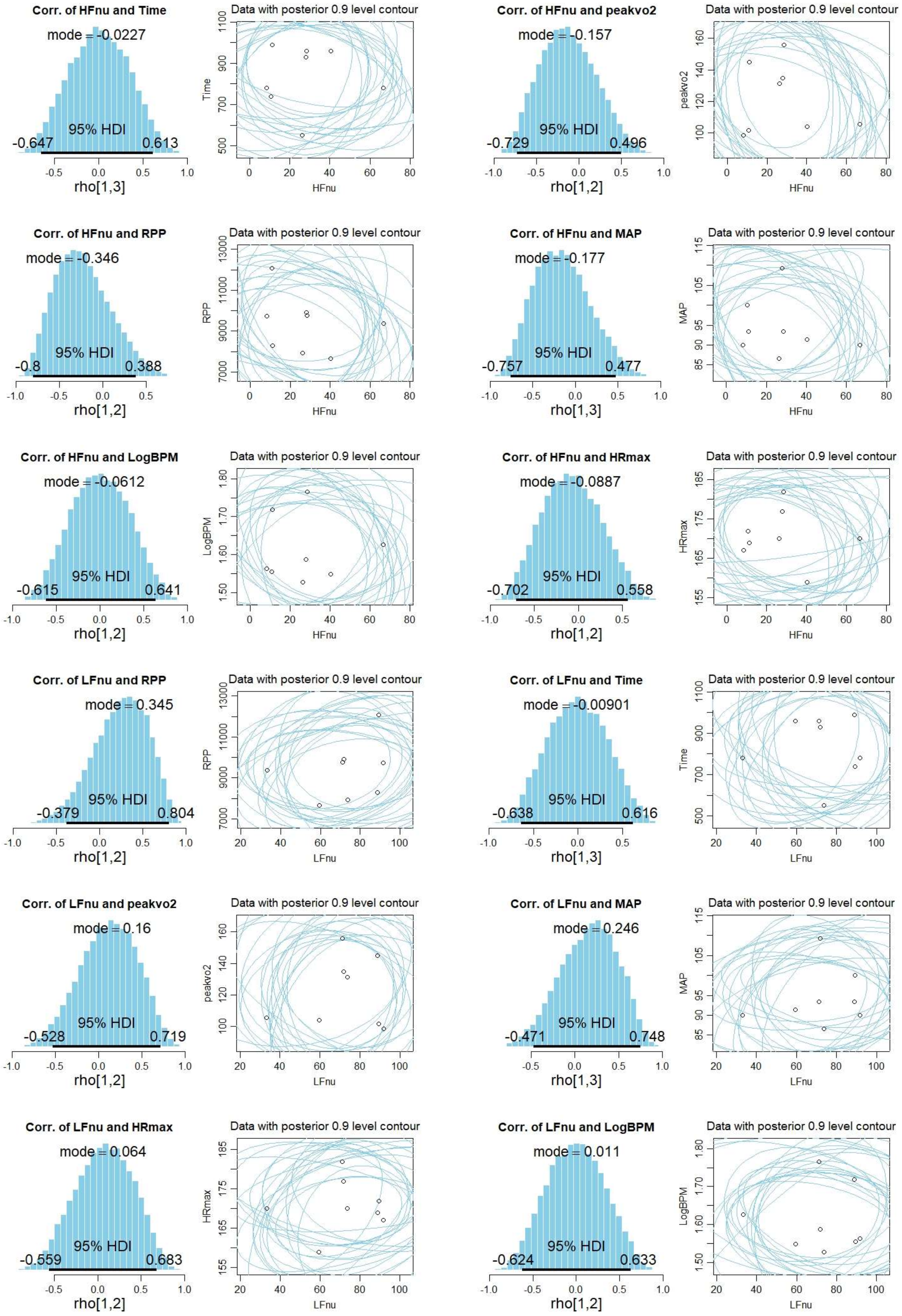

Corr. of HFnu and Time
mode = -0.0227
95% HDI
-0.647
0.613
rho[1,3]
Data with posterior 0.9 level contour
Time
HFnu
Corr. of HFnu and peakvo2
mode = -0.157
95% HDI
-0.729
0.496
rho[1,2]
Data with posterior 0.9 level contour
peakvo2
HFnu
Corr. of HFnu and RPP
mode = -0.346
95% HDI
-0.8
0.388
rho[1,2]
Data with posterior 0.9 level contour
RPP
HFnu
Corr. of HFnu and MAP
mode = -0.177
95% HDI
-0.757
0.477
rho[1,3]
Data with posterior 0.9 level contour
MAP
HFnu
Corr. of HFnu and LogBPM
mode = -0.0612
95% HDI
-0.615
0.641
rho[1,2]
Data with posterior 0.9 level contour
LogBPM
HFnu
Corr. of HFnu and HRmax
mode = -0.0887
95% HDI
-0.702
0.558
rho[1,2]
Data with posterior 0.9 level contour
HRmax
HFnu
Corr. of LFnu and RPP
mode = 0.345
95% HDI
-0.379
0.804
rho[1,2]
Data with posterior 0.9 level contour
RPP
LFnu
Corr. of LFnu and Time
mode = -0.00901
95% HDI
-0.638
0.616
rho[1,3]
Data with posterior 0.9 level contour
Time
LFnu
Corr. of LFnu and peakvo2
mode = 0.16
95% HDI
-0.528
0.719
rho[1,2]
Data with posterior 0.9 level contour
peakvo2
LFnu
Corr. of LFnu and MAP
mode = 0.246
95% HDI
-0.471
0.748
rho[1,3]
Data with posterior 0.9 level contour
MAP
LFnu
Corr. of LFnu and HRmax
mode = 0.064
95% HDI
-0.559
0.683
rho[1,2]
Data with posterior 0.9 level contour
HRmax
LFnu
Corr. of LFnu and LogBPM
mode = 0.011
95% HDI
-0.624
0.633
rho[1,2]
Data with posterior 0.9 level contour
LogBPM
LFnu

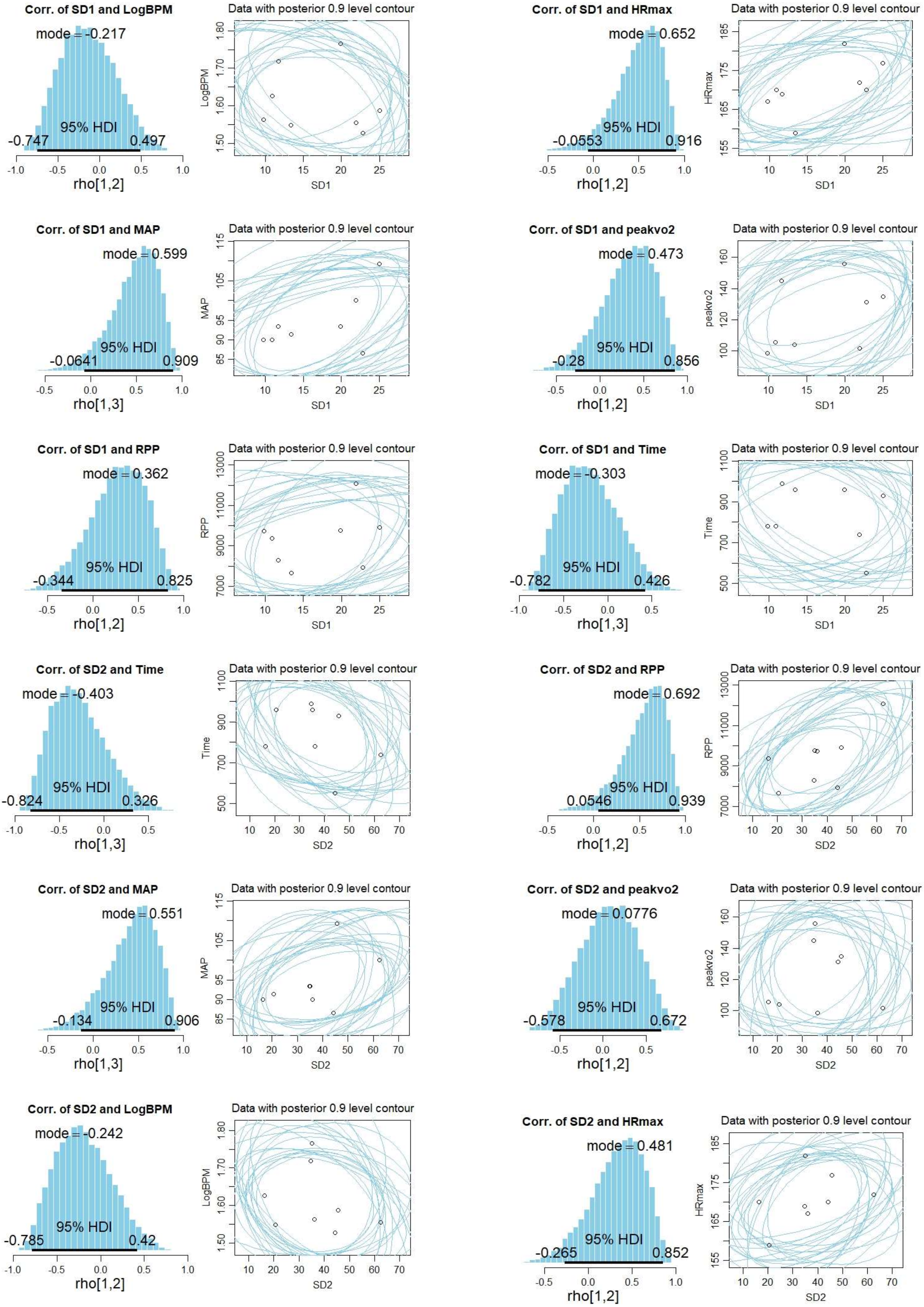
Corr. of SD1 and LogBPM
mode = -0.217
95% HDI
-0.747
0.497
rho[1,2]
Data with posterior 0.9 level contour
LogBPM
SD1
Corr. of SD1 and HRmax
mode = 0.652
95% HDI
-0.0553
0.916
rho[1,2]
Data with posterior 0.9 level contour
HRmax
SD1
Corr. of SD1 and MAP
mode = 0.599
95% HDI
-0.0641
0.909
rho[1,3]
Data with posterior 0.9 level contour
MAP
SD1
Corr. of SD1 and peakvo2
mode = 0.473
95% HDI
-0.28
0.856
rho[1,2]
Data with posterior 0.9 level contour
peakvo2
SD1
Corr. of SD1 and RPP
mode = 0.362
95% HDI
-0.344
0.825
rho[1,2]
Data with posterior 0.9 level contour
RPP
SD1
Corr. of SD1 and Time
mode = -0.303
95% HDI
-0.782
0.426
rho[1,3]
Data with posterior 0.9 level contour
Time
SD1
Corr. of SD2 and Time
mode = -0.403
95% HDI
-0.824
0.326
rho[1,3]
Data with posterior 0.9 level contour
Time
SD2
Corr. of SD2 and RPP
mode = 0.692
95% HDI
0.0546
0.939
rho[1,2]
Data with posterior 0.9 level contour
RPP
SD2
Corr. of SD2 and MAP
mode = 0.551
95% HDI
-0.134
0.906
rho[1,3]
Data with posterior 0.9 level contour
MAP
SD2
Corr. of SD2 and peakvo2
mode = 0.0776
95% HDI
-0.578
0.672
rho[1,2]
Data with posterior 0.9 level contour
peakvo2
SD2
Corr. of SD2 and LogBPM
mode = -0.242
95% HDI
-0.785
0.42
rho[1,2]
Data with posterior 0.9 level contour
LogBPM
SD2
Corr. of SD2 and HRmax
mode = 0.481
95% HDI
-0.265
0.852
rho[1,2]
Data with posterior 0.9 level contour
HRmax
SD2
